# Selective electroreduction of CO_2_ to acetone by single copper atoms anchored on N-doped porous carbon

**DOI:** 10.1038/s41467-020-16381-8

**Published:** 2020-05-15

**Authors:** Kun Zhao, Xiaowa Nie, Haozhi Wang, Shuo Chen, Xie Quan, Hongtao Yu, Wonyong Choi, Guanghui Zhang, Bupmo Kim, Jingguang G. Chen

**Affiliations:** 1 Key Laboratory of Industrial Ecology and Environmental Engineering (Ministry of Education, China), School of Environmental Science and Technology, Dalian University of Technology, Dalian, 116024 PR China; 2 State Key Laboratory of Fine Chemicals, PSU-DUT Joint Center for Energy Research, School of Chemical Engineering, Dalian University of Technology, Dalian, 116024 PR China; 3 Department of Chemical Engineering, Columbia University, New York, NY 10027 USA; 4 Division of Environmental Science and Engineering, Pohang University of Science and Technology, Pohang, 37673 Republic of Korea

**Keywords:** Catalyst synthesis, Energy, Density functional theory, Electrocatalysis

## Abstract

Efficient electroreduction of CO_2_ to multi-carbon products is a challenging reaction because of the high energy barriers for CO_2_ activation and C–C coupling, which can be tuned by designing the metal centers and coordination environments of catalysts. Here, we design single atom copper encapsulated on N-doped porous carbon (Cu-SA/NPC) catalysts for reducing CO_2_ to multi-carbon products. Acetone is identified as the major product with a Faradaic efficiency of 36.7% and a production rate of 336.1 μg h^−1^. Density functional theory (DFT) calculations reveal that the coordination of Cu with four pyrrole-N atoms is the main active site and reduces the reaction free energies required for CO_2_ activation and C–C coupling. The energetically favorable pathways for CH_3_COCH_3_ production from CO_2_ reduction are proposed and the origin of selective acetone formation on Cu-SA/NPC is clarified. This work provides insight into the rational design of efficient electrocatalysts for reducing CO_2_ to multi-carbon products.

## Abstract (web summary)

Efficient electroreduction of CO_2_ to multi-carbon products is challenging. Here, the single atom Cu encapsulated on N-doped porous carbon catalysts are designed for reducing CO_2_ to acetone at low overpotentials and the active sites are identified as Cu coordination with four pyrrole-N atoms.

## Introduction

Electrochemical reduction of CO_2_ to value-added chemicals and fuels offers a promising approach for solving issues related to energy crisis and global warming^1–5^. CO_2_ can be converted to C_1_, C_2_, or C_2+_ products via different proton- and electron-transfer steps and different CO_2_ reduction pathways. Converting CO_2_ to multi-carbon (C_2_ and C_2+_) products is more desirable due to their higher value and higher energy density^6–9^.

Recently, numerous electrocatalysts have been designed for CO_2_ reduction, such as metals, metal oxides, and carbon-based materials^10–14^. Among these catalysts, copper (Cu) owns the ability to generate multi-carbon products from CO_2_ reduction. This may be related to the optimal binding energy of CO intermediate on Cu, leading to the further reduction of CO intermediate and achieving the C–C coupling^12,15–17^. Although Cu-based catalysts can reduce CO_2_ to C_2_ products, it still suffers from high barriers for CO_2_ activation and C–C coupling, resulting in large overpotentials for C_2_ product formation^18–21^. Moreover, efficient reduction of CO_2_ to C_2+_ products, such as C_3_ oxygenates, on Cu-based electrocatalysts are rarely reported.

Single atom catalysts (SAC) with atomically distributed active metal centers have been demonstrated to possess enhanced activity and tunable selectivity toward CO_2_ reduction due to its maximum atom utilization efficiency, unique electronic structure, and unsaturated coordination environment of metal centers^22–24^. As reported previously, partially oxidized single atom cobalt can electrochemically reduce CO_2_ to HCOOH with a Faradaic efficiency of ~90%^25^. Isolated Ni centers in N- and S-doped graphene exhibit high activity toward CO production with a TOF value of 14,800 h^−1^ and a maximum Faradaic efficiency of 97% at −0.5 V^26^. Single Fe(II) sites on N-doped carbon are active for reducing CO_2_ to CH_3_COOH^27^. These results have clearly demonstrated the potential in using single atom materials as active and selective electrocatalysts for CO_2_ reduction.

In this work, the atomically distributed Cu is anchored on N-doped porous carbon (Cu-SA/NPC) and is evaluated for CO_2_ electrochemical reduction. The Cu-SA/NPC reduces CO_2_ to acetic acid, ethanol, and acetone products at a low overpotential, with acetone being the major product. The effects of Cu distribution and local coordination environment of SAC on CO_2_ reduction are investigated. The active sites of Cu-SA/NPC and mechanisms of CO_2_ activation, C–C coupling, and CH_3_COCH_3_ formation from CO_2_ reduction are elucidated by combined experimental and density functional theory (DFT) studies.

## Results

### Characterization of Cu-SA/NPC

The Cu-SA/NPC was synthesized by a continuous process including hydrothermal synthesis of Cu-doped ZIF-8 and subsequent carbonization of the precursor at 1000 °C under N_2_ atmosphere. The elemental composition of obtained catalysts was characterized by XPS measurement. The NPC only showed the signal of C, N, and O elements (Supplementary Fig. 1), while the Cu-SA/NPC exhibited a small peak of Cu, indicating that Cu was successfully incorporated in the prepared material. The Cu content was determined to be about 0.59 wt% by inductively coupled plasma atomic emission spectroscopy (ICP-AES) (Supplementary Table 1). The XRD patterns of Cu-SA/NPC showed two broad diffraction peaks located at 23° and 44°, corresponding to the (002) and (101) plane of carbon, respectively (Supplementary Fig. 2). No Cu-related crystal phases were observed, which might be caused by the low loading amount of Cu species.

**Fig. 1:**
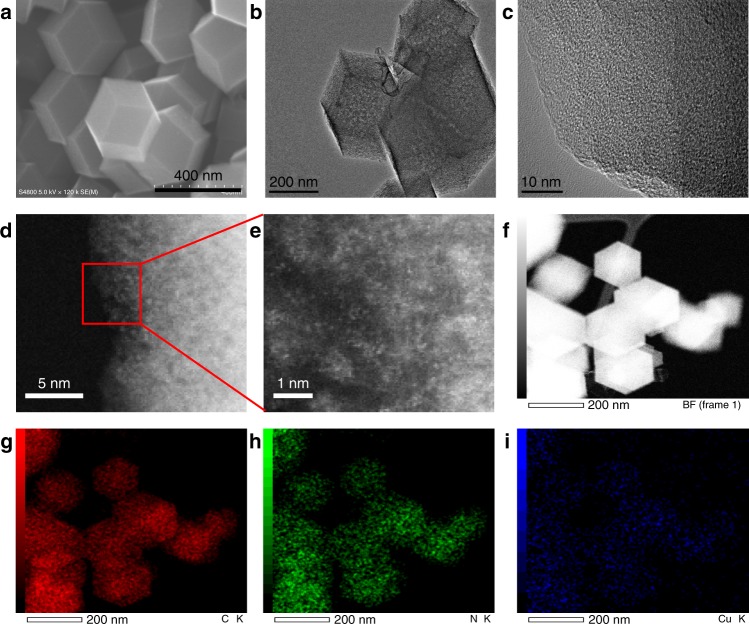
Morphological characterization of Cu-SA/NPC. **a** SEM. **b**, **c** TEM. **d**, **e** HAADF-STEM images and enlarged images. **f**–**i** EDS images of Cu-SA/NPC by HAADF-STEM.

As shown in the scanning electron microscope (SEM) and transmission electron microscope (TEM) images, Cu-SA/NPC retained the rhombic dodecahedral morphology of the pristine ZIF precursor (Fig. 1 and Supplementary Fig. 3), while exhibiting a rough surface. It is worth noting that no Cu nanoparticles or large clusters were observed in the SEM and TEM images of Cu-SA/NPC catalysts. The presence of Cu on Cu-SA/NPC was confirmed by atomic-resolution high-angle annular dark-field scanning TEM (HAADF-STEM). Fig. 1d, e clearly showed the presence of bright dots that were attributed to the atomically distributed Cu components. The particle size of Cu was measured to be about 0.1 nm, revealing that Cu on Cu-SA/NPC catalysts was present at atom dimension. Energy dispersive X-ray spectroscopy (EDS) mapping analysis in STEM images confirmed the uniform dispersion of Cu, N, and C species in Cu-SA/NPC (Fig. 1f–i and Supplementary Fig. 4), demonstrating that Cu atom was homogeneously distributed on N-doped carbon materials.

**Fig. 2:**
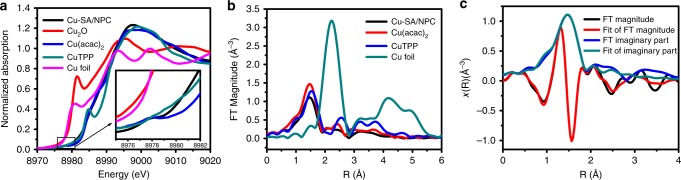
Structural characterization of Cu-SA/NPC. **a** Normalized XANES spectra at Cu K-edge of Cu-SA/NPC, Cu_2_O, cupric acetylacetonate (Cu(acac)_2_), copper meso-tetraphenylporphine (CuTPP), and Cu foil. **b** Fourier transforms (FT) magnitude of the experimental EXAFS spectra of Cu-SA/NPC, Cu(acac)_2_, CuTPP, and Cu foil. **c** EXAFS R space fitting curves of Cu-SA/NPC.

The oxidation state and structural information of Cu-SA/NPC were characterized using X-ray absorption near edge spectroscopy (XANES) and extended X-ray absorption fine structure (EXAFS). Fig. 2a depicts the normalized XANES spectra of Cu-SA/NPC and several reference materials, such as Cu_2_O, cupric acetylacetonate (Cu(acac)_2_), copper meso-tetraphenylporphine (CuTPP), and Cu foil. The white-line intensities in these spectra were related to the oxidation states of Cu species. The white-line intensity of Cu-SA/NPC was similar to that of Cu(acac)_2_ and CuTPP, indicating that the Cu atoms existed as Cu(II) species. The pre-edge region was also indicative of the Cu oxidation state, as reported earlier that Cu(II) showed a small pre-edge feature while the pre-edge feature was absent for Cu or Cu(I)^28^. As shown in Fig. 2a, a small pre-edge feature was observed for the Cu-SA/NPC sample, confirming the Cu(II) oxidation state in Cu-SA/NPC. The Fourier-transformed EXAFS spectra of Cu-SA/NPC showed a main peak at ~1.5 Å, which could be assigned to the Cu–N bond. Notably, the peak related to Cu–Cu bond at ~2.2 Å was absent (Fig. 2b), consistent with the presence of individually distributed Cu. According to the EXAFS fitting results (Fig. 2c and Supplementary Table 2), the Cu atom coordinated with N atom and the coordination number was 3.8 ± 0.2. Based on the combined HAADF-STEM and EXAFS results, it could be concluded that the Cu species was atomically dispersed and was fourfold coordinated with N atoms in Cu-SA/NPC^29^.

### Electrochemical reduction of CO_2_ on Cu-SA/NPC

**Fig. 3:**
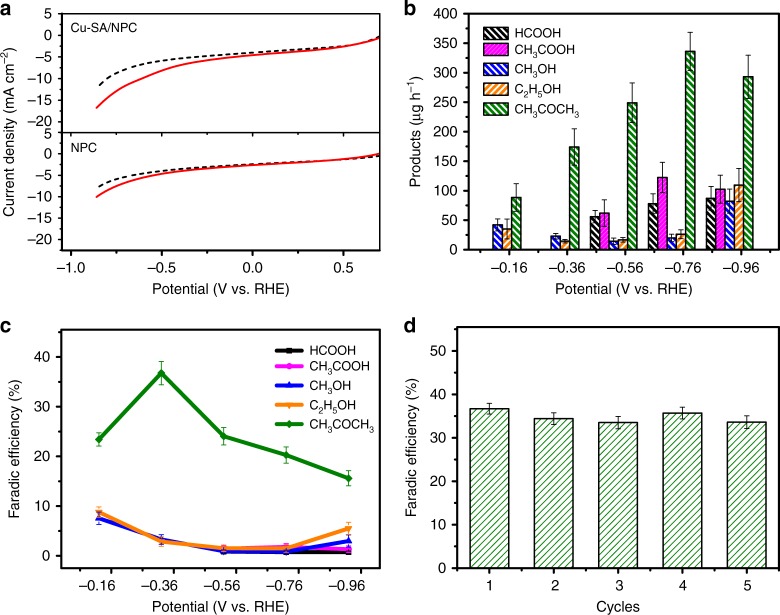
Catalytic performance of Cu-SA/NPC. **a** LSV curves of NPC and Cu-SA/NPC. **b** Production rate of CO_2_ reduction products on Cu-SA/NPC. **c** Faradaic efficiency of CO_2_ reduction products on Cu-SA/NPC. **d** Stability of Cu-SA/NPC.

The CO_2_ reduction activity of Cu-SA/NPC was investigated by linear sweep voltammetry (LSV) measurements. The LSV tests were performed in a phosphate buffer (0.2 M, pH 6.8) saturated with CO_2_ or Ar. As shown in Fig. 3a, the current density in CO_2_-saturated solution was greater than that in Ar-saturated solution for both NPC and Cu-SA/NPC, demonstrating that NPC and Cu-SA/NPC were active for CO_2_ electrochemical reduction. The current density for CO_2_ reduction on Cu-SA/NPC was much higher than that on NPC. Moreover, the onset potential (CO_2_ reduction current density achieved 1.0 mA cm^−2^) for CO_2_ reduction on Cu-SA/NPC was −0.25 V, which was more positive than that on NPC (−0.62 V). These results suggested that the incorporation of single atom Cu into NPC material could significantly enhance its catalytic activity toward CO_2_ reduction.

Electrochemical reduction of CO_2_ was performed in a CO_2_-saturated 0.1 M KHCO_3_ solution (pH 6.8) at applied potential of −0.16 to −0.96 V versus reversible hydrogen electrode (RHE). The detected liquid products on Cu-SA/NPC were formic acid (HCOOH), acetic acid (CH_3_COOH), methanol (CH_3_OH), ethanol (C_2_H_5_OH), and acetone (CH_3_COCH_3_), while the gaseous products were H_2_ and CO. Fig. 3b showed the product distribution on Cu-SA/NPC, which was found to be dependent on the applied potential (its current–time curves were presented in Supplementary Fig. 5). It is worthy to note that oxygenate products were generated at a low potential of −0.16 V versus RHE, revealing that the overpotential for CH_3_OH, C_2_H_5_OH, and CH_3_COCH_3_ production on Cu-SA/NPC was about 180, 250, and 250 mV, respectively. Among these reduction products, the CH_3_COCH_3_ was the major reduced product of CO_2_ reduction on the Cu-SA/NPC catalyst at low potential. Moreover, the production rate of CH_3_COCH_3_ was further investigated in the potentials between −0.1 and −1.0 V (Supplementary Fig. 6). At the potential of −0.1 V, no CH_3_COCH_3_ was detected. The production rate of CH_3_COCH_3_ increased at potentials between −0.16 and −0.76 V, and then decreased with the applied potential further negatively shifted to −1.0 V, likely due to the competing reaction of H_2_ evolution. The maximum CH_3_COCH_3_ production rate reached 336.1 μg h^−1^ (−0.76 V), which was 4.3, 2.8, 16.9, and 12.8 times larger than that of HCOOH, CH_3_COOH, CH_3_OH, and C_2_H_5_OH, respectively. No liquid products were detected during the electrolysis in Ar-saturated solution on Cu-SA/NPC catalysts, confirming that the oxygenates were generated from CO_2_ electrochemical reduction (Supplementary Fig. 7). As shown in Supplementary Fig. 8, no peak associated with acetone was detected from ^1^H NMR spectra after reducing HCOOH or CH_3_COOH in 0.1 M KHCO_3_ electrolyte, suggesting that the formed HCOOH and CH_3_COOH products could not be further reduced to acetone on Cu-SA/NPC. The isotope experiment using ^13^CO_2_ gas as carbon source was conducted and products were analyzed by GC-MS (Supplementary Fig. 9). The peaks at *m/z* = 59 and *m/z* = 60 were characteristic peak of ^13^C-labeled CH_3_COCH_3_, which confirmed that the gas ^13^CO_2_ was reduced on Cu-SA/NPC catalyst. The peak at *m/z* = 61 was not observed, which may be caused by the participation of HCO_3_^−^ electrolyte in CO_2_ reduction and it was evidenced by the isotope experiment using H^13^CO_3_^−^ (Supplementary Fig. 10). As shown in Supplementary Fig. 11, the characteristic peaks of CH_3_OH (*m/z* = 31 and *m/z* = 32), and C_2_H_5_OH (*m/z* = 27, *m/z* = 28, and *m/z* = 29) were detected in ^13^CO_2_ and H^13^CO_3_. These results were consistent with those reported in the literature^30,31^. According to the previous studies, there should be a dynamic equilibrium between CO_2_ partial pressure and HCO_3_^−^ concentration and the CO_2_ in equilibrium with HCO_3_^−^ was the source for CO_2_ reduction reaction^30,31^.

Energy efficiency was a major consideration for converting CO_2_ into chemicals by the electrochemical method, and thus the Faradaic efficiency for CO_2_ reduction on Cu-SA/NPC was investigated. As shown in Fig. 3c, the Faradaic efficiency of CH_3_COCH_3_ generation was 15.6–36.7% at the applied potential range of −0.16 to −0.96 V, which was much higher than those of other oxygenate products at tested potentials. With the applied potential negatively shifted, the Faradaic efficiency of CH_3_COCH_3_ generation increased and reached to a maximum value of 36.7% at −0.36 V, which was 11.2 and 12.7 times as great as those for CH_3_OH and C_2_H_5_OH, respectively. Both the production rate and Faradaic efficiency of acetone generation on Cu-SA/NPC were significantly enhanced as compared with other electrocatalysts reported in the literature^32,33^ (Supplementary Table 3). The stability of the Cu-SA/NPC catalyst was measured by five sequential CO_2_ electroreduction experiments at −0.36 V (Fig. 3d). The Faradaic efficiency for CH_3_COCH_3_ generation remained at ~36.7% for each cycle. Furthermore, the catalysts were characterized by SEM and HAADF-STEM (Supplementary Fig. 12) after five sequential CO_2_ reduction. The rhombic dodecahedral morphology of the post-reaction catalysts was intact. The HAADF-STEM results showed that Cu remained atomically distributed in porous carbon. These results indicated that the Cu-SA/NPC catalyst showed good stability in CO_2_ electrochemical reduction.

The reduced products for the electrochemical reduction of CO_2_ on NPC were measured for comparison. As shown in Supplementary Fig. 13, the products on NPC were detected to be HCOOH and CH_3_COOH at tested potentials. The trace amount of Zn impurity should have a negligible effect on acetone production from CO_2_ reduction on Cu-SA/NPC, as discussed in the Supplementary Note 1. Such comparison suggested that the production of CH_3_OH, C_2_H_5_OH, and CH_3_COCH_3_ on Cu-SA/NPC was attributed to the presence of single atom Cu. The Cu-SA/NPC catalysts with different Cu content were prepared. The Cu-SA/NPC catalyst was synthesized with the Zn/Cu ratio of 10/1. The catalysts with Zn/Cu ratios of 20/1, 5/1, 10/3, and 5/3 were also synthesized and denoted as Cu-SA/NPC_0.5_, Cu-SA/NPC_2_, Cu-SA/NPC_3_, and Cu-SA/NPC_6_, respectively. As shown in Supplementary Fig. 14, the Cu-SA/NPC_0.5_, Cu-SA/NPC_2_, and Cu-SA/NPC_3_ retained the rhombic dodecahedral morphology of pristine ZIF-8, and the size of prepared catalysts increased with increasing Cu content. The pristine rhombic dodecahedral morphology was destroyed when further increased the Cu content to a Zn/Cu ratio of 5/3 (Cu-SA/NPC_6_). Therefore, the CO_2_ electroreduction was conducted on Cu-SA/NPC_0.5_, Cu-SA/NPC, Cu-SA/NPC_2_, and Cu-SA/NPC_3_ at −0.76 V (Supplementary Fig. 15), with a acetone production rate of 108.6, 336.1, 194.7, and 129.3 μg h^−1^, respectively. The Faradic efficiency of acetone generation followed the same trend as Cu-SA/NPC > Cu-SA/NPC_2_ > Cu-SA/NPC_3_ > Cu-SA/NPC_0.5_. According to the XPS results, the Cu content of Cu-SA/NPC_0.5_, Cu-SA/NPC, Cu-SA/NPC_2_, and Cu-SA/NPC_3_ catalysts increased from 0.1 to 0.3% (Supplementary Table 4). However, the N content also increased slightly from 8.3 to 8.4% as the Zn/Cu ratio increased to 10/1, and the N content decreased to 6.4% with further increasing the Zn/Cu ratio to 10/3 (Supplementary Figs. 16, 17 and Table 4). The trend in the change of N content with the doping content of Cu metal was similar to that reported in the literature^34^. Therefore, the acetone production from CO_2_ on Cu-SA/NPC was not only related to the atomic Cu doping, but also related to the N content and species.

**Fig. 4:**
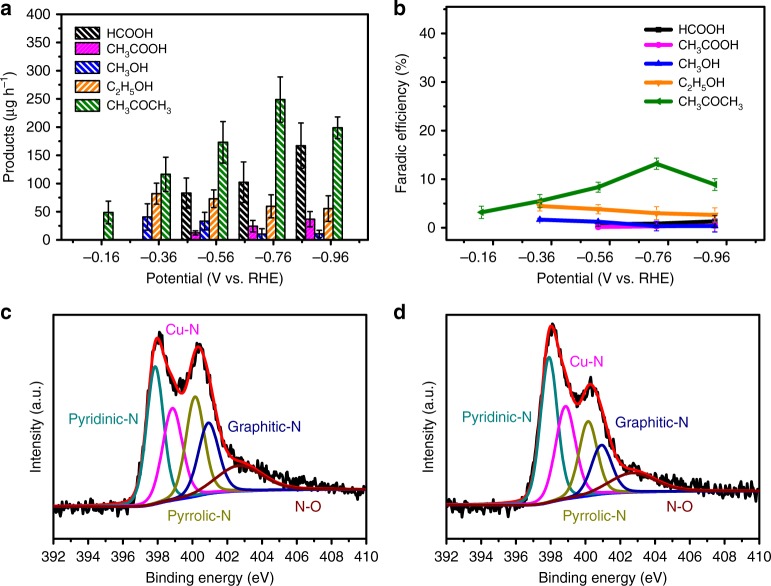
Catalytic performance of Cu-SA/NPCAr and high-resolution XPS. **a** Production rate of CO_2_ reduction products on Cu-SA/NPC_Ar_. **b** Faradaic efficiency of CO_2_ reduction products on Cu-SA/NPC_Ar_. **c** XPS N 1 s spectra of Cu-SA/NPC. **d** XPS N 1s spectra of Cu-SA/NPC_Ar_.

In order to determine whether the interaction between Cu and supported materials would be influenced by the carbonization conditions of MOFs, the Cu catalyst was prepared under Ar atmosphere and denoted as Cu-SA/NPC_Ar_. According to the LSV results (Supplementary Fig. 18), the onset potential for CO_2_ reduction on Cu-SA/NPC (−0.25 V) was more positive than that on Cu-SA/NPC_Ar_ (−0.38 V), indicating that CO_2_ reduction occurred more easily on the Cu-SA/NPC electrocatalyst. The production rate and Faradaic efficiency of reduced products from CO_2_ reduction on Cu-SA/NPC_Ar_ were also measured for comparison (Fig. 4a, b). At the applied potential of −0.76 V, the CH_3_COCH_3_ production rate on Cu-SA/NPC was about 336.1 μg h^−1^ with a Faradaic efficiency of 20.3%, while Cu-SA/NPC_Ar_ exhibited lower CH_3_COCH_3_ production rate (248.8 μg h^−1^) and Faradaic efficiency (13.2%), indicating that Cu-SA/NPC possessed a better CO_2_ reduction activity than Cu-SA/NPC_Ar_. Since the Cu-SA/NPC and Cu-SA/NPC_Ar_ catalysts had similar Cu content, BET surface area, and pore structure (Supplementary Tables 1, 5 and Figs. 19, 20), the different CO_2_ reduction activity may be resulted from the content and type of N species. The N content was characterized by X-ray photoelectron spectroscopy (XPS) and the N composition was determined by the peak area ratio of different N species (details provided in the Supplementary methods). Based on the XPS results, the Cu-SA/NPC and Cu-SA/NPC_Ar_ showed a similar total N content, while the distribution of N species was different (Supplementary Table 4). Fig. 4c, d showed the XPS N 1s spectra of Cu-SA/NPC and Cu-SA/NPC_Ar_, respectively. Both spectra exhibited five peaks with binding energies at 397.9, 398.9, 400.2, 401.0, and 402.7 eV, which were assigned to pyridinic N, metal Cu–N, pyrrolic N, graphite N, and oxidized N (N–O), respectively^35,36^. The content of graphite N and metal Cu–N was similar, while that of pyridinic N, pyrrolic N, and oxidized N (N–O) was different. As shown in Supplementary Table 4, the NPC possessed the oxidized N content of 1.1%, similar to that of Cu-SA/NPC (1.2%). However, there was no acetone production from CO_2_ reduction on NPC without Cu (Supplementary Fig. 13), suggesting the oxidized N alone might not play an important role in acetone production. As shown in Supplementary Table 4, the content of pyridinic N and pyrrolic N on Cu-SA/NPC was 2.2% and 1.8%, respectively, while that on Cu-SA/NPC_Ar_ was 2.7% and 1.4%, respectively. This comparison indicated that the Cu-SA/NPC had a higher content of pyrrolic N than that of Cu-SA/NPC_Ar_, which might play an important role for acetone production on Cu-SA/NPC. In view of CO_2_ reduction measurements and XPS results, the CH_3_COCH_3_ production from CO_2_ reduction might be determined by the single atom Cu and the content of pyrrolic N. However, how these two species combined with each other was not confirmed based on experimental results and DFT calculations were conducted to identify the active sites of Cu-SA/NPC and to uncover the mechanisms for acetone production from CO_2_ reduction.

### Active sites identification and mechanisms

**Fig. 5:**
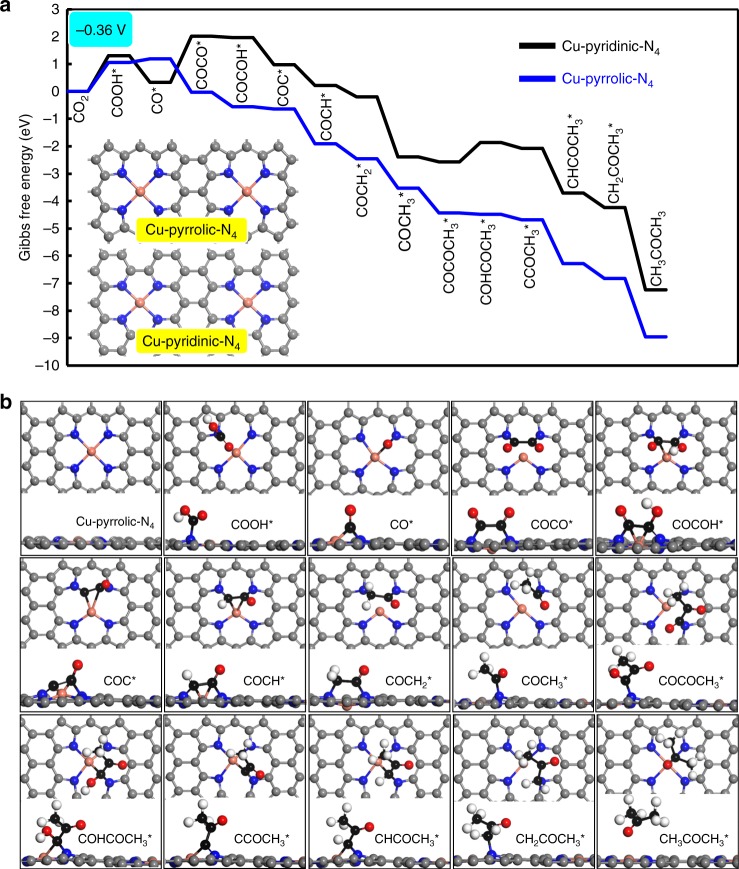
DFT calculations of reaction pathways on different Cu-N sites of Cu-SA/NPC. **a** Free energy diagrams calculated at a potential of −0.36 V for CO_2_ reduction to CH_3_COCH_3_ on Cu-pyridinic-N_4_ and Cu-pyrrolic-N_4_ sites of Cu-SA/NPC (the computational models were included in the figure). **b** Optimized structures of all reaction intermediates involved in the pathways of CO_2_ reduction on the Cu-pyrrolic-N_4_ site (gray: C of catalyst; black: C of adsorbate; red: O; orange: Cu; blue: N; white: H).

As the electrocatalytic activity of SAC could be affected by the coordination environment of isolated metal atoms^37^, different types of coordinated N species were first examined. According to the combined HAADF-STEM and EXAFS results, the Cu species should be atomically dispersed and was fourfold coordinated with N atoms in the Cu-SA/NPC^29^ catalyst. Herein, two catalyst models were constructed, in which single atom Cu was doped into a graphitic sheet with coordination environments with four pyridine N atoms (Cu-pyridinic-N_4_ site) and four pyrrole N atoms (Cu-pyrrolic-N_4_ site) (inside the Fig. 5a). The Gibbs free energy diagrams of CO_2_ reduction and Δ*G* values of elementary steps involved were calculated based on the computational hydrogen electrode model. Fig. 5a illustrated the lowest energy pathways of CH_3_COCH_3_ formation from CO_2_ reduction on Cu-pyridinic-N_4_ and Cu-pyrrolic-N_4_ sites of Cu-SA/NPC at a potential of −0.36 V versus RHE. The free energy diagrams at 0 V on the two sites were provided in Supplementary Fig. 21. Fig. 5b showed the optimized structures of all states generated on the Cu-pyrrolic-N_4_ site of Cu-SA/NPC while the optimized structures formed on Cu-pyridinic-N_4_ were provided in Supplementary Fig. 22. The proposed path went through in the sequence of CO_2_ → COOH* → CO* → COCO* → COCOH* → COC* → COCH* → COCH_2_* → COCH_3_* → COCOCH_3_* → COHCOCH_3_* → CCOCH_3_* → CHCOCH_3_* → CH_2_COCH_3_* → CH_3_COCH_3_. The free energy change for the overall process of CH_3_COCH_3_ production from CO_2_ reduction was negative, indicating that reducing CO_2_ to CH_3_COCH_3_ was thermodynamically favorable on the Cu-SA/NPC catalyst. The elementary steps for CO_2_ reduction to CH_3_COCH_3_ were more thermodynamically downhill when a −0.36 V potential was applied (comparing Fig. 5a and Supplementary Fig. 21), indicating that the CH_3_COCH_3_ formation from CO_2_ reduction became more favorable under the electroreduction environment. According to the literature, a rate-limiting step for CO_2_ reduction was the activation of CO_2_ molecule to form a COOH* intermediate via the single electron transfer pathway^38–41^, which was the first step in CO_2_ reduction. In the current DFT calculation, the ΔG value calculated at -0.36 V for COOH* formation on Cu-pyrrolic-N_4_ was 1.06 eV, being lower than that obtained on Cu-pyridinic-N_4_ (1.30 eV) and indicating that CO_2_ reduction should be more facile on the Cu-pyrrolic-N_4_ site of Cu-SA/NPC. The COOH* intermediate was reduced to CO* species by reacting with a proton and releasing a H_2_O molecule. The Δ*G* values were 0.14 and −0.97 eV for CO* formation on Cu-pyrrolic-N_4_ and Cu-pyridinic-N_4_ sites, respectively. Based on the lowest energy pathways for CH_3_COCH_3_ generation, the first C–C coupling of two CO* species was crucial for the formation of C_2+_ products, and this step was found to have a Δ*G* value of 1.67 eV on Cu-pyridinic-N_4_ but was −1.23 eV exothermic on Cu-pyrrolic-N_4_, indicating a quite facile C–C coupling of CO* species catalyzed by the Cu-pyrrolic-N_4_ site. Then, the formed COCO* intermediate was reduced to COCOH* with a Δ*G* value of −0.05 eV and −0.53 eV on Cu-pyridinic-N_4_ and Cu-pyrrolic-N_4_, respectively. The formation of COCOH* species was also reported by Goddard III et al., which was considered as a key intermediate for C_2_ or C_3_ product formation from CO_2_ reduction^42^. The COCOH* was converted into the COC* species by breaking the C–O(H) bond, and the formed COC* was further reduced to the COCH_3_* intermediate via sequential hydrogenation steps. These steps were all downhill in free energies on the both sites at −0.36 V, as shown in Fig. 5a. The subsequent C–C bond formed by coupling of the COCH_3_* intermediate with another adsorbed CO* led to the formation of COCOCH_3_* with a Δ*G* value of −0.18 eV on Cu-pyridinic-N_4_ and −0.92 eV on Cu-pyrrolic-N_4_, showing a more favorable formation of C_3_ species from C_2_ intermediates on the Cu-pyrrolic-N_4_ site. Subsequent conversions of COCOCH_3_* to acetone were all downhill in free energy change on Cu-pyrrolic-N_4_ whereas the elementary step of COCOCH_3_* reduction to COHCOCH_3_* still had an endothermic Δ*G* of 0.70 eV on Cu-pyridinic-N_4_. The free energy calculations revealed that CO_2_ activation to COOH* was the only slow step on Cu-pyrrolic-N_4_ (Supplementary Table 6), determining the overall rate for CH_3_COCH_3_ production. The C–C coupling reactions were quite facile to occur, leading to a high selectivity to acetone formation on this site. Other possible C–C coupling pathways including CO*–CHO* and CO*-COH* were also considered on Cu-pyrrolic-N_4,_ but these routes were found to be energetically unfavorable as compared with the direct coupling of two CO* species (Supplementary Note 2 and Supplementary Fig. 23). In contrast, several steps proceeded slowly on Cu-pyridinic-N_4_, including CO_2_ reduction to COOH*, CO*-CO* coupling and COCOCH_3_* reduction to COHCOCH_3_* (Supplementary Table 6). The C–C coupling was non-electrochemical reaction and could not be facilitated by the applied potential, therefore, the significantly endothermic free energy change (Δ*G* of 1.67 eV) in the coupling of two CO* species hindered acetone formation from CO_2_ reduction on the Cu-pyridinic-N_4_ site. These calculation results revealed that Cu coordinated with pyrrolic N species (Cu-pyrrolic-N_4_) was active for acetone production rather than pyridinic N species.

To examine whether the uncoordinated pyrrolic N species was active toward acetone formation, DFT calculations were performed on two types of uncoordinated pyrrolic N models without Cu, as shown in Supplementary Figs. 24 and 25, and Supplementary Table 7. The free energy diagrams for acetone production form CO_2_ reduction on uncoordinated pyrrolic-N_1_, uncoordinated pyrrolic-N_4_, and Cu-pyrrolic-N_4_ at 0 V potential were compared in Supplementary Fig. 24. Clearly, the uncoordinated pyrrolic-N_4_ was not active toward acetone formation due to several uphill elementary steps other than CO_2_ reduction to COOH* (ΔG of 1.35 eV), such as COCOH* reduction to COC* (Δ*G* of 1.17 eV), COCH_2_* reduction to COCH_3_* (Δ*G* of 1.07 eV), and COCH_3_* coupling with CO* to form COCOCH_3_* (Δ*G* of 1.05 eV). For the uncoordinated pyrrolic-N_1_, although the first step of CO_2_ reduction to COOH* had a smaller Δ*G* value (0.75 eV) than that (1.42 eV) obtained on Cu-pyrrolic-N_4_, subsequent CO* formation from COOH* reduction had a larger Δ*G* of 1.24 eV. In addition, steps such as COCO* reduction to COCOH* and COHCOCH_3_* reduction to CCOCH_3_* were energetically endothermic with Δ*G* values of 0.70 and 0.97 eV, respectively, indicating that the uncoordinated pyrrolic-N_1_ did not have an advantage for acetone production. The comparison results in Supplementary Fig. 24 revealed that Cu single atom coordinated with pyrrolic N species (Cu-pyrrolic-N_4_) was catalytically more active than the uncoordinated pyrrolic N species, and should be responsible for the acetone production from CO_2_ reduction on Cu-SA/NPC. As shown in Supplementary Table 4, other than the difference in the content of pyridinic N and pyrrolic N, the content of oxidized N was also different on Cu-SA/NPC and Cu-SA/NPC_Ar_. The experimental results on the NPC catalyst without adding Cu showed no acetone formation from CO_2_ reduction, which suggested that the oxidized N should not be the active site for acetone production on Cu-SA/NPC. To further confirm this, DFT calculations of energetic pathways for CO_2_ reduction to acetone on pyridinic- and pyrrolic-N=O sites were performed (Supplementary Figs. 26–28), and detailed results were provided in the Supplementary Note 3 and Supplementary Table 8. Since several elementary steps involved in the paths were highly uphill in Δ*G* (Supplementary Fig. 26), the oxidized N sites were not active for acetone formation. Furthermore, the influence of Zn impurity on acetone production from CO_2_ reduction on the Cu-SA/NPC catalyst was also investigated by DFT calculations (Supplementary Figs. 29, 30) which revealed that Zn was not responsible for acetone production from CO_2_ reduction (Supplementary Note 4).

These DFT results revealed that the active sites for acetone synthesis from CO_2_ reduction on Cu-SA/NPC were Cu-pyrrolic-N_4_, consistent with the structural prediction of Cu/N coordination environment from EXAFS characterization and the N species identified from XPS. For CO_2_ reduction to acetone, the formation of reaction intermediates required the synergy between Cu and coordinated pyrrolic N species (Fig. 5b), leading to a facile C–C coupling toward C_2_ and C_3_ species formation (Fig. 5a). The ICP results provided in Supplementary Table 1 revealed that the content of Cu dispersed into the Cu-SA/NPC catalyst was relatively small, and the EXAFS results (Fig. 2b) confirmed that the Cu coordinated with N atom. The combined experimental and DFT studies confirmed that the Cu single atom coordinated with four pyrrolic N (Cu-pyrrolic-N_4_) should be responsible for the acetone production from CO_2_ reduction on Cu-SA/NPC, and therefore were proposed as the active sites.

**Fig. 6:**
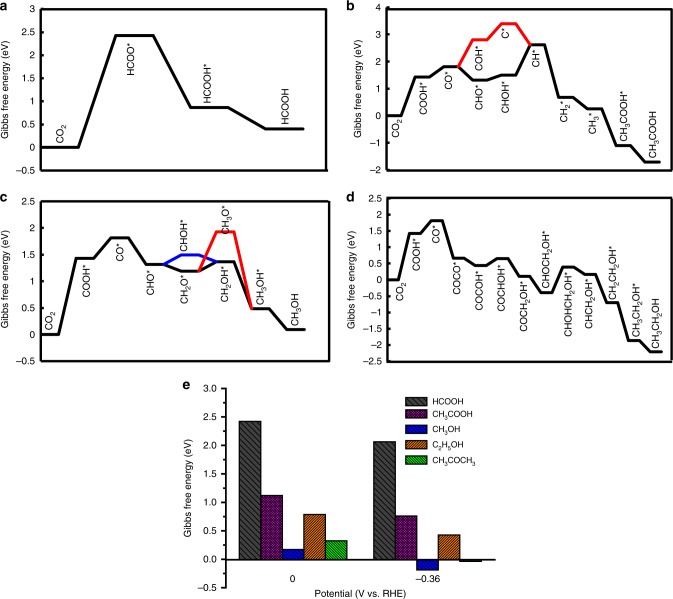
Selectivity determination by DFT calculations on Cu-SA/NPC. Free energy diagrams calculated for CO_2_ reduction to **a** HCOOH, **b** CH_3_COOH, **c** CH_3_OH and **d** C_2_H_5_OH at 0 V potential. **e** Relative selectivity evaluation based on DFT calculations on reaction free energies of all oxygenate products on the Cu-pyrrolic-N_4_ site of Cu-SA/NPC.

As observed in Fig. 3b, other oxygenates such as HCOOH, CH_3_COOH, CH_3_OH, and C_2_H_5_OH were also detected at different potentials. The energetic pathways for the formation of these oxygenates on the Cu-pyrrolic-N_4_ active site of Cu-SA/NPC were examined by DFT calculations and the results were illustrated in Fig. 6a–d. Optimized structures of all intermediates were provided in Supplementary Figs. 31–34, with the free energy change for each elementary step involved given in Supplementary Table 9. The relative selectivity to acetone versus these oxygenates was evaluated based on DFT results at 0 and −0.36 V. For acetone, the rate-determining step was CO_2_ reduction to COOH*, which had a free energy change of 1.42 eV at 0 V potential. For HCOOH formation, the rate-limiting step was CO_2_ reduction to HCOO* with a Δ*G* of 2.42 eV, determining the selectivity to formic acid and making it difficult to form at lower potentials. However, the formation of CH_3_COOH, CH_3_OH and C_2_H_5_OH all went through a CO* intermediate via CO_2_ → COOH* → CO*, similar to that for acetone formation. Therefore, the selectivity determining step for acetone versus other oxygenates should appear after CO* formation, which was found to be the COCOCH_3_* reduction to COHCOCH_3_* step with a Δ*G* of 0.32 eV for acetone formation, the CHOH* reduction to CH* + H_2_O(aq) step with a Δ*G* of 1.12 eV associated with CH_3_COOH formation, the CH_2_O* reduction to CH_2_OH* step with a Δ*G* of 0.17 eV for CH_3_OH formation, and the CHOCH_2_OH* reduction to CHOHCH_2_OH* step with a Δ*G* of 0.79 eV in regard to C_2_H_5_OH formation, at 0 V potential. Since these steps were all electrochemical reactions, the applied potential could drive these steps occurring more facilely. The relative selectivities predicted for these oxygenate products based on reaction free energy calculations at varied potentials (0 and −0.36 V) were illustrated in Fig. 6e, which was generally consistent with the experimental results shown in Fig. 3b. At lower potentials (≤−0.36 V), the HCOOH and CH_3_COOH could not be produced due to higher uphill Δ*G* values; the formation of CH_3_COCH_3_ and CH_3_OH was relatively fast because the selectivity determining step became more energetically favorable with the applied potentials; the C_2_H_5_OH could be produced but the selectivity should be lower than that of CH_3_COCH_3_ and CH_3_OH due to a moderate uphill free energy change of the selectivity determining step when the potential was applied. The higher selectivity toward acetone formation from CO_2_ reduction on the Cu-SA/NPC catalyst should originate from the unique Cu-pyrrolic-N_4_ active site in stabilizing the reaction intermediates involved in acetone production as well as facilitating the C–C coupling reactions involved in CO_2_ reduction to acetone. As shown in Fig. 5a, the two C–C coupling steps were largely downhill in free energy change (−1.22 and −0.92 eV, respectively, for CO*–CO* and CO*–COCH_3_* coupling) and the formation of reaction intermediates was energetically quite favorable (except for the first CO_2_ reduction to COOH* step) due to the synergy of Cu–N coordination and interaction with intermediate species for acetone formation on the Cu-pyrrolic-N_4_ site of Cu-SA/NPC.

## Discussion

Efficient and selective electrochemical reduction of CO_2_ to CH_3_COCH_3_ was achieved on Cu-SA/NPC. The maximum production rate of CH_3_COCH_3_ was 336.1 μg h^−1^ and the highest Faradaic efficiency of CH_3_COCH_3_ production was 36.7%, significantly enhanced as compared with other electrocatalysts reported in the literature. Based on CO_2_ reduction experiments and DFT calculations, the high activity and selectivity of Cu-SA/NPC for CH_3_COCH_3_ generation were mainly originated from single atom Cu coordinated to four pyrrolic N atoms, which lowered the reaction energies required for CO_2_ activation and C–C bond coupling. The proposed energetically most favorable pathways for CH_3_COCH_3_ generation from CO_2_ reduction went through CO_2_ → COOH* → CO* → COCO* → COCOH* →  COC* → COCH* → COCH_2_* → COCH_3_*  →  COCOCH_3_* → COHCOCH_3_* → CCOCH_3_* → CHCOCH_3_* → CH_2_COCH_3_* → CH_3_COCH_3_. The higher selectivity toward acetone formation from CO_2_ reduction on the Cu-SA/NPC catalyst should originate from the unique Cu-pyrrolic-N_4_ active sites in stabilizing the reaction intermediates involved in acetone production as well as facilitating the C–C coupling reactions due to the Cu–N synergy. This work offers fundamental insight into the design of efficient electrocatalysts for reducing CO_2_ to multi-carbon products, which is valuable in the field of energy regeneration and electrochemical synthesis.

## Methods

### Synthesis

The catalyst was obtained by carbonization of Cu-doped ZIF-8 (Cu-ZIF-8) precursor. For synthesis of Cu-ZIF-8, 5.256 g of 2-methylimidazole was dissolved in 80 mL of methanol (solution A). 4.76 g of Zn(NO_3_)_2_·6H_2_O,. and 0.31 g of Cu(CH_3_COO)_2_·H_2_O, were dissolved in 120 mL of methanol (solution B). After ultrasound for 10 min, solution B was added into solution A and the mixed solution was stirred for 30 min at room temperature. Subsequently, the mixture was transferred into Teflon-lined autoclave and heated at 120 °C for 4 h. The products were washed with methanol and DMF several times, and dried at 80 °C under vacuum. The obtained Cu-ZIF-8 powder was carbonized at 1000 °C for 4 h under nitrogen (N_2_) or argon (Ar) atmosphere. The heating rate was set to 5 °C min^−1^. The sample was denoted as Cu-SA/NPC and Cu-SA/NPC_Ar_, respectively. The catalysts with different content of Cu (0.16, 0.62, 0.93, and 1.86 g Cu with Zn/Cu ratios of 20/1, 5/1, 10/3, and 5/3, respectively) were also synthesized and the obtained catalysts were denoted as Cu-SA/NPC_0.5_, Cu-SA/NPC_2_, Cu-SA/NPC_3_, and Cu-SA/NPC_6_, respectively.

### Characterization

X-ray diffraction (XRD) patterns were measured by a Shimadzu LabX XRD-6000 diffractometer with Cu kα radiation (*λ* = 0.15406 nm). Scanning electron microscopy (SEM) and transmission electron microscopy (TEM) analyses were performed on a Hitachi S-4800 microscope and an FEI-Tecnai G^2^ 20 microscope, respectively. XPS measurements were conducted by using a VG ESCALAB 250 instrument with a monochromatized Al X-ray source (1486.6 eV). Nitrogen adsorption–desorption isotherms were obtained from a Quadrasorb instrument at 77 K. ICP-AES was detected by a Perinlmer Optima 2000DV instrument. The HAADF-STEM images were obtained on a JEOL ARM200CF fifth order aberration-corrected TEM equipped with a dual-type EDS detector. The X-ray absorption find structure spectra were measured at the BL8C beamline in Pohang Light Source (PLS), Korea.

### Electrochemical measurements

Electrochemical measurements were conducted in an airtight double-cell with a 750E electrochemical workstation. The electrolyte of cathode and anode compartments was the same and these two compartments were separated by Nafion N117 membrane. A platinum sheet and saturated calomel electrode (SCE) were served as counter electrode and reference electrode, respectively. Prior to electrochemical reduction of CO_2_, the cathode compartment was bubbled with CO_2_ for 30 min and kept being purged with CO_2_ during electrocatalytic reduction. The cyclic voltammograms tests were performed on Ar- or CO_2_-saturated electrolyte with a scan rate of 50 mV s^−1^. All the potentials were converted to the RHE using equation as: *E*_(vs. RHE)_ = *E*_(vs. SCE)_ + 0.0591 × pH + 0.241 V.

### DFT calculations

All calculations were conducted by using the Vienna Ab-initio Simulation Package^43,44^. The exchange-correlation energies were treated with the spin-polarized generalized gradient approximation and Perdew–Bruke–Ernzerh of functional^45^. Core electrons were represented by projector augmented-wave pseudopotentials^46^. A 400 eV plane wave cutoff energy and a 2 × 2 × 1 *k*-point sampling were used for all calculations. The Cu single atom doped in graphite sheet with coordination environments of four pyridine nitrogen atoms and four pyrrole nitrogen atoms were constructed and denoted as Cu-pyridinic-N_4_ and Cu-pyrrolic-N_4_, respectively. The computational hydrogen electrode model^47,48^ was utilized to calculate the free energies of intermediates involved in CO_2_ reduction under experimental conditions.

## Supplementary information

Supplementary Information

Peer Review File

## Data Availability

The authors declare that all data supporting the results of this study are available within the paper and its supplementary information files or from the corresponding authors upon reasonable request.

## Code availability

All codes are available upon reasonable request.
