# Supplementary material for: Selective electroreduction of CO2 to acetone by single copper atoms anchored on N-doped porous carbon
Source: Nat Commun. 2020 May 15;11:2455. doi: 10.1038/s41467-020-16381-8 (PMC7229121; doi:10.1038/s41467-020-16381-8)
Supplement: Supplementary file 1 — Supplementary Information [file 41467_2020_16381_MOESM1_ESM.pdf]

Supplementary Information

Selective electroreduction of CO_2 to acetone by single copper atoms anchored on N-doped porous carbon

Zhao et al.

Supplementary Figures

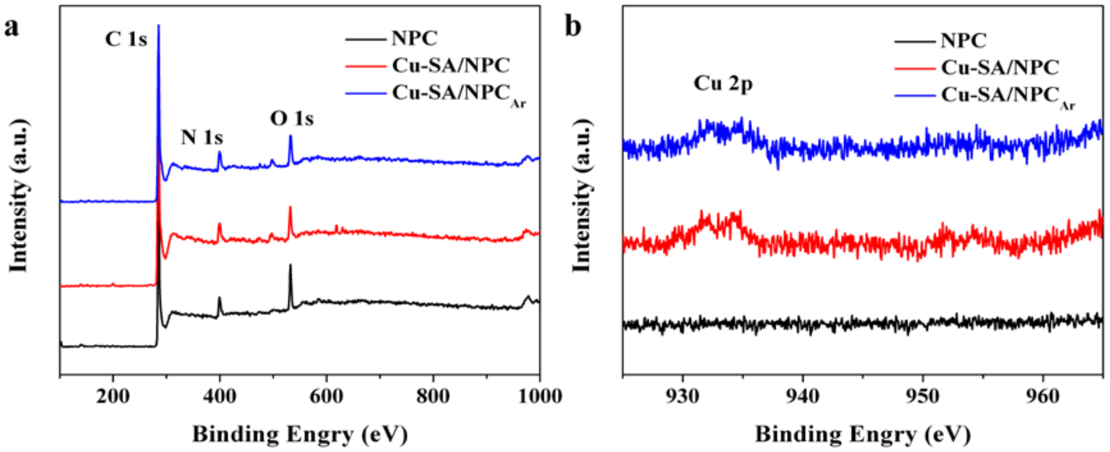

Supplementary Figure 1. XPS spectra of catalysts. **a** XPS spectra of NPC, Cu-SA/NPC and Cu-SA/NPC_{Ar}. **b** Enlarged XPS images of NPC, Cu-SA/NPC and Cu-SA/NPC_{Ar}.

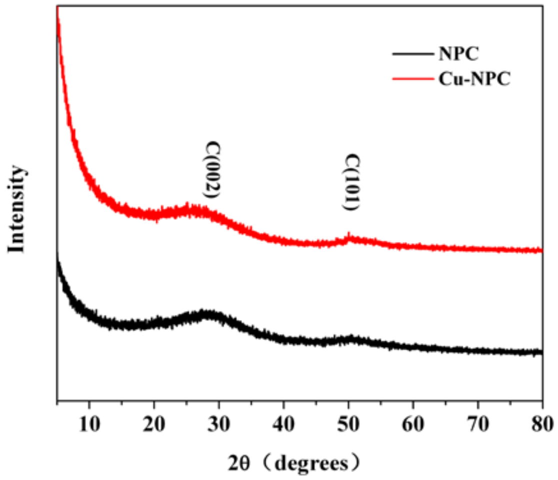

Supplementary Figure 2. XRD spectra of NPC and Cu-SA/NPC.

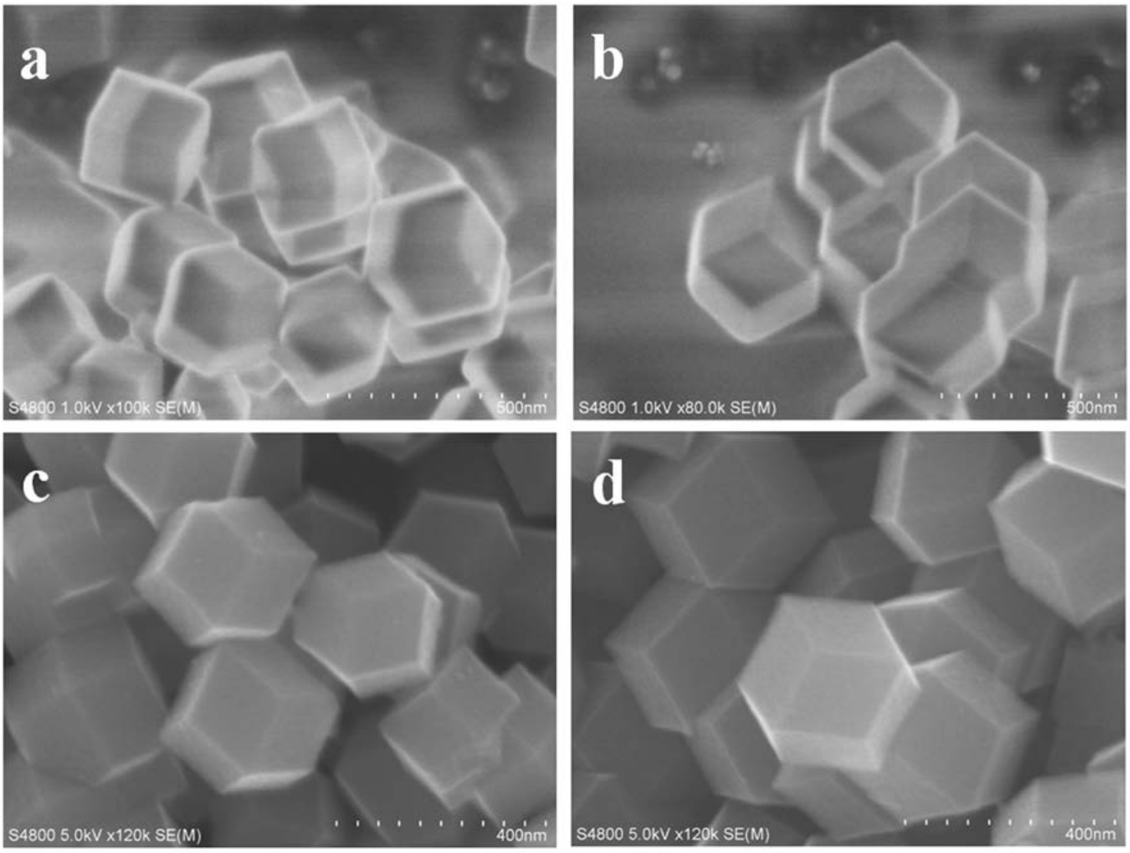

Supplementary Figure 3. SEM images of the prepared catalysts. **a** ZIF-8. **b** Cu-doped ZIF-8. **c** NPC. **d** Cu-SA/NPC.

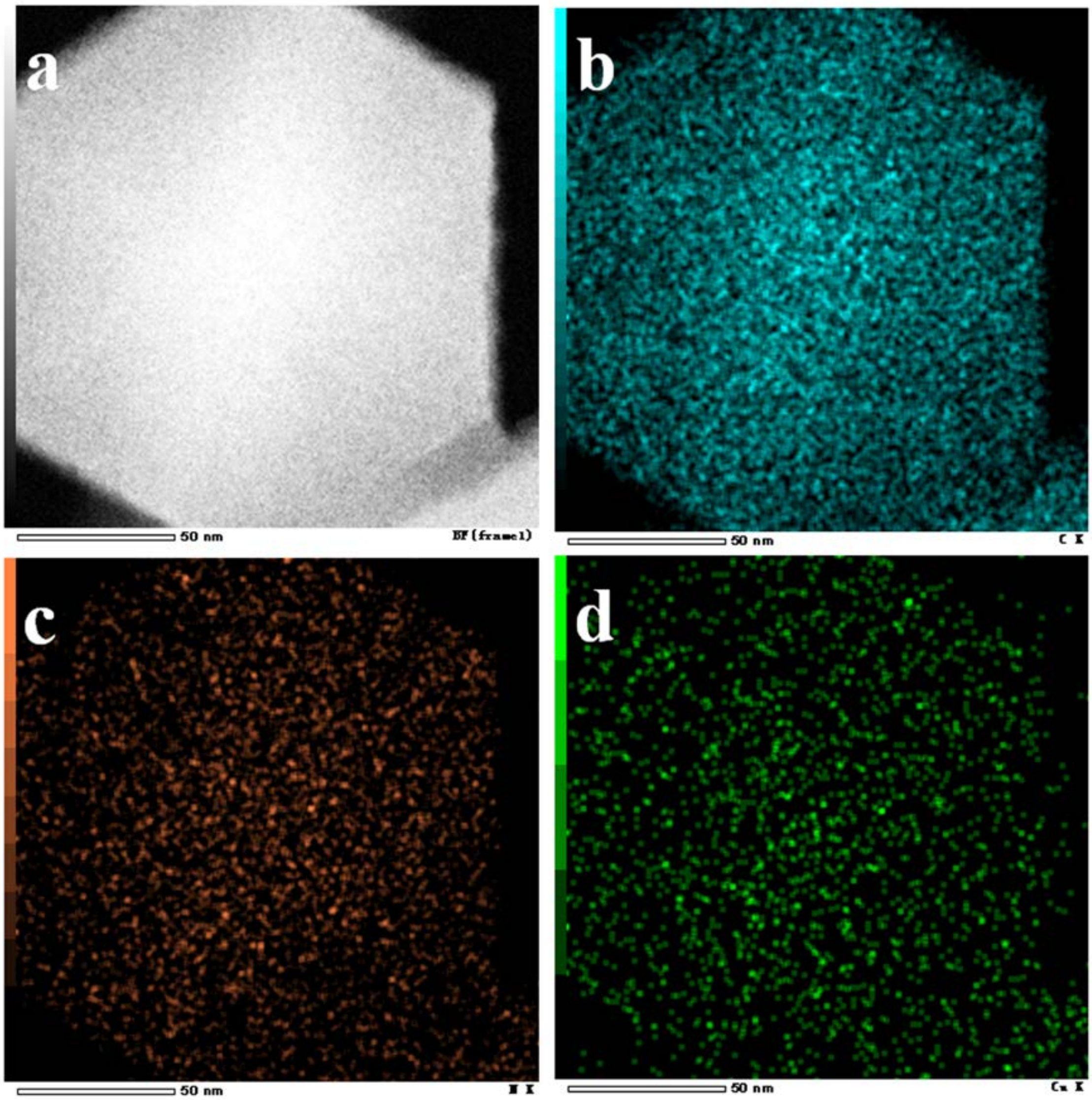

Supplementary Figure 4. EDS images of Cu-SA/NPC by HAADF-STEM. **a** STEM images. **b** C element. **c** N elemen. **d** Cu element.

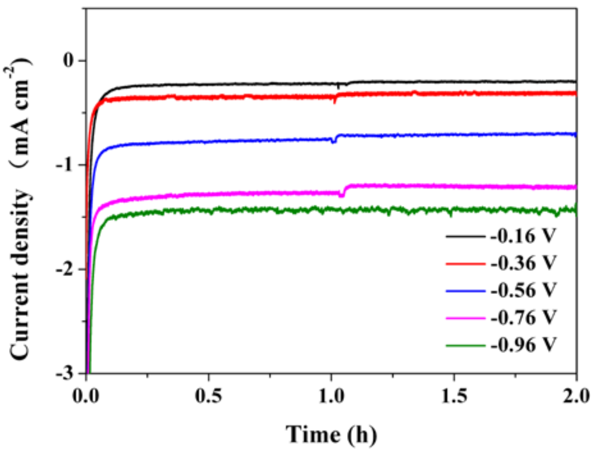

Supplementary Figure 5. Current-time curves of CO_2 electroreduction on Cu-SA/NPC at varied applied potentials in 0.1 M $KHCO_3$ solution.

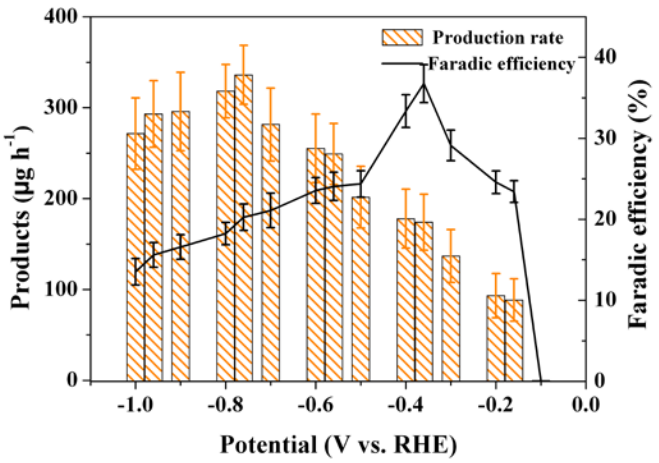

Supplementary Figure 6. Production rate and Faradic efficiency of acetone production on Cu-SA/NPC at potentials of -0.1 to -1.0 V vs. RHE.

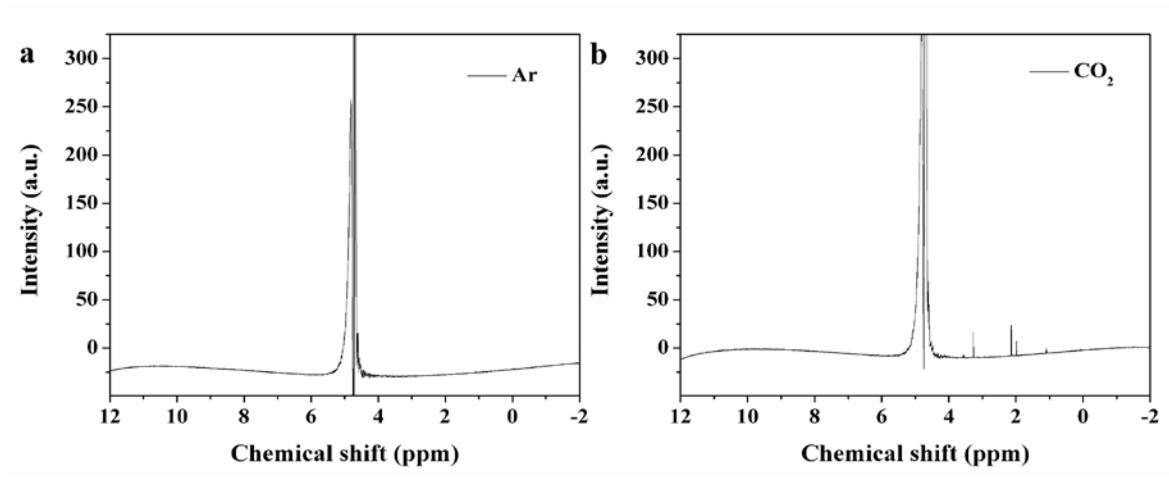

Supplementary Figure 7. ^{1}H NMR spectrum of the sample. **a** Ar-saturated electrolyte. **b** CO_2-saturated electrolyte.

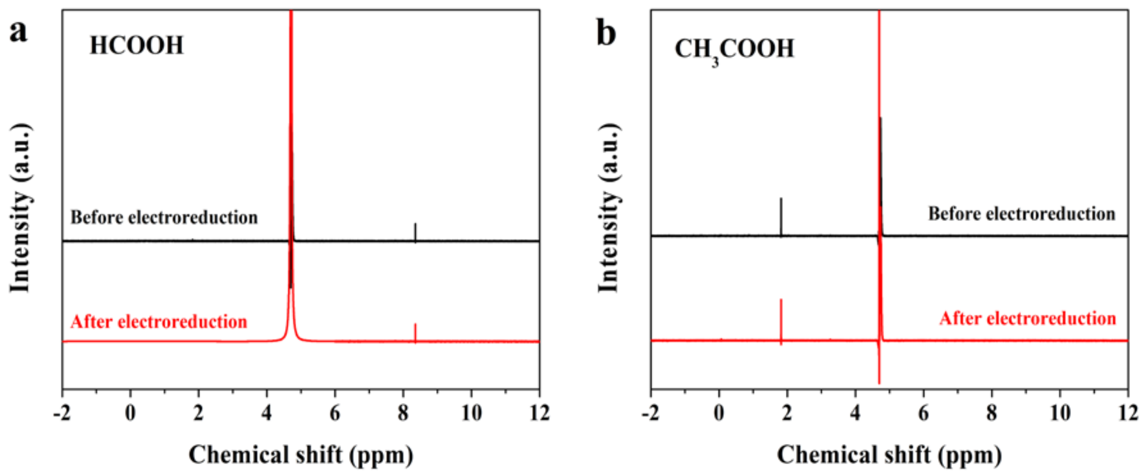

Supplementary Figure 8. ^{1}H NMR spectra of HCOOH and CH_3COOH reduction on Cu-SA/NPC catalysts. **a** HCOOH. **b** CH_3COOH.

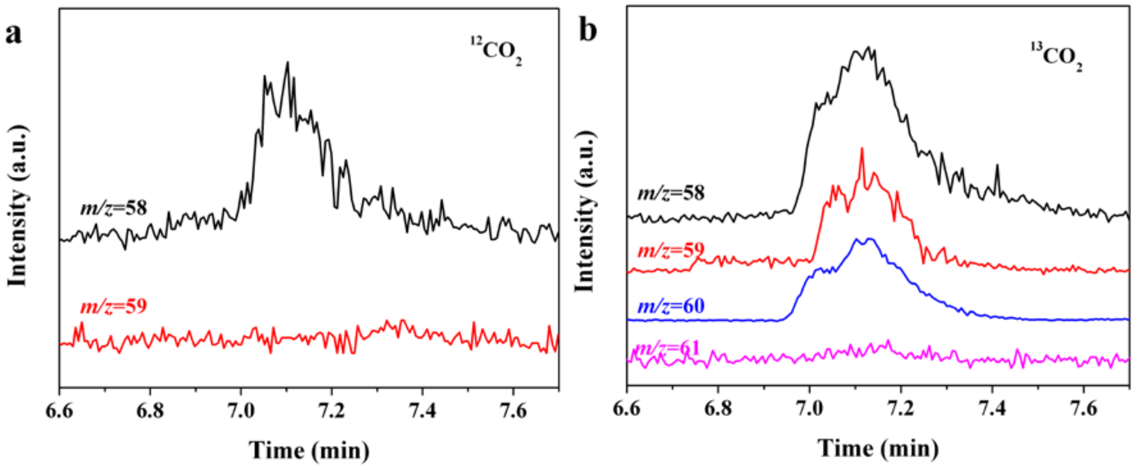

Supplementary Figure 9. GC-MS spectra of CO_2 electrochemical reduction. **a** $^{12}CO_2$. **b** $^{13}CO_2$.

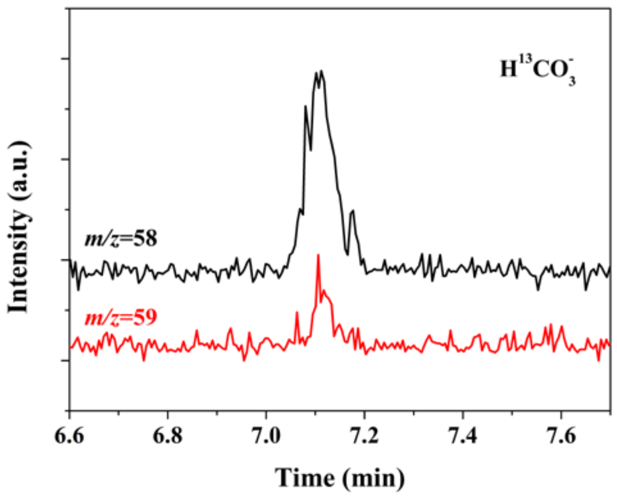

Supplementary Figure 10. GC-MS spectra of CO_2 electrochemical reduction in $H^{13}CO_3^-$.

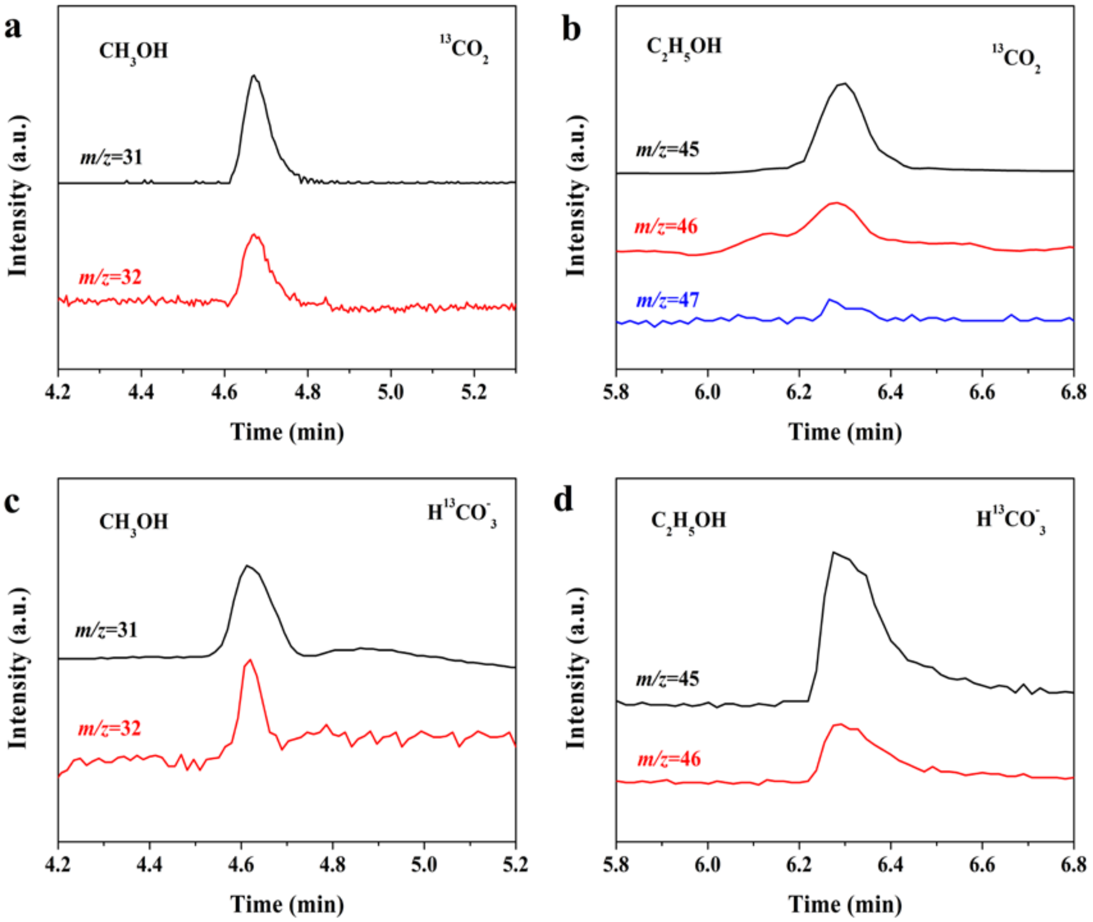

Supplementary Figure 11. GC-MS spectra. **a** CH_3OH production form $^{13}CO_2$ electrochemical reduction. **b** C_2H_5OH production form $^{13}CO_2$ electrochemical reduction. **c** CH_3OH production from CO_2 electrochemical reduction in $H^{13}CO_3^-$. **d** C_2H_5OH production from CO_2 electrochemical reduction in $H^{13}CO_3^-$.

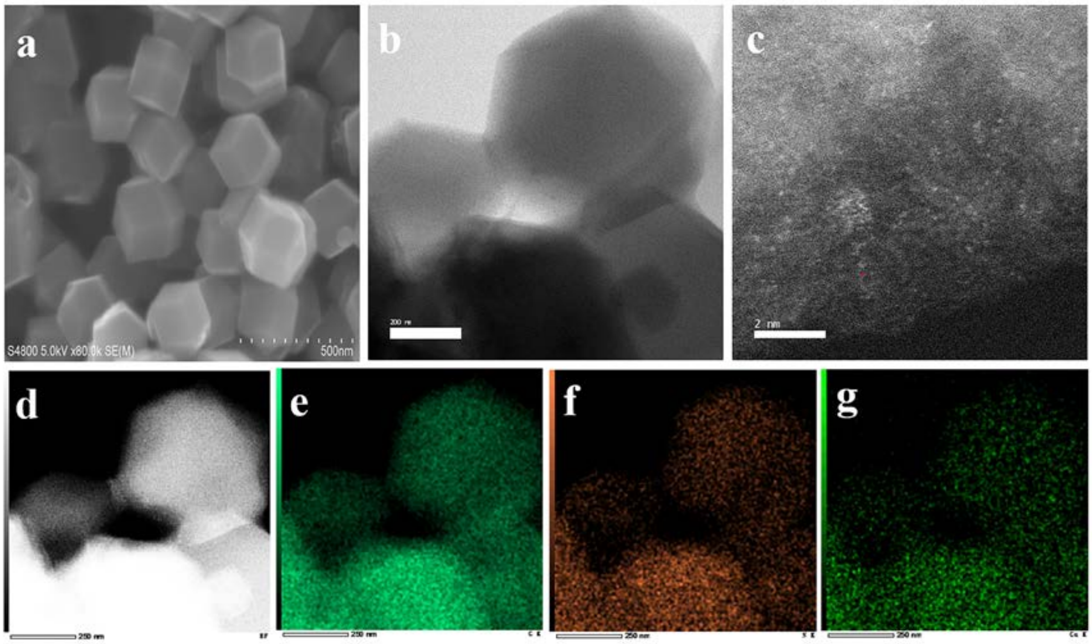

Supplementary Figure 12. Characterizations of post-catalysts. **a** SEM. **b, c** HAADF-STEM. **d-g** EDS images of post-catalysts by HAADF-STEM.

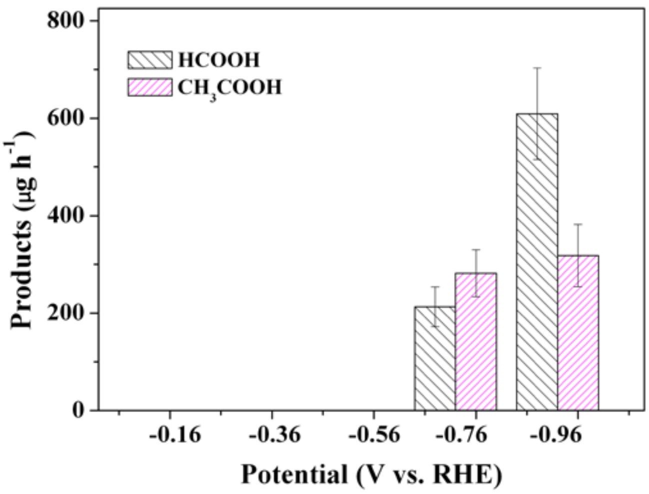

Supplementary Figure 13. Production rates of liquid products from CO_2 reduction on NPC.

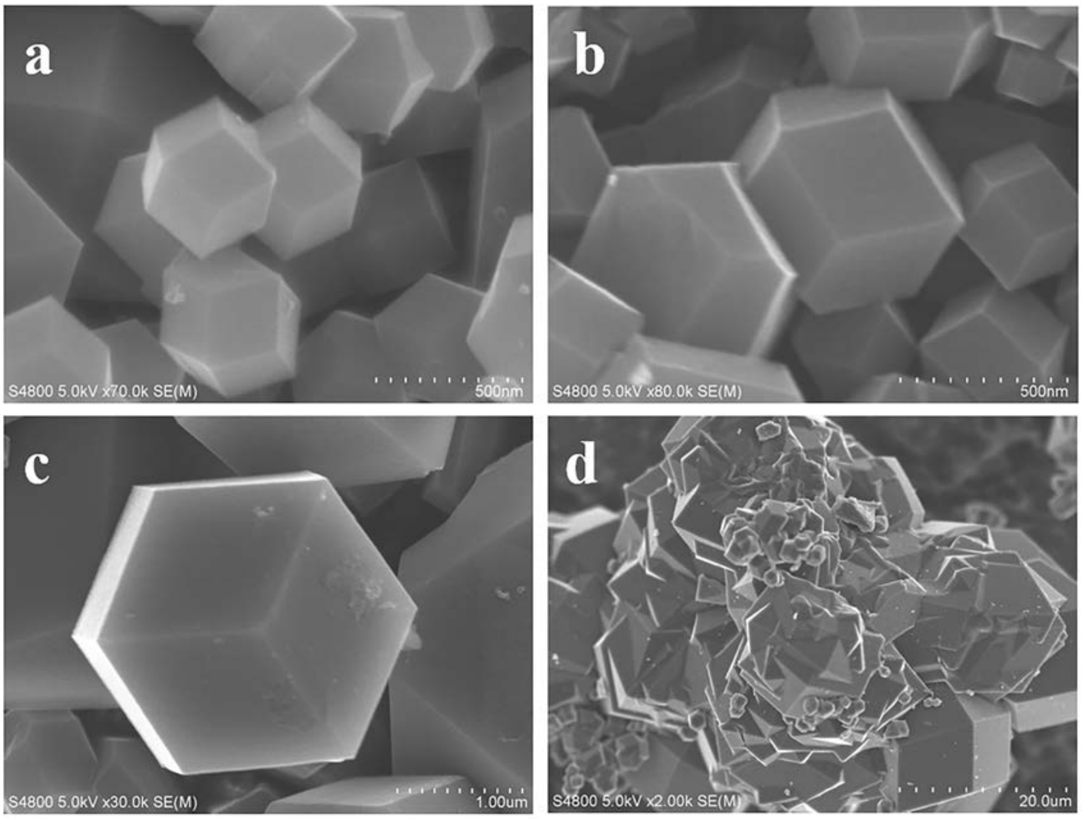

Supplementary Figure 14. SEM images of different samples. **a** Cu-SA/$NPC_{0.5}$. **b** Cu-SA/NPC_2. **c** Cu-SA/NPC_3. **d** Cu-SA/NPC_6.

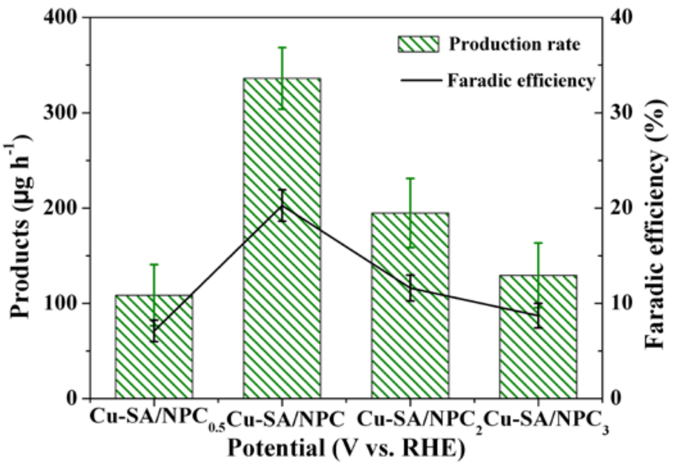

Supplementary Figure 15. Production rate and Faradic efficiency of acetone production on Cu-SA/NPC$_{0.5}$, Cu-SA/NPC, Cu-SA/NPC$_2$ and Cu-SA/NPC$_3$ at potential of -0.76 V vs. RHE.

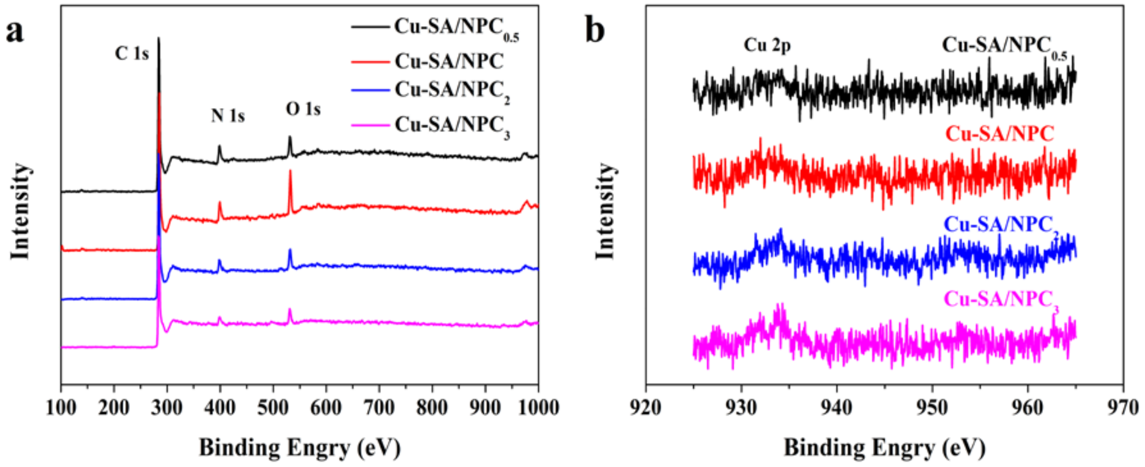

Supplementary Figure 16. XPS spectra of catalysts. **a** XPS of Cu-SA/$NPC_{0.5}$, Cu-SA/NPC, Cu-SA/NPC_2 and Cu-SA/NPC_3. **b** Enlarged XPS images of Cu-SA/$NPC_{0.5}$, Cu-SA/NPC, Cu-SA/NPC_2 and Cu-SA/NPC_3.

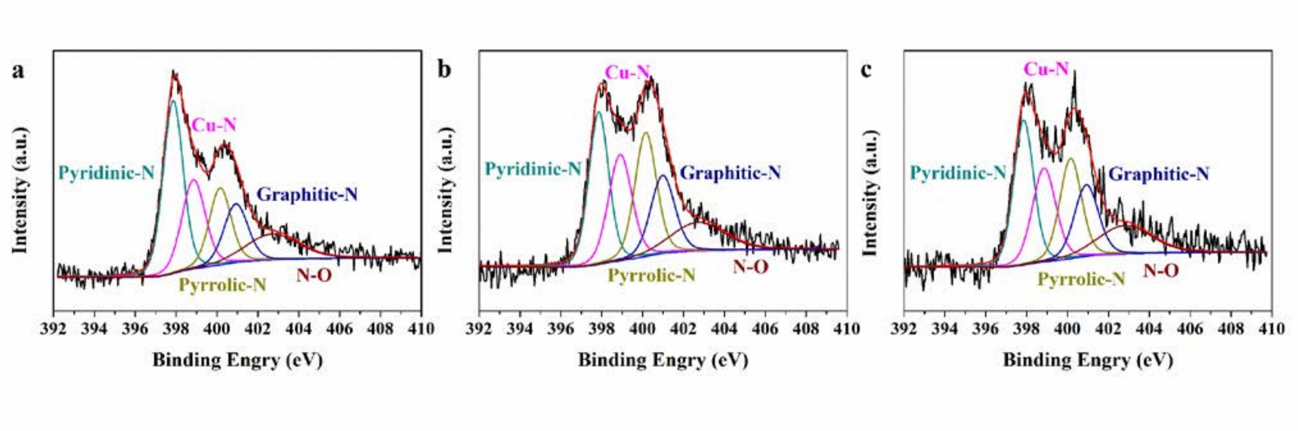

Supplementary Figure 17. XPS N 1s spectra. **a** Cu-SA/$NPC_{0.5}$. **b** Cu-SA/NPC_2. **c** Cu-SA/NPC_3.

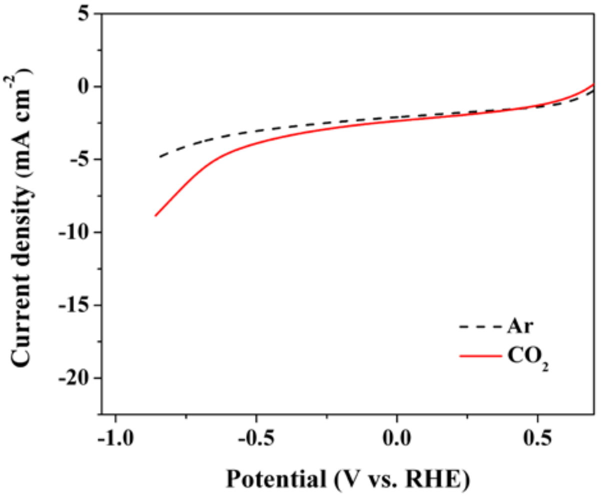

Supplementary Figure 18. LSV of Cu-SA/NPC$_{Ar}$ in Ar or CO_2 saturated solution.

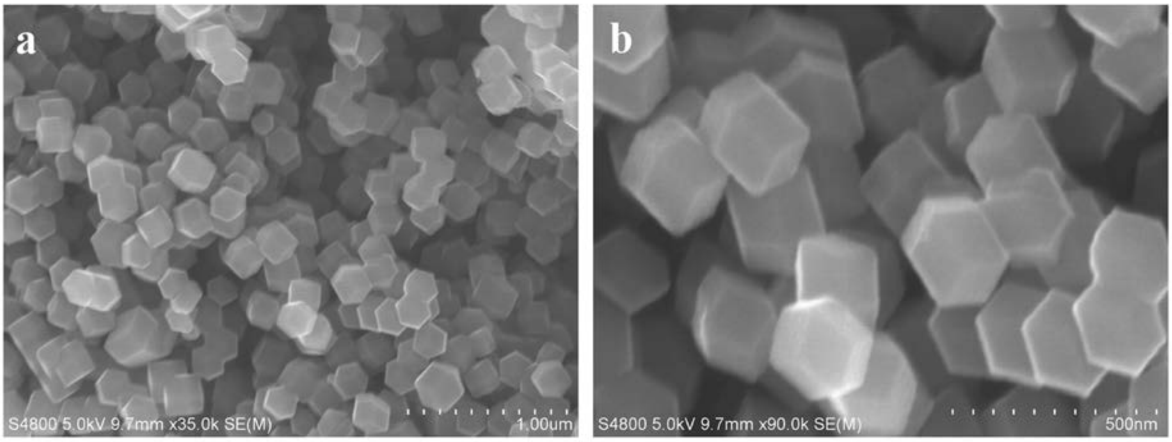

Supplementary Figure 19. Morphology characterization of Cu-SA/NPC_{Ar}. **a** SEM images of Cu-SA/NPC_{Ar}. **b** Enlarged SEM images of Cu-SA/NPC_{Ar}.

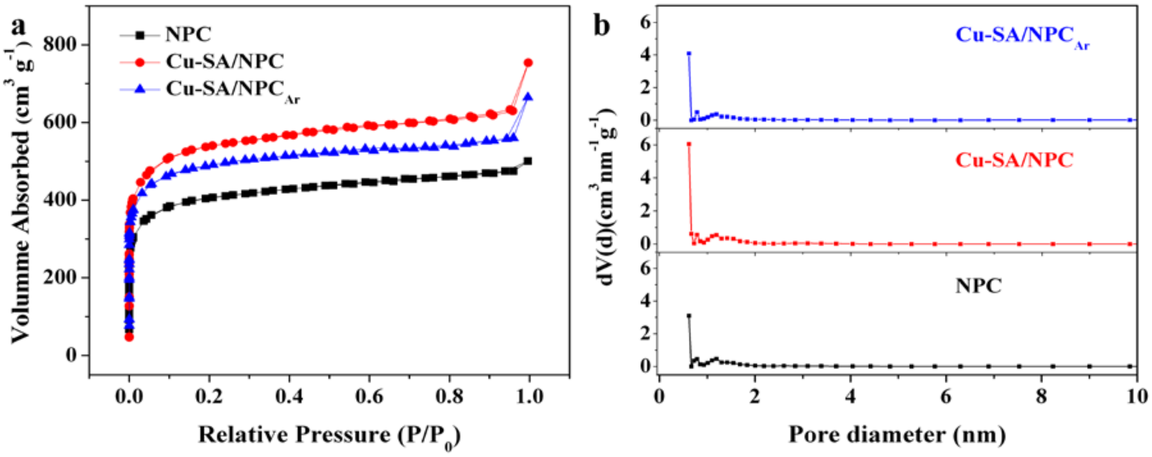

Supplementary Figure 20. N_2 adsorption-desorption characterization. **a** N_2 adsorption-desorption curves. **b** Pore distribution images of prepared catalysts.

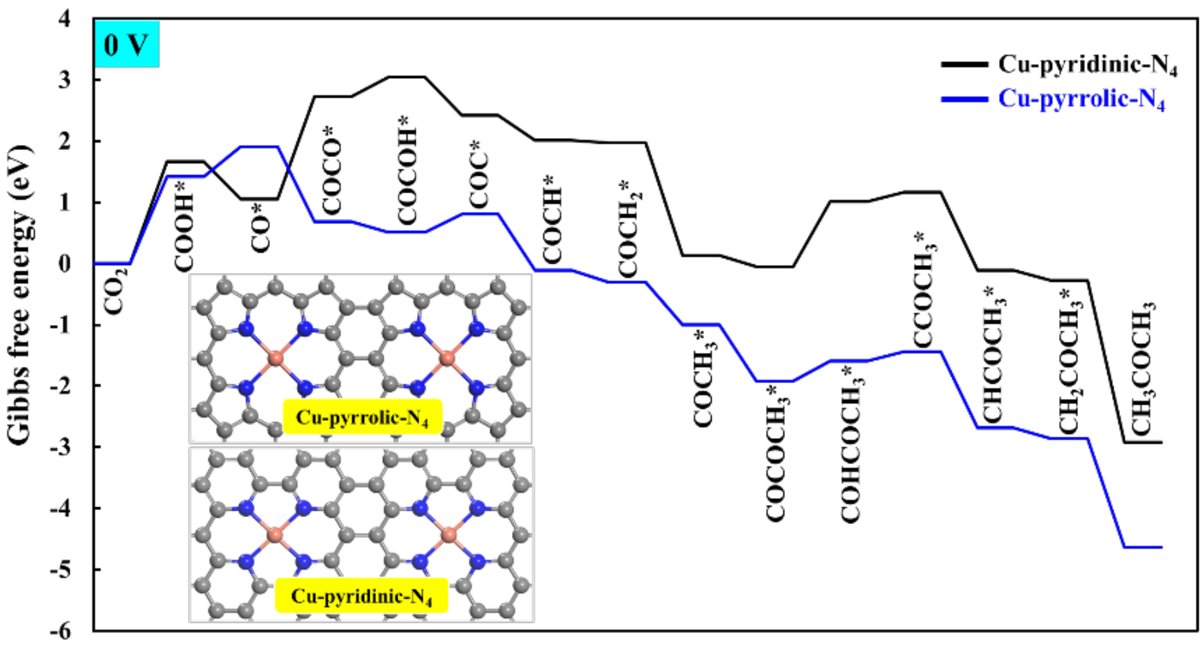

Supplementary Figure 21. Free energy diagrams calculated at 0 V for CO_2 reduction to CH_3COCH_3 on Cu-pyridinic-N_4 and Cu-pyrrolic-N_4 sites of Cu-SA/NPC. (the computational models were included in the figure) (grey: C; orange: Cu; blue: N)

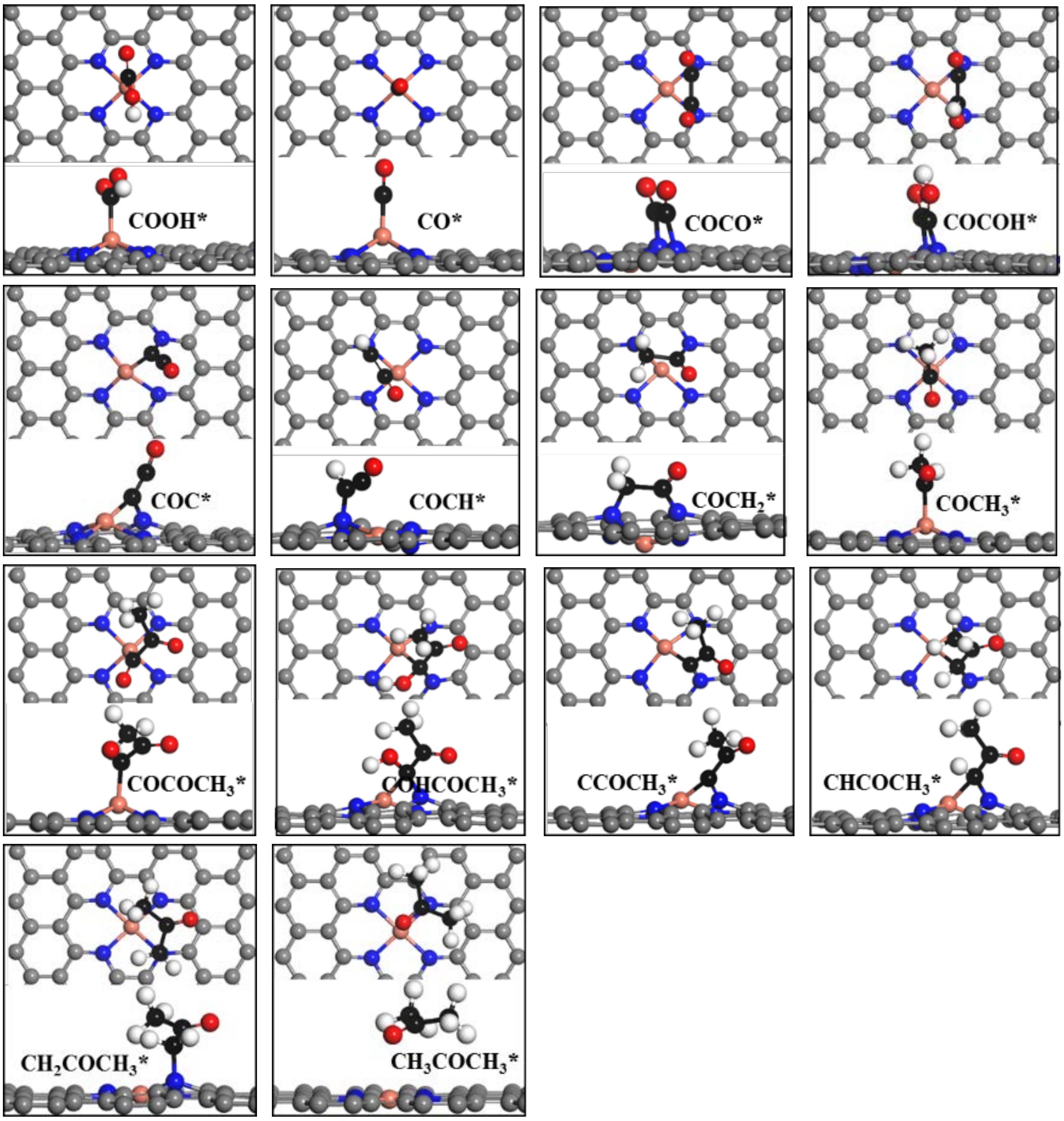

Supplementary Figure 22. Optimized structures of reaction intermediates in the pathways of CO_2 reduction to acetone on the Cu-pyridinic-N_4 site. (grey: C of catalyst; black: C of adsorbate; red: O; blue: N; orange: Cu; white: H)

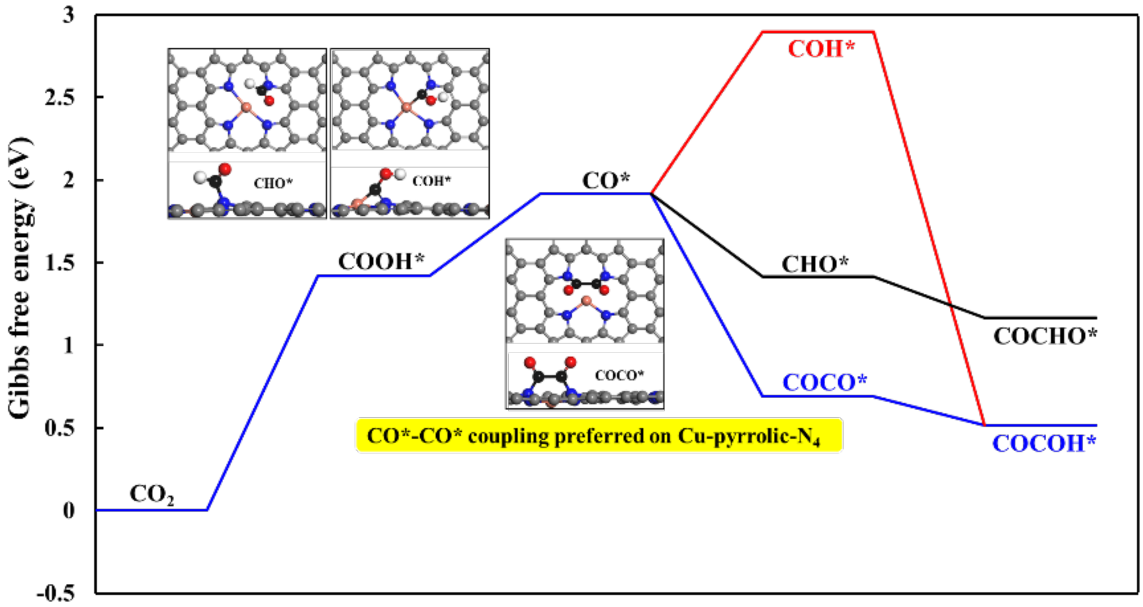

Supplementary Figure 23. Free energy diagrams calculated for CO_2 reduction through the CO*-CO* coupling, CO*-CHO* coupling, and CO*-COH* coupling pathways. The optimized structures of key CHO*, COH* and COCO* intermediates were included in the figure. (grey: C of catalyst; black: C of adsorbate; red: O; orange: Cu; blue: N; white: H)

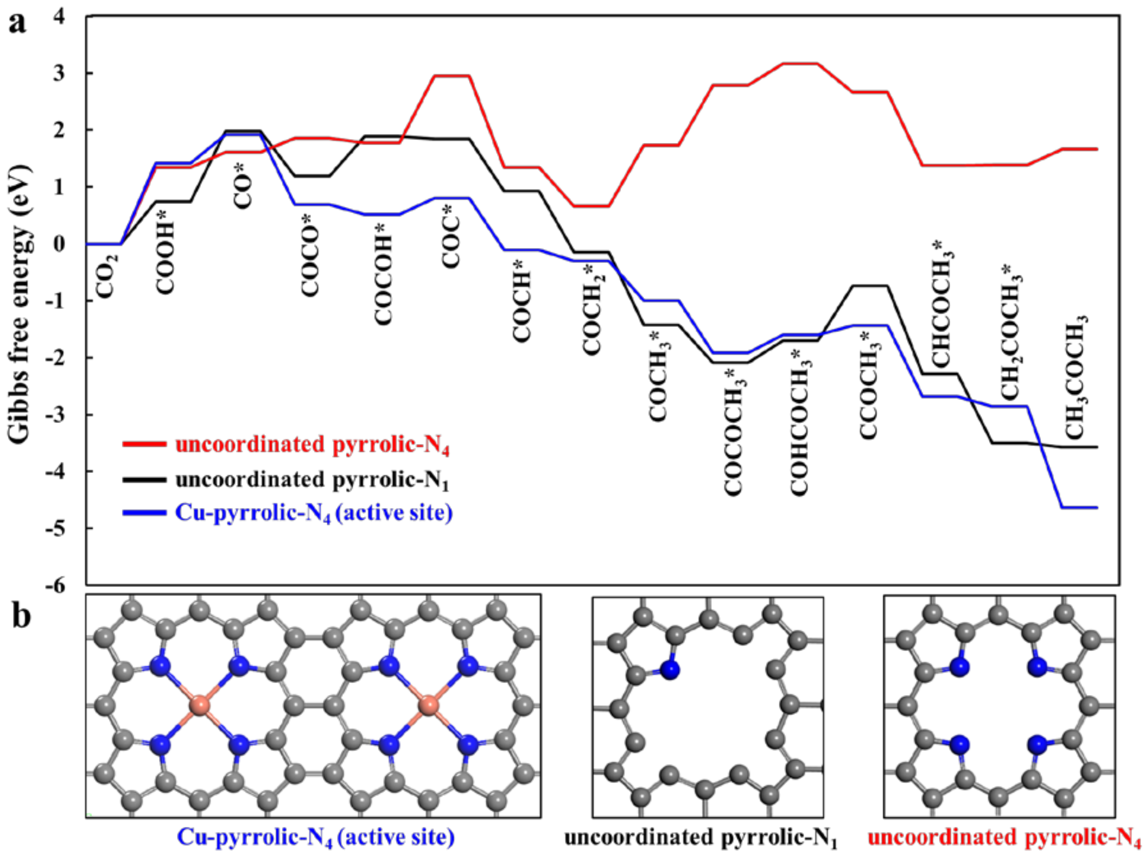

Supplementary Figure 24. DFT calculations of reaction pathways on different sites. **a** Free energy diagrams calculated at 0 eV for CO_2 reduction to CH_3COCH_3 on uncoordinated pyrrolic-N_1, uncoordinated pyrrolic-N_4 and coordinated Cu-pyrrolic-N_4 sites. **b** The computational models of these sites examined.

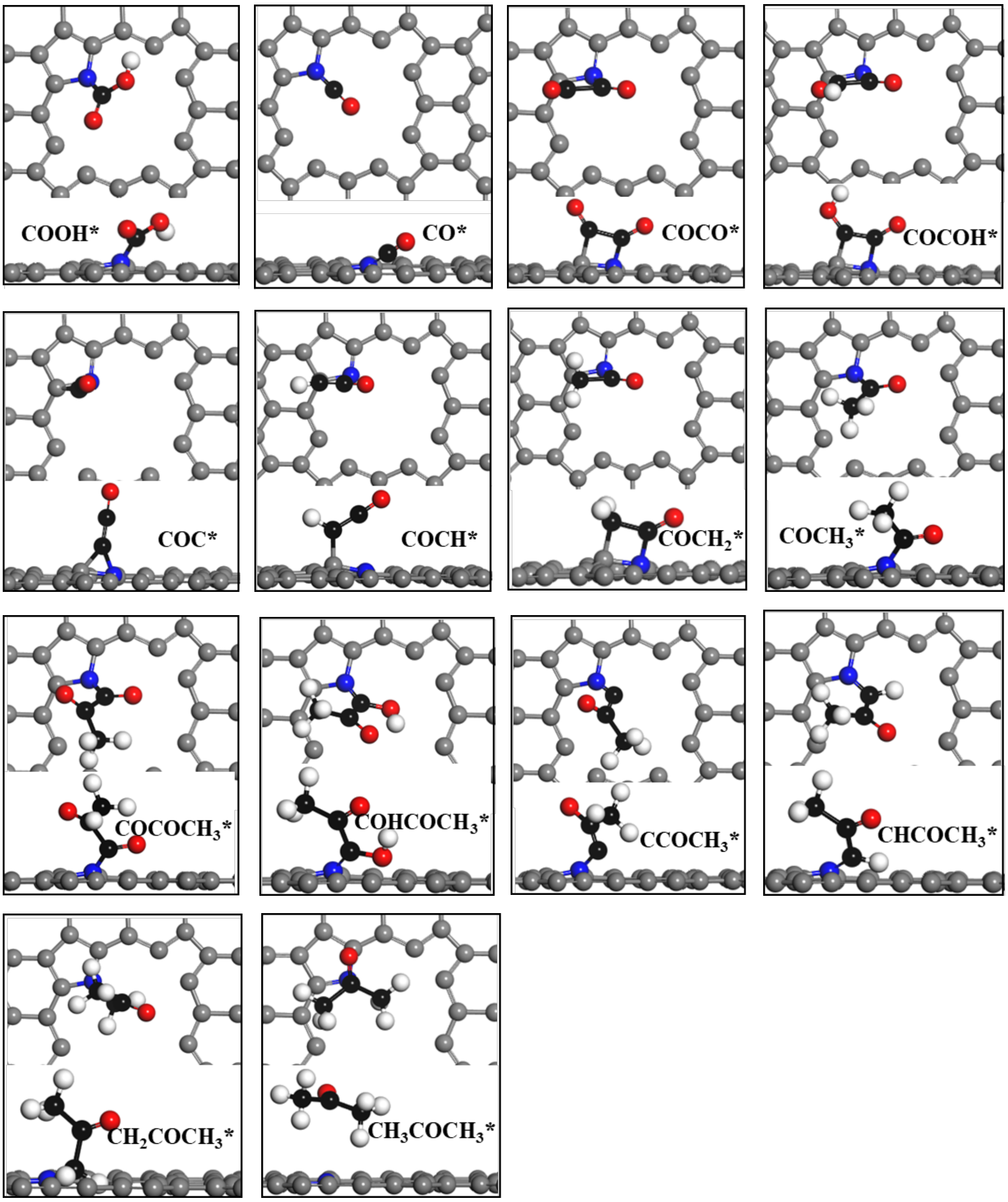
COOH*
CO*
COCO*
COCOH*
COC*
COCH*
$COCH_2$*
$COCH_3$*
$COCOCH_3$*
$COHCOCH_3$*
$CCOCH_3$*
$CHCOCH_3$*
CH_2COCH_3*
CH_3COCH_3*

a

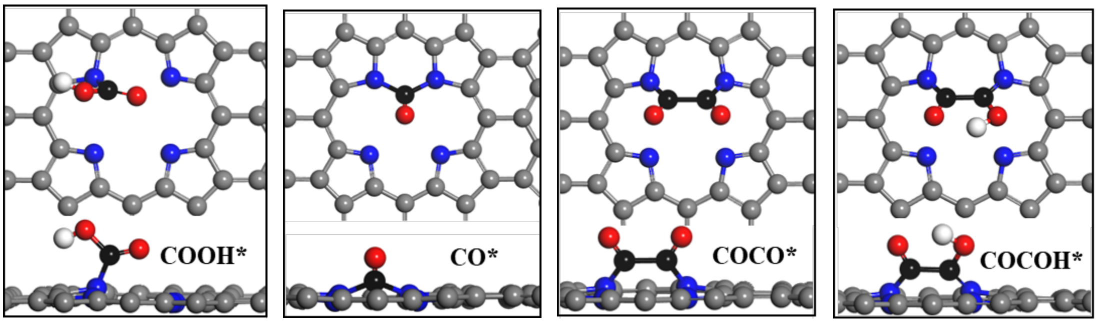
COOH*
CO*
COCO*
COCOH*

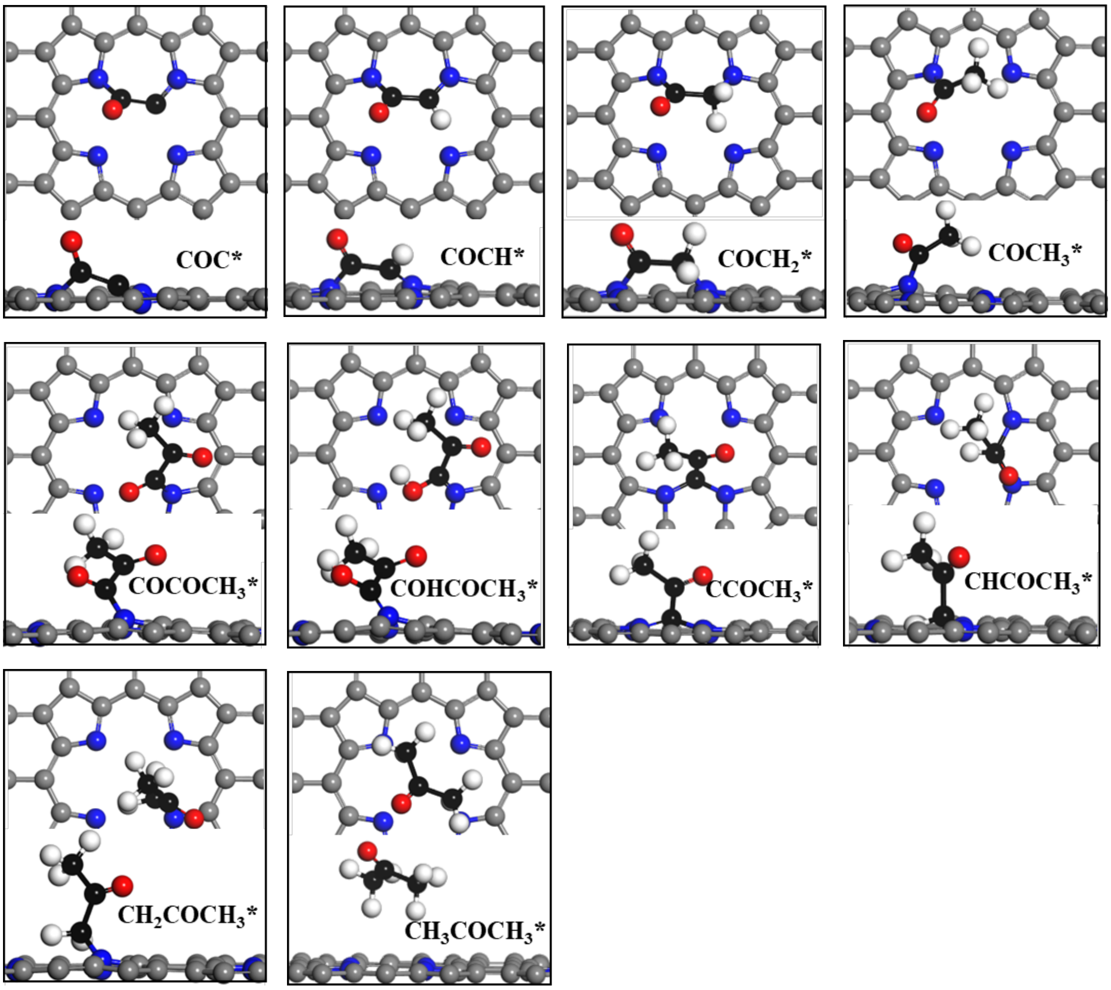

b

Supplementary Figure 25. Optimized structures of reaction intermediates in the pathways of CO_2 reduction to acetone on different sites. **a** uncoordinated pyrrolic-N_1 site. **b** uncoordinated pyrrolic-N_4 site. (grey: C of catalyst; black: C of adsorbate; red: O; blue: N; white: H)

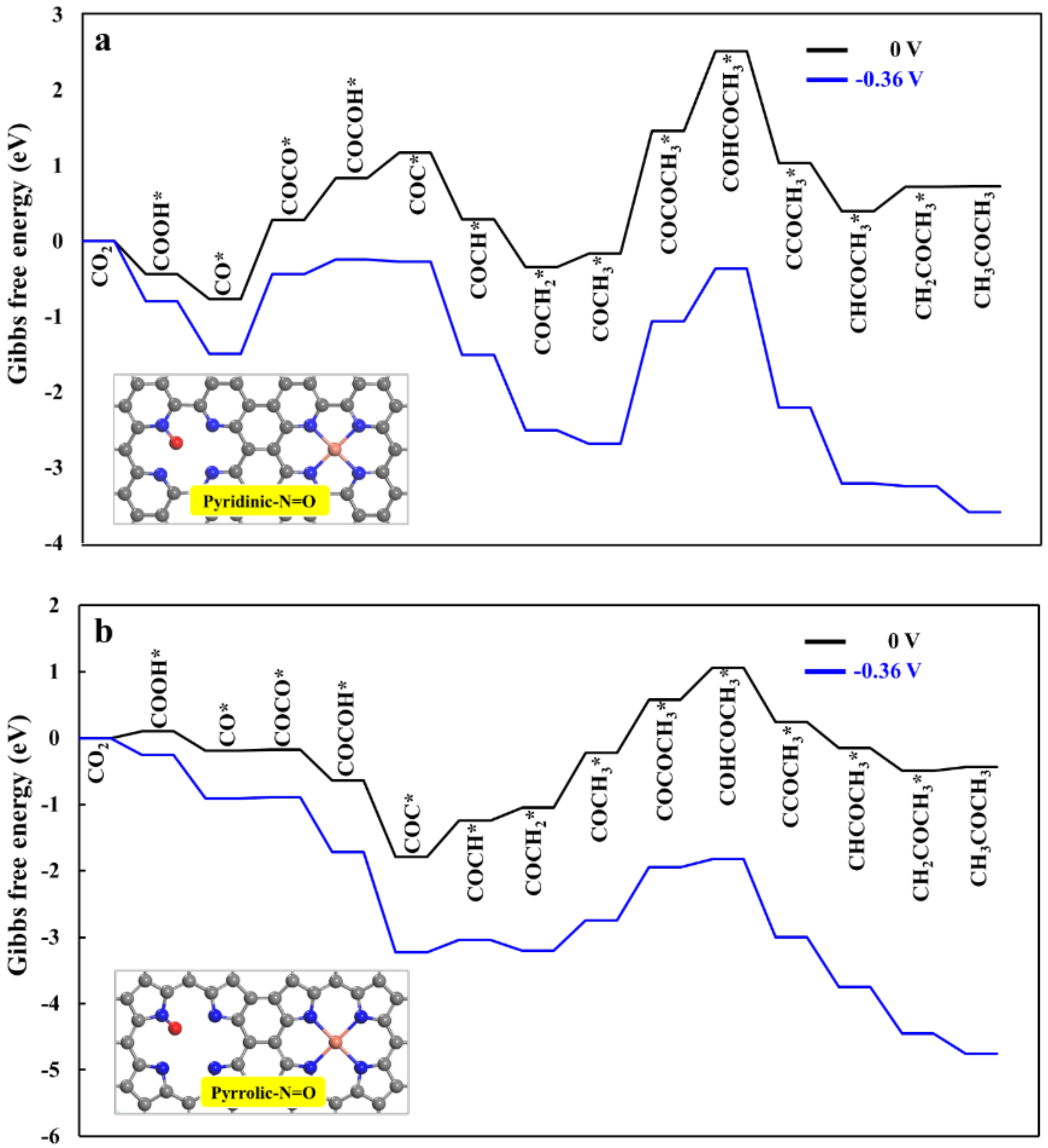

Supplementary Figure 26. Free energy diagrams calculated at potentials of 0 and -0.36 V for CO_2 reduction to CH_3COCH_3 on different sites. **a** Pyridinic-N=O site. **b** Pyrrolic-N=O site. The two computational models were included in the figures. (grey: C; red: O; blue: N; orange: Cu)

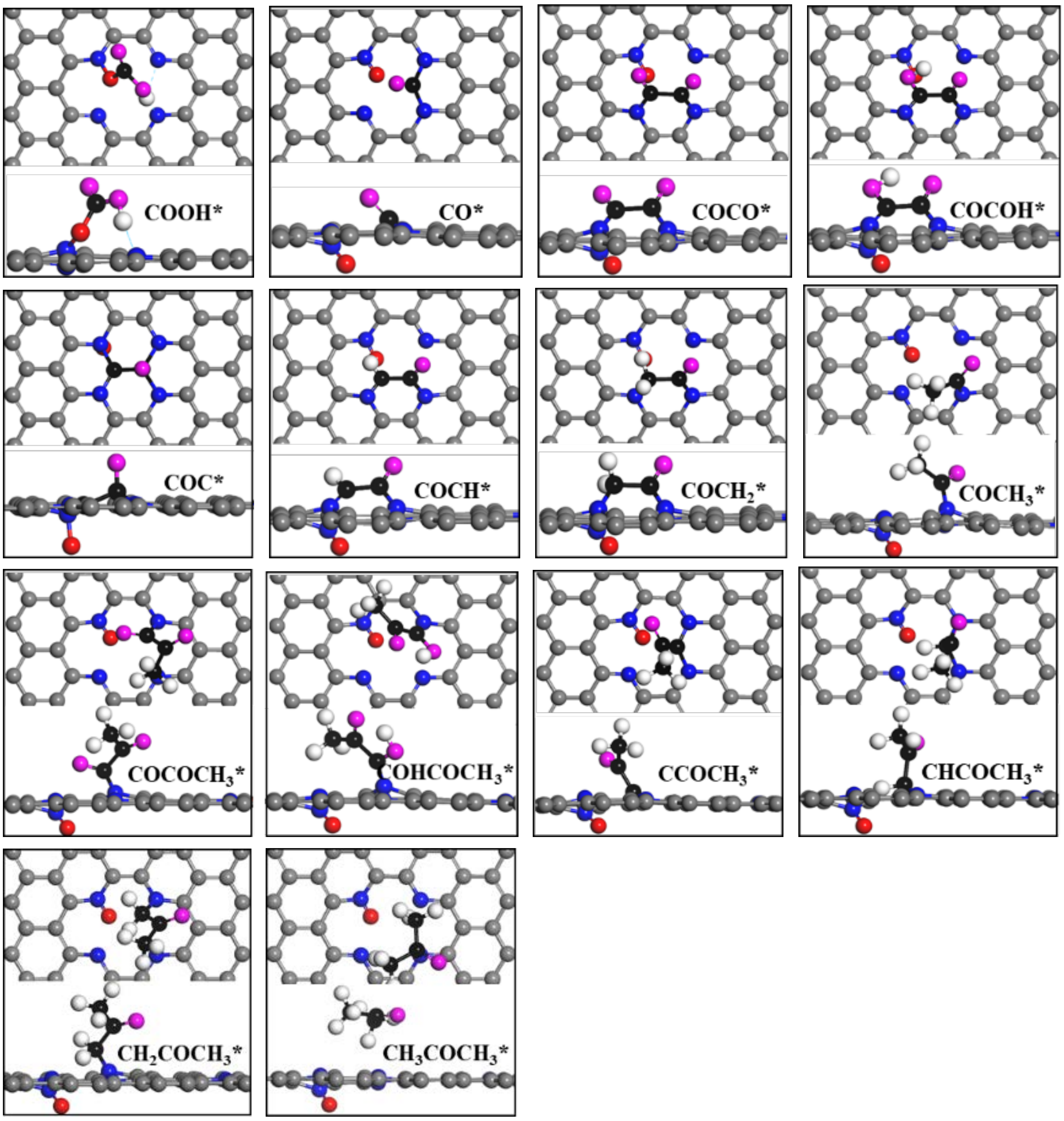

Supplementary Figure 27. Optimized structures of reaction intermediates in the pathways of CO_2 reduction to acetone on the pyridinic-N=O site. (grey: C of catalyst; black: C of adsorbate; red: O of catalyst; pink: O of adsorbate; blue: N; white: H)

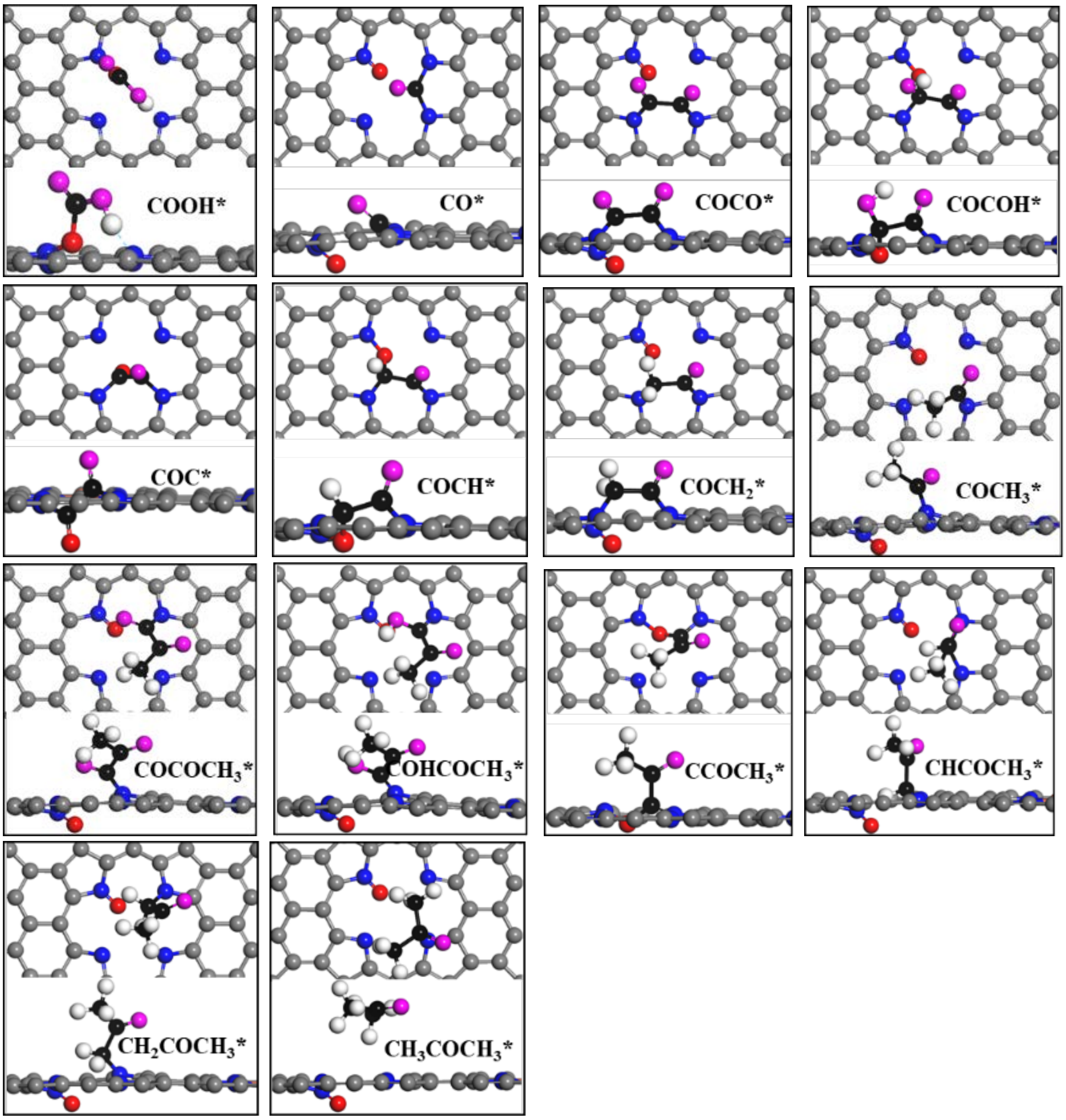

Supplementary Figure 28. Optimized structures of reaction intermediates in the pathways of CO_2 reduction to acetone on the pyrrolic-N=O site. (grey: C of catalyst; black: C of adsorbate; red: O of catalyst; pink: O of adsorbate; blue: N; white: H)

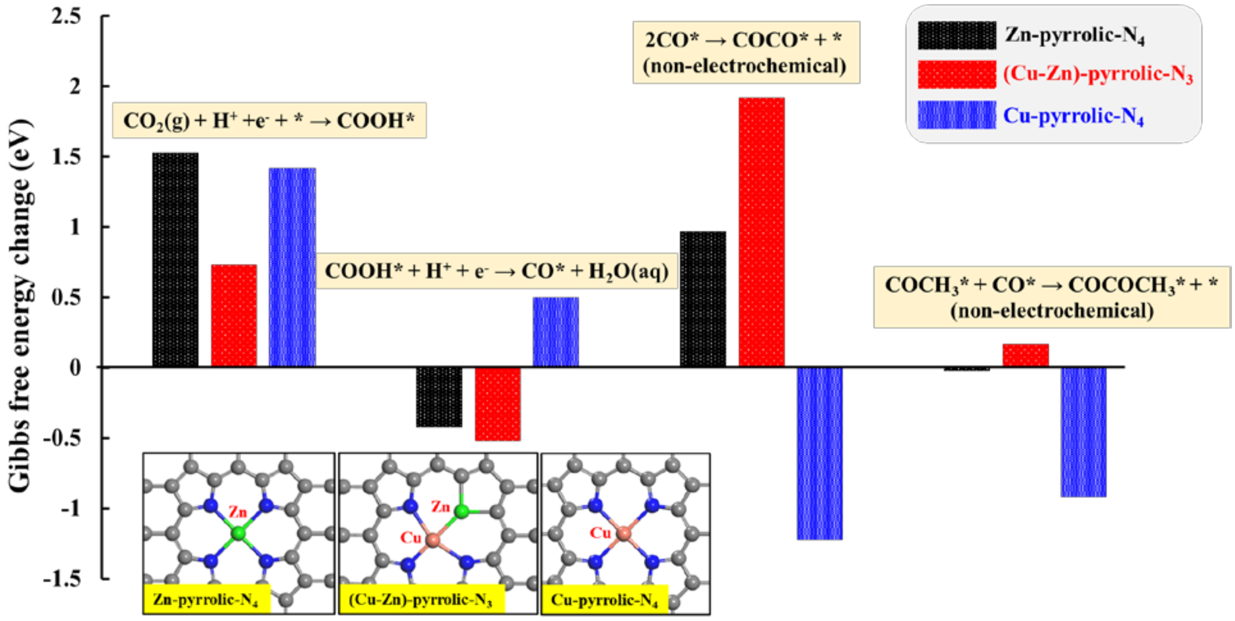

Supplementary Figure 29. Gibbs free energy change for key elementary steps involved in CO_2 reduction to acetone on different catalyst models including Cu-pyrrolic-N_4, Zn-pyrrolic-N_4 and (Cu-Zn)-pyrrolic N_3 sites.

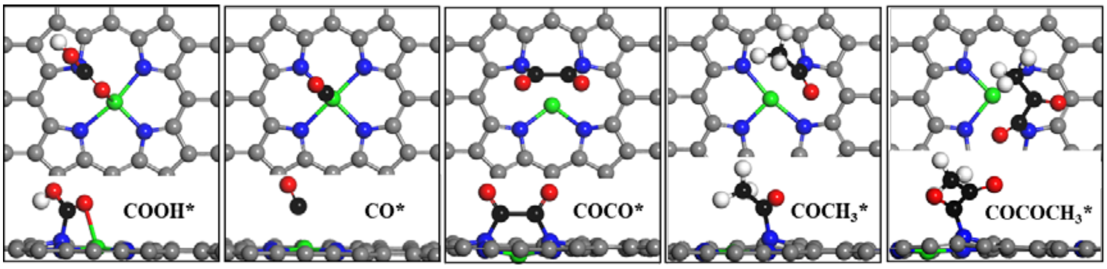

a

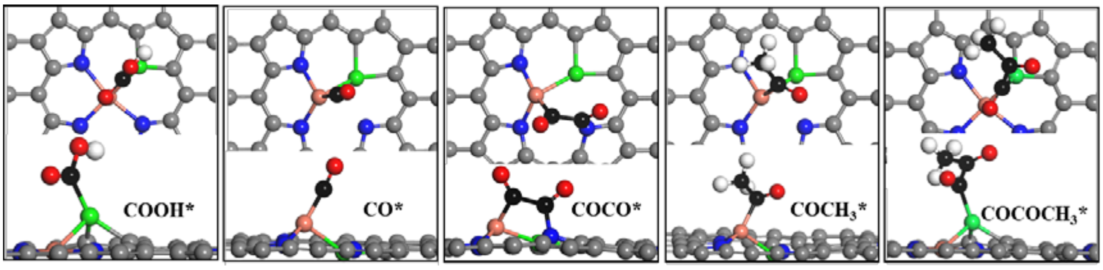

b

Supplementary Figure 30. Optimized structures of key intermediates involved in acetone formation from CO_2 reduction on different sites. **a** Zn-pyrrolic-N_4 catalyst model. **b** (Cu-Zn)-pyrrolic-N_3 catalyst model. (grey: C of catalyst; black: C of adsorbate; red: O; orange: Cu; green: Zn; blue: N; white: H)

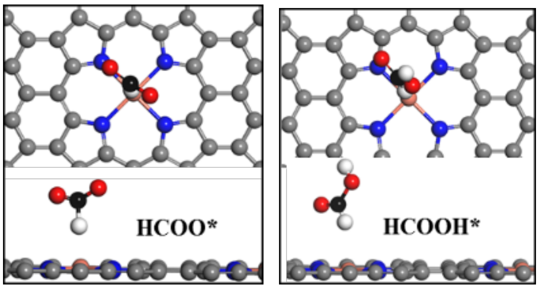

Supplementary Figure 31. Optimized structures of reaction intermediates in the pathways of CO_2 reduction to formic acid on the Cu-pyridinic-N_4 site. (grey: C of catalyst; black: C of adsorbate; red: O; orange: Cu; blue: N; white: H)

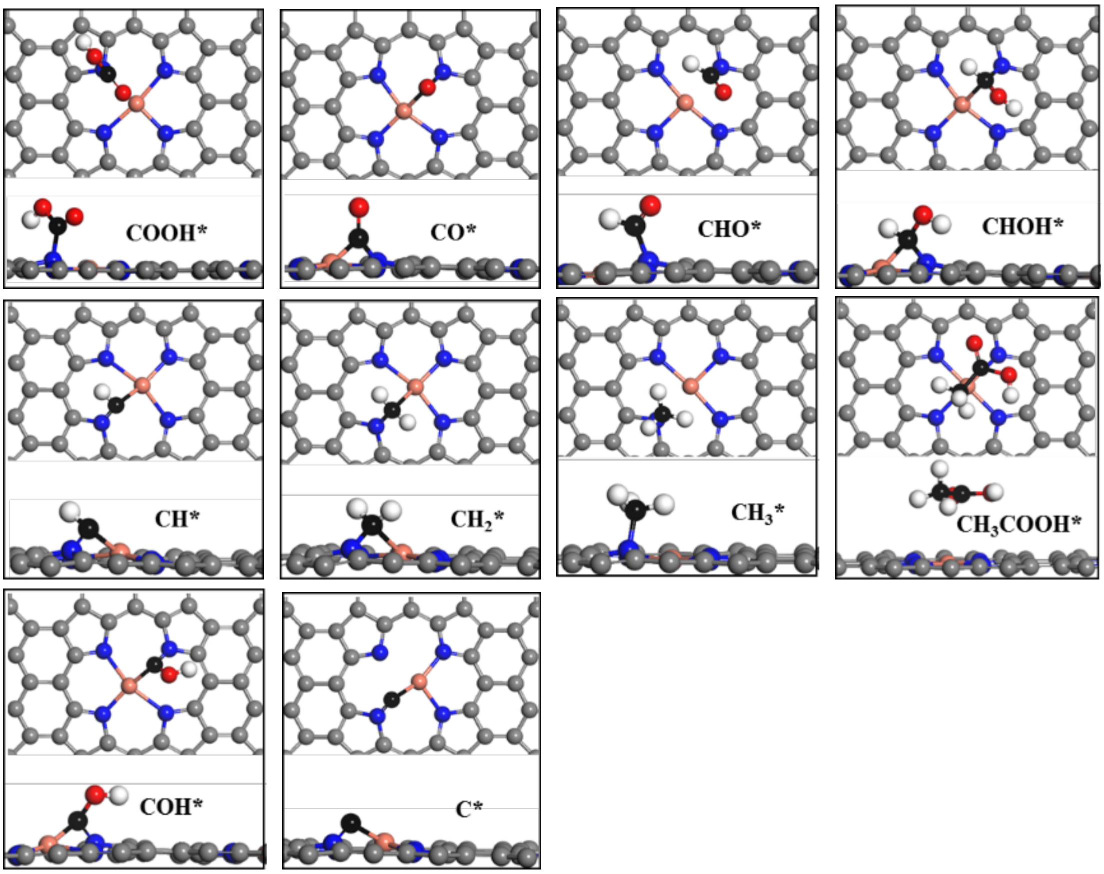

Supplementary Figure 32. Optimized structures of reaction intermediates in the pathways of CO_2 reduction to acetic acid on the Cu-pyridinic-N_4 site. (grey: C of catalyst; black: C of adsorbate; red: O; orange: Cu; blue: N; white: H)

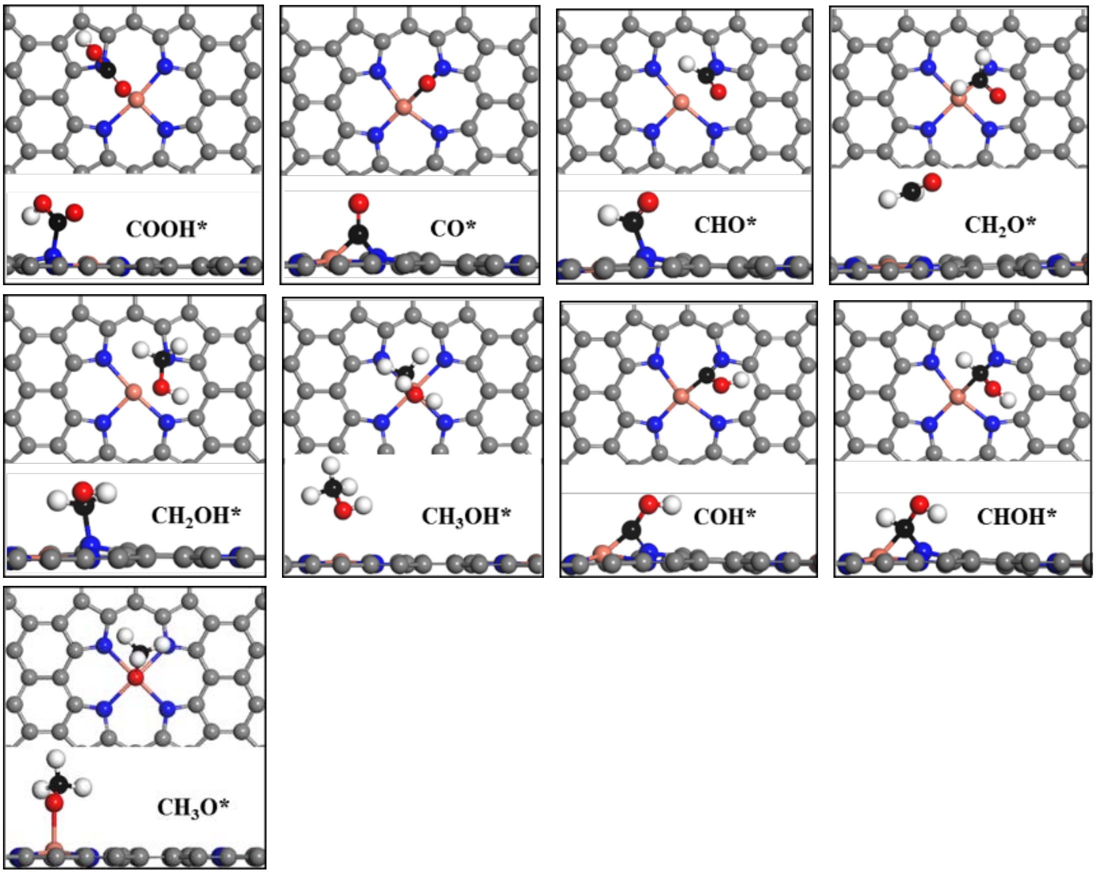

Supplementary Figure 33. Optimized structures of reaction intermediates in the pathways of CO_2 reduction to methanol on the Cu-pyridinic-N_4 site. (grey: C of catalyst; black: C of adsorbate; red: O; orange: Cu; blue: N; white: H)

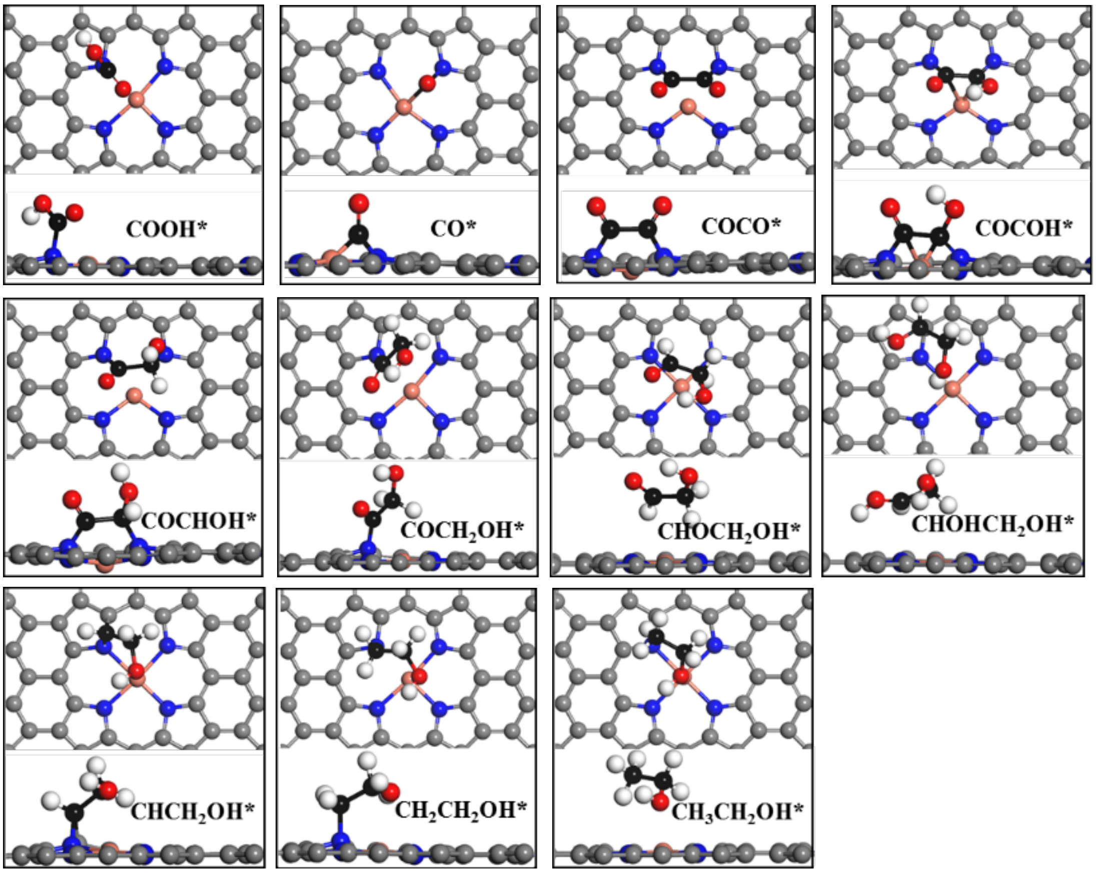

Supplementary Figure 34. Optimized structures of reaction intermediates in the pathways of CO_2 reduction ethanol on the Cu-pyridinic-N_4 site. (grey: C of catalyst; black: C of adsorbate; red: O; orange: Cu; blue: N; white: H)

Supplementary Tables

Supplementary Table 1. Inductively coupled plasma atomic emission spectrometry data for Cu and Zn contents in the prepared catalysts.

Sample	ZIF-8	NPC	Cu-doped ZIF-8	Cu-SA/NPC	Cu-SA/NPC_{Ar}
Cu (wt %)	--	--	0.12	0.59	0.60
Zn (wt %)	19.21	0.90	18.04	0.07	0.06

Supplementary Table 2. Cu K-edge EXAFS curves fitting parameters.

Sample	Path	N	R(Å)	E_0	σ^2
Cu-SA/NPC	Cu-N	3.8±0.2	1.93±0.01	-0.4±0.8	0.005±0.001

Supplementary Table 3. The comparison of acetone production from electrochemical CO_2 reduction from this work and in the literature.

Sample	Condition	Production rate	Faradaic Efficiency	Ref.
Cu-based NP/C	-1.1 V vs.RHE, 0.1 M KCl	--	~2.0%	2
GO-VB_6-4	-0.4V vs. RHE, 0.1 M $KHCO_3$	--	8.9%	3
CuO/TiO_2-5	-0.85 V vs.RHE, 0.5 M $KHCO_3$	--	4.3%	4
CuCats	-1.2 V vs.RHE, 0.1M $KHCO_3$	--	minor	5
CuCNT-Nw	200 mA, 0.5 M $KHCO_3$	0.12 μmol h^{-1}	0.3%	6
CuCNT-ImR		0.29 μmol h^{-1}	1.0%	
1-CuCNT-ImR		0.58 μg h^{-1}	4.5%	
SIM-1/Pt-CNTs	10 mA, 0.5 M $KHCO_3$	0.10 μg h^{-1}	--	7
Cu-Cd-modified RuO_X+IrO_X	-0.8 V vs. SCE, 0.5 M $KHCO_3$	69.6 μg h^{-1}	--	8
Fe10-PS	10-20 mA, 0.5 M KCl	--	0.05%	9
Pt10-PS		--	0.22%	
Metallic copper	45 mA, 0.1 M $KHCO_3$	--	0.1%	10
PMPy/MB-Pt	-0.6 V vs. SCE, 0.5 M KCl	324.8 μg L^{-1}	--	11
Modified platinum mesh electrode	-0.6 V vs. Ag/AgCl, 0.5 M KCl	--	10.2%	12
Cu(100)	-0.4 V vs RHE, phosphate buffer pH=7.9±0.1	--	minor	13
Cu-SA/NPC	-0.36V vs. RHE, 0.1 M $KHCO_3$	174.0 μg h^{-1}	36.7%	this work
	-0.76 V vs. RHE, 0.1 M $KHCO_3$	336.1 μg h^{-1}	20.3%	

Supplementary Table 4. The content of Cu and N of prepared catalysts [a].

Sample	Cu_{Total} (at.%)	N_{Total} (at.%)	Pyridinic N (at.%)	Pyrrolic N (at.%)	Graphitic N (at.%)	Cu-N (at.%)	N-O (at.%)
NPC	--	7.8	3.3	1.9	1.5	--	1.1
Cu-SA/$NPC_{0.5}$	0.2	8.3	2.8	1.4	1.3	1.7	1.1
Cu-SA/NPC	0.2	8.4	2.2	1.8	1.4	1.8	1.2
Cu-SA/NPC_2	0.3	6.5	1.7	1.5	1.1	1.4	0.8
Cu-SA/NPC_3	0.3	6.4	1.7	1.3	1.1	1.3	1.0
Cu-SA/NPC_{Ar}	0.2	8.2	2.7	1.4	1.3	1.9	0.9

[a] Catalysts with adding Cu content of 0, 0.16, 0.31, 0.62, and 0.93 g were denoted as NPC, Cu-SA/$NPC_{0.5}$, Cu-SA/NPC, Cu-SA/NPC_2, Cu-SA/NPC_3, respectively. Catalysts carbonized at Ar atmosphere was denoted as Cu-SA/NPC_{Ar}.

Supplementary Table 5. The BET surface areas, total pore volumes and average pore diameters of prepared catalysts.

Sample	BET surface area (m^2 g^{-1})	pore volume (cm^3 g^{-1})	Average pore diameter (nm)
NPC	1180.2	0.68	0.61
Cu-SA/NPC	1565.4	1.39	0.61
Cu-SA/NPC_{Ar}	1441.1	1.03	0.61

Supplementary Table 6. Free energies for elementary steps involved in CH_3COCH_3 generation from CO_2 reduction on Cu-pyridinic-N_4 and Cu-pyrrolic-N_4 sites of Cu-SA/NPC.

	Elementary steps	ΔG (eV)			
		**Cu-pyridinic-N_4**		**Cu-pyrrolic-N_4**	
		**0 V**	**-0.36 V**	**0 V**	**-0.36 V**
1	$CO_2(g)$ + H+ +e- + * → COOH*	1.66	1.30	1.42	1.06
2	COOH* + H+ + e- → CO* + $H_2O(aq)$	-0.61	-0.97	0.50	0.14
3	2CO* → COCO* + * (non-electrochemical)	1.67	1.67	-1.23	-1.23
4	COCO* + H+ +e- → COCOH*	0.31	-0.05	-0.17	-0.53
5	COCOH* + H+ +e- → COC* + $H_2O(aq)$	-0.62	-0.98	0.29	-0.07
6	COC* + H+ +e- → COCH*	-0.40	-0.76	-0.92	-1.28
7	COCH* + H+ +e- → $COCH_2$*	-0.05	-0.41	-0.19	-0.55
8	$COCH_2$* + H+ +e- → $COCH_3$*	-1.84	-2.20	-0.70	-1.06
9	$COCH_3$* + CO* → $COCOCH_3$* + * (non-electrochemical)	-0.18	-0.18	-0.92	-0.92
10	$COCOCH_3$* + H+ +e- → $COHCOCH_3$*	1.06	0.70	0.32	-0.04
11	$COHCOCH_3$* + H+ +e- → $CCOCH_3$* + $H_2O(aq)$	0.15	-0.21	0.16	-0.20
12	$CCOCH_3$* + H+ +e- → $CHCOCH_3$*	-1.27	-1.63	-1.25	-1.61
13	$CHCOCH_3$* + H+ +e- → CH_2COCH_3*	-0.17	-0.53	-0.18	-0.54
14	CH_2COCH_3* + H+ +e- → $CH_3COCH_3(aq)$ + *	-2.64	-3.00	-1.77	-2.14

Supplementary Table 7. Free energies for elementary steps involved in CH_3COCH_3 generation from CO_2 reduction on uncoordinated pyrrolic-N_1 and pyrrolic-N_4 sites.

	**Elementary steps**	**ΔG (eV)**	
		pyrrolic-N_1	pyrrolic-N_4
		**0 V**	**0 V**
1	CO_2(g) + H+ +e- + * → COOH*	0.75	1.35
2	COOH* + H+ + e- → CO* + H_2O(aq)	1.24	0.27
3	2CO* → COCO* + * (non-electrochemical)	-0.79	0.24
4	COCO* + H+ +e- → COCOH*	0.70	-0.08
5	COCOH* + H+ +e- → COC* + H_2O(aq)	-0.04	1.17
6	COC* + H+ +e- → COCH*	-0.92	-1.60
7	COCH* + H+ +e- → $COCH_2$*	-1.07	-0.68
8	$COCH_2$* + H+ +e- → $COCH_3$*	-1.28	1.07
9	$COCH_3$* + CO* → $COCOCH_3$* + * (non-electrochemical)	-0.66	1.05
10	$COCOCH_3$* + H+ +e- → $COHCOCH_3$*	0.38	0.38
11	$COHCOCH_3$* + H+ +e- → $CCOCH_3$* + H_2O(aq)	0.97	-0.50
12	$CCOCH_3$* + H+ +e- → $CHCOCH_3$*	-1.55	-1.29
13	$CHCOCH_3$* + H+ +e- → CH_2COCH_3*	-1.21	0.01
14	CH_2COCH_3* + H+ +e- → CH_3COCH_3(aq) + *	-0.07	0.28

Supplementary Table 8. Free energies for elementary steps involved in CH_3COCH_3 generation from CO_2 reduction on pyridinic-N=O and pyrrolic-N=O sites.

	**Elementary steps**	**ΔG (eV)**			
		**pyridinic-N=O**		**pyrrolic-N=O**	
		**0 V**	**-0.36 V**	**0 V**	**-0.36 V**
1	CO_2(g) + H+ +e- + * → COOH*	-0.43	-0.79	0.10	-0.26
2	COOH* + H+ + e- → CO* + H_2O(aq)	-0.34	-0.70	-0.29	-0.65
3	2CO* → COCO* + * (non-electrochemical)	1.05	1.05	0.02	0.02
4	COCO* + H+ +e- → COCOH*	0.55	0.19	-0.47	-0.83
5	COCOH* + H+ +e- → COC* + H_2O(aq)	0.34	-0.02	-1.15	-1.51
6	COC* + H+ +e- → COCH*	-0.88	-1.24	0.54	0.18
7	COCH* + H+ +e- → $COCH_2$*	-0.64	-1.00	0.20	-0.16
8	$COCH_2$* + H+ +e- → $COCH_3$*	0.18	-0.18	0.82	0.46
9	$COCH_3$* + CO* → $COCOCH_3$* + * (non-electrochemical)	1.62	1.62	0.80	0.80
10	$COCOCH_3$* + H+ +e- → $COHCOCH_3$*	1.06	0.69	0.48	0.12
11	$COHCOCH_3$* + H+ +e- → $CCOCH_3$* + H_2O(aq)	-1.48	-1.84	-0.82	-1.18
12	$CCOCH_3$* + H+ +e- → $CHCOCH_3$*	-0.64	-1.00	-0.39	-0.75
13	$CHCOCH_3$* + H+ +e- → CH_2COCH_3*	0.32	-0.04	-0.34	-0.70
14	CH_2COCH_3* + H+ +e- → CH_3COCH_3(aq) + *	0.01	-0.35	0.06	-0.30

Supplementary Table 9. Free energies for elementary steps involved in HCOOH, CH_3COOH, CH_3OH and C_2H_5OH generation from CO_2 reduction on the Cu-pyrrolic-N_4 site of Cu-SA/NPC.

Elementary steps		ΔG (eV)	
		**0 V**	**-0.36 V**
	**HCOOH**		
1	CO_2(g) + H+ +e- + * → HCOO*	2.42	2.06
2	HOOH* + H+ + e- → HCOOH*	-1.55	-1.91
3	HCOOH* → HCOOH(aq) + * (non-electrochemical)	-0.47	-0.47
	**CH_3COOH**		
1	CO_2(g) + H+ +e- + * → HCOO*	1.42	1.06
2	COOH* + H+ + e- → CO* + H_2O(aq)	0.50	0.14
3	CO* + H+ + e- → COH*	0.98	0.62
4	COH* + H+ +e- → C* + H_2O(aq)	0.59	0.23
5	C* + H+ +e- → CH*	-0.78	-1.14
6	CH* + H+ +e- → CH_2*	-1.94	-2.30
7	CH_2* + H+ +e- → CH_3*	-0.42	-0.78
8	CH_3* + COOH* → CH_3COOH* + * (non-electrochemical)	-1.37	-1.37
9	CH_3COOH* → CH_3COOOH(aq) + * (non-electrochemical)	-0.61	-0.61
10	CO* + H+ + e- → CHO*	-0.50	-0.86
11	CHO* + H+ + e- → CHOH*	0.18	-0.18
12	CHOH* + H+ + e- → CH* + H_2O(aq)	1.12	0.76
	**CH_3OH**		
1	CO_2(g) + H+ +e- + * → HCOO*	1.42	1.06
2	COOH* + H+ + e- → CO* + H_2O(aq)	0.50	0.14
3	CO* + H+ + e- → CHO*	-0.50	-0.86

4	$CHO^* + H^+ + e^- \rightarrow CH_2O^*$	-0.12	-0.48
5	$CH_2O^* + H^+ + e^- \rightarrow CH_3O^*$	0.73	0.37
6	$CH_3O^* + H^+ + e^- \rightarrow CH_3OH^*$	-1.44	-1.80
7	$CH_3OH^* \rightarrow CH_3OH(aq) + ^*$ (non-electrochemical)	-0.39	-0.39
8	$CH_2O^* + H^+ + e^- \rightarrow CH_2OH^*$	0.17	-0.19
9	$CH_2OH^* + H^+ + e^- \rightarrow CH_3OH^*$	-0.88	-1.24
10	$CHO^* + H^+ + e^- \rightarrow CHOH^*$	0.18	-0.18
11	$CHOH^* + H^+ + e^- \rightarrow CH_2OH^*$	-0.13	-0.49
	**C_2H_5OH**		
1	$CO_2(g) + H^+ + e^- + ^* \rightarrow HCOO^*$	1.42	1.06
2	$COOH^* + H^+ + e^- \rightarrow CO^* + H_2O(aq)$	0.50	0.14
3	$2CO^* \rightarrow COCO^* + ^*$ (non-electrochemical)	-1.23	-1.23
4	$COCO^* + H^+ + e^- \rightarrow COCOH^*$	-0.22	-0.58
5	$COCOH^* + H^+ + e^- \rightarrow COCHOH^*$	0.21	-0.15
6	$COCHOH^* + H^+ + e^- \rightarrow COCH_2OH^*$	-0.55	-0.91
7	$COCH_2OH^* + H^+ + e^- \rightarrow CHOCH_2OH^*$	-0.50	-0.86
8	$CHOCH_2OH^* + H^+ + e^- \rightarrow CHOHCH_2OH^*$	0.79	0.43
9	$CHOHCH_2OH^* + H^+ + e^- \rightarrow CHCH_2OH^* + H_2O(aq)$	-0.22	-0.58
10	$CHCH_2OH^* + H^+ + e^- \rightarrow CH_2CH_2OH^*$	-0.87	-1.23
11	$CH_2CH_2OH^* + H^+ + e^- \rightarrow CH_3CH_2OH^*$	-1.16	-1.52
12	$CH_3CH_2OH^* \rightarrow CH_3CH_2OH(aq) + ^*$ (non-electrochemical)	-0.35	-0.35

Supplementary Notes

Supplementary Note 1. Impact of Zn on acetone production

According to the ICP-AES test (Supplementary **Table 1**), Zn impurity existed in the NPC and Cu-SA/NPC. The Zn content of NPC was 0.90 wt %, which was 12 times higher than that of Cu-SA/NPC (0.07 wt %). However, the liquid products of CO_2 reduction on NPC were HCOOH and CH_3COOH (Supplementary **Fig. 13**), demonstrating that Zn should not be responsible for the formation of CH_3OH, C_2H_5OH and CH_3COCH_3. The Zn content of Cu-SA/NPC was only 0.07 wt %, and such low level of Zn on the catalysts could not combine with Cu nor change the selectivity toward CO_2 reduction [1]. In this work, the Cu atom was identified to be coordinated with N atom as measured by the EXAFS (**Fig.2b**), demonstrating that Zn was not coordinated with Cu. As shown in Supplementary **Table 1**, Cu-SA/NPC and Cu-SA/NPC_{Ar} possessed similar Zn content of 0.07 wt % and 0.06 wt %, respectively, but the selectively of CH_3OH, C_2H_5OH and CH_3COCH_3 production on these two catalysts was different. This also indicated that Zn should not be responsible for the formation of CH_3OH, C_2H_5OH and CH_3COCH_3. Furthermore, the Cu content, BET surface area and pore structure (Supplementary **Tables 1, 5** and **Figs. 19, 20**) of Cu-SA/NPC and Cu-SA/NPC_{Ar} catalysts were also similar, therefore, the disparate CO_2 reduction activity of these two catalysts may be resulted from the content and type of N species. Furthermore, the influence of Zn impurity on acetone production from CO_2 reduction on Cu-SA/NPC catalyst was also investigated by DFT calculations.

Supplementary Note 2. Other C-C coupling pathways on Cu-pyrrolic-N_4 by DFT

Other possible C-C coupling pathways were also examined on the Cu-pyrrolic-N_4 site of Cu-SA/NPC, including CO*-CHO* and CO*-COH* coupling, and were compared with the CO*-CO* coupling route. As illustrated in Supplementary **Fig. 23**, the formation of COH* from CO* reduction was 0.98 eV endothermic in free energy change and thus subsequent CO*-COH* coupling was not a preferred path despite that this C-C coupling was substantially exothermic (-2.27 eV). Meanwhile, the CO*

reduction to CHO* (ΔG of -0.50 eV) was less favorable as compared to the direct coupling of two CO* species with a ΔG of -1.23 eV. Moreover, the formed COCHO* species was much less stable than the COCOH* species (Supplementary **Fig. 23**). Therefore, the route going through CO*-CO* coupling followed by the formation of a COCOH* intermediate should be the most plausible pathway for acetone production from CO_2 reduction and was reported in the main text.

Supplementary Note 3. Pyridinic-N=O and pyrrolic-N=O sites examined by DFT

As shown in Supplementary **Table 4**, other than the difference in the content of pyridinic N and pyrrolic N, the oxidized N content was also different on Cu-SA/NPC and Cu-SA/NPC_{Ar}. The experimental results on N-doped porous carbon catalyst (NPC) without adding Cu showed no acetone formation from CO_2 reduction, which suggested that the oxidized N should not be the active site for acetone production on the Cu-SA/NPC catalyst. To further confirm this, DFT calculations of energetic pathways for CO_2 reduction to acetone on two types of oxidized N sites, including pyridinic- and pyrrolic-N=O sites, were further performed. The results showed that the pyridinic-N=O site was not active for CO_2 reduction to acetone since several steps involved in the conversion were highly uphill in ΔG, such as CO*-CO* coupling and CO*-$COCH_3$* coupling steps with ΔG values of 1.05 and 1.62 eV, respectively, at 0 V (see Supplementary **Fig. 26a**). On the pyrrolic-N=O site, although the CO_2 reduction to COOH* step became facile comparing to that occurred on the Cu-pyrrolic-N_4 site, some elementary steps involved in acetone formation had large ΔG values, such as $COCH_2$* reduction to $COCH_3$* and CO*-$COCH_3$* coupling steps with ΔG of ~0.8 eV at 0 V (see Supplementary **Fig. 26b**). Since the C-C coupling reaction could not be facilitated by the applied potential, the acetone formation should not prefer to occur on the pyrrolic-N=O site. It was worth noting that only the reduction of CO_2 to COOH* involved the N=O site to form a N-O-COOH* intermediate, but other steps mainly occurred on adjacent C-N sites or C-N···N-C bridge sites with less O atom interaction on the two types of oxidized N sites (all intermediates involved in acetone formation were provided in Supplementary **Figs. 27** and **28**). Therefore, the oxidized N sites were not

the active sites for acetone formation.

Supplementary Note 4. Zn-involved sites examined by DFT

To further investigate whether the Zn species had an impact on acetone production from CO_2 reduction on Cu-SA/NPC, DFT calculations were performed on two types of catalyst models including Zn-pyrrolic-N_4 and (Cu-Zn)-pyrrolic-N_3 sites (Supplementary **Figs. 29** and **30**). The Gibbs free energy change for several key elementary steps were calculated including CO_2 reduction to COOH* and then to CO*, together with two non-electrochemical C-C coupling reactions, and compared with that obtained on Cu-pyrrolic-N_4. As shown in Supplementary **Fig. 29**, on Cu-pyrrolic-N_4 site without Zn, the first eletroreduction step of CO_2 to COOH* had a ΔG value of 1.42 eV, and was identified to be the rate-limiting step for acetone formation according to **Fig. 5**. The two C-C coupling reactions of CO*+CO* and CO*+$COCH_3$* were energetically exothermic with ΔG values of -1.23 and -0.92 eV, respectively, indicating a facile formation of acetone from CO_2 reduction on Cu-pyrrolic-N_4. For comparison, on Zn-pyrrolic-N_4 without Cu, the first electroreduction step of CO_2 to COOH* was energetically more endothermic with a ΔG value of 1.53 eV. In addition, the C-C coupling of two CO* species had a ΔG value of 0.96 eV and this reaction could not be facilitated by the electrode potential. Therefore, the Zn-pyrrolic-N_4 was not active for acetone production. Although the (Cu-Zn)-pyrrolic-N_3 facilitated CO_2 reduction to COOH* by reducing the ΔG value to 0.73 eV, the non-electrochemical C-C coupling of two CO* was highly endothermic with a ΔG value of 1.92 eV, as illustrated in Supplementary **Fig. 29**. These DFT results revealed that the Zn species was not responsible for acetone production from CO_2 reduction, confirming the experimental results that the trace amount of Zn impurity had a negligible effect on CO_2 reduction on Cu-SA/NPC.

Supplementary Methods

Materials

Zinc nitrate hexahydrate ($Zn(NO_3)_2 \cdot 6H_2O$, 99%) and copper acetate monohydrate ($Cu(CH_3COO)_2 \cdot H_2O$, 99%) were obtained from Sinopharm Chemical Reagent Co. Ltd. 2-methylimidazole (99%) was purchased from Aladdin Industrial Corporation. Potassium bicarbonate ($KHCO_3$) was obtained from Tianjin Fuchen Chemical Reagent CO. Ltd. Nafion N117 membrane and Nafion solution (5%) were supplied by DuPont Ltd. Carbon paper (HCP 330P) was purchased from Hesen Electrical Co.Ltd. All chemicals were of analytical grade and used without further treatment. The ultrapure water was used for all experiments.

Synthesis of ZIF-8

For synthesis of ZIF-8, 5.256 g of 2-methylimidazole and 4.76 g of $Zn(NO_3)_2 \cdot 6H_2O$ were dissolved in 80 mL of methanol and 120 mL of methanol, respectively. After ultrasound for 10 min, the $Zn(NO_3)_2 \cdot 6H_2O$ solution was added into the 2-methylimidazole solution under stirring for 30 min at room temperature. Subsequently, the mixture was transferred into Teflon-lined autoclave and heated at 120 ℃ for 4 h. The products were washed with methanol and DMF several times. The white powder was dried at 80 ℃ under vacuum.

Synthesis of porous carbon

The obtained ZIF-8 powder was placed into a tube furnace and carbonized at 1000 ℃ for 4 h under nitrogen (N_2) atmosphere. The heating rate was set to 5 ℃ min^{-1}. The sample was denoted as NPC.

Preparation of working electrode

The working electrode was synthesized as follow. 10 mg of obtained powder was added into 3 mL mixture solution, which contained 2.95 mL of H_2O and 0.05 mL of Nafion (5%). After ultrasound for 30 min, the catalyst ink was coated on the carbon paper with geometric area of 6 cm^2. The catalyst ink was dried at room temperature and then heated at 120 ℃ for 4 h to remove volatile compound. For CV tests, the working electrode was glassy carbon electrode with geometric area of 0.07 cm^2 covered by

coating 10 μL of the above catalyst ink.

Analytical method

The gas products were analyzed by gas chromatograph (Shimadzu, GC-14C). Liquid products were identified by mean of ^{1}H nuclear magnetic resonance with a 500 MHz spectrometer (Rruker, AVANCE III 500). The solvent presaturation method was used to suppress the water peak. Quantization of formic acid and acetic acid was measured by using an ion chromatography (Shimadzu, SCL-10ASP). Methanol, ethanol and acetone were quantified by gas chromatography equipped a flame ionization detector (Shimadzu, GC-2010). The ^{13}C-labeled product was analyzed by gas chromatography-mass spectrometry (GC-MS, Agilent 6890N GC, America) equipped with an Agilent HP-5MS column (30 m × 0.250 mm × 0.25 μm).

XPS analysis

The XPSPEAK41 software was used to analyze the obtained XPS spectra. Component fitting for N element was based on Gaussian-Lorentzian product function with a 20% Lorentzian-Gaussian value using Shirley background. The C 1s at 284.5 eV was used as the reference for charge correction. The percentage of different N species was determined by the ratio of peak area. The binging energies (BE) and full width at half maximum (FWHM) was fixed at constant.

Supplementary References

1. Karapinar D., Huan N. T., Ranjbar Sahraie N., Li J.K., Wakerley D, Touati N., Zanna S., Taverna D., Tizei L. H. G., Zitolo A., Jaouen F., Mougel V., Fontecave M. Electroreduction of CO_2 on single-site copper-nitrogen-doped carbon material: selective formation of ethanol and reversible restructuration of the metal sites. *Angew. Chem. Int. Ed.* **58**, 15098-15103 (2019).

2. Jung, H., Lee, S. Y., Lee, C. W., Cho, M. K., Won, D. H., Kim, C., Oh. H., Min, B. K., Hwang, T. J. Electrochemical fragmentation of Cu_2O nanoparticles enhancing selective C-C coupling from CO_2 reduction reaction. *J. Am. Chem. Soc.* **141**, 4624-4633 (2019).

3. Yuan, J., Zhi, W. Y., Liu, L., Yang M. P., Wang, H., Lu, J. X. Electrochemical reduction of CO_2 at metal-free N-functionalized graphene oxide electrodes. *Electrochim. Acta* **282**, 694-701 (2018).

4. Yuan, J., Zhang, J. J., Yang, M. P., Meng, W. J., Wang, H., Lu, J. X. CuO nanoparticles supported on TiO_2 with high efficiency for CO_2 electrochemical reduction to ethanol. *Catalysts* 8, 171 (2018).

5. Klingan, K., Kottakkat, T., Jovanov, Z. P., Lovanov, Z. P., Jing, S., Pasquini, C., Scholten, F., ubella, P., Bergmann, A., Cuenya, B. R., Roth, C., Dau, H. Reactivity determinants in electrodeposited Cu foams for electrochemical CO_2 reduction. *ChemSusChem* **11**, 3449-3459 (2018).

6. Marepally, B. C., Ampelli, C., Genovese, C., Tavella, F., Veyre, L., Quadrelli, E. A., Perathoner, S., Centi, G. Role of small Cu nanoparticles in the behaviour of nanocarbon-based electrodes for the electrocatalytic reduction of CO_2. *J. CO_2 Util.* **21**, 534-542 (2017).

7. Marepally, B. C., Ampelli, C., Genovese, C., Saboo, T., Perathoner, S., Wisser, F. M., Veyre, L., Canivet, J., Quadrelli, E. A., Centi, G. Enhanced formation of > C1 products in electroreduction of CO_2 by adding a CO_2 adsorption component to a gas-diffusion layer−type catalytic electrode. *ChemSusChem* **10**, 4442-4446 (2017).

8. Popić, J. P., Avramov-Ivić, M. L., Vuković, N. B. Reduction of carbon dioxide on ruthenium oxide and modified ruthenium oxide electrodes in 0.5 M $NaHCO_3$. *J. Electroanal. Chem.* **421**, 105-110 (1997).

9. Genovese, C., Ampelli, C., Perathoner, S., Centi, G. Electrocatalytic conversion of CO_2 on carbon nanotube-based electrodes for producing solar fuels. *J. Catal.* **308**, 237-249 (2013).

10. Kuhl, K. P., Cave, E. R., Abram, D. N., Jaramillo, T. F. New insights into the electrochemical reduction of carbon dioxide on metallic copper surfaces. *Energy Environ. Sci.* **5**, 7050-7059 (2012).

11. Ogura, K., Sugihara, H., Yano, J., Higasa, M. Electrochemical reduction of carbon dioxide on dual−film electrodes modified with and without cobalt (II) and iron (II) complexes. *J. Electrochem. Soc.* **141**, 419-424 (1994).

12. Ogura, K., Endo, N. Electrochemical reduction of CO_2 with a functional gas−diffusion electrode in aqueous solutions with and without propylene carbonate. *J. Electrochem. Soc* **146**, 3736-3740 (1999).

13. Le Duff, C. S., Lawrence, M. J., Rodriguez, P. Role of the adsorbed oxygen species in the selective electrochemical reduction of CO_2 to alcohols and carbonyls on copper electrodes. *Angew. Chem. Int. Ed.* **56**, 12919-12924 (2017).
