# Supplementary material for: Selective electroreduction of CO2 to acetone by single copper atoms anchored on N-doped porous carbon
Source: Nat Commun. 2020 May 15;11:2455. doi: 10.1038/s41467-020-16381-8 (PMC7229121; doi:10.1038/s41467-020-16381-8)
Supplement: Supplementary file 2 — Peer Review File [file 41467_2020_16381_MOESM2_ESM.docx]

Peer Review Information

**Journal:** Nature Communications

**Manuscript title:** Selective electrochemical reduction of CO_2_ to acetone by single Cu atoms anchored on N-doped porous carbon

**Corresponding author name(s):** Prof Jingguang Chen

**Editorial notes:**

**Transferred manuscripts**

This manuscript has been previously reviewed at another journal that is not operating a transparent peer review scheme. This document only contains reviewer comments and rebuttal letters for versions considered at *Nature Communications*.

**Transferred manuscripts (no peer review at *Nature Communications*)**

This manuscript has been previously reviewed at another journal that is not operating a transparent peer review scheme. The manuscript was considered suitable for publication without further review at *Nature Communications*.

**Redactions – transferred manuscripts (mention of previous referee reports from elsewhere)**

This manuscript has been previously reviewed at another journal that is not operating a transparent peer review scheme. This document only contains reviewer comments and rebuttal letters for versions considered at *Nature Communications*. Mentions of prior referee reports have been redacted.

**Redactions – transferred manuscripts (mention of the other journal)**

This manuscript has been previously reviewed at another journal that is not operating a transparent peer review scheme. This document only contains reviewer comments and rebuttal letters for versions considered at *Nature Communications*. Mentions of the other journal have been redacted.

**Redactions – unpublished data**

Parts of this Peer Review File have been redacted as indicated to maintain the confidentiality of unpublished data.

**Redactions – confidential patient information**

Parts of this Peer Review File have been redacted as indicated to maintain patient confidentiality.

**Redactions – published data**

Parts of this Peer Review File have been redacted as indicated to remove third-party material where no permission to publish could be obtained.

**Amendments – published data**

[Figure X/The figure on page X] in this Peer Review File has been amended to remove third-party material where no permission to publish could be obtained.

**Published data (credit line for image)**

[Figure X/The figure on page X] in this Peer Review File is reproduced with permission from [Copyright Holder].

**Redactions – reviewer opt-out**

Parts of this Peer Review File have been redacted as indicated as we could not obtain permission to publish the reports of reviewer ###.

**Reviewer comments in marked-up manuscript**

In their review of the [first/second/third/...] version of this manuscript, reviewer ### added their comments to the manuscript file. These comments, excluding minor textual revisions, have been copied into this Peer Review File.

**Reviewer comments & decisions:**

**Reviewer comments, first version:**

Reviewers' comments:

Reviewer #1 (Remarks to the Author):

In this work, the authors reported single Cu atom sites on N-containing carbon derived from MOF structures for the CO2 reduction to multi C atoms products.
This manuscript is of limited novelty but reports some interesting results on the impact of the N species on C, however the analysis of the data is incomplete and thus the theoretical calculation is based on a too narrow scenario, which could lead to inadequate conclusions.
Particularly, the rationalization of the results aided by the calculations is based on the assumption that the XPS analysis and the assignment of the components in the N1s XPS are exact and unique.
However, based on many experimental data in the literature, whilst the lowest BE component in the N1s can be safely assigned to uncoordinated pyridinic N, and the highest BE component to quaternary N, the assignment of the components in between is highly ambiguous, and must be checked case by case with specifically addressed experiments. Particularly, at 399.5 eV circa there are metal-Npyr species, whereas at 400 eV, not only pyrrole is found but also oxygenated forms of N such as lactame and pyridine oxide.
If one considers that the only difference in the preparation of the two electrocatalysts was the atmosphere, Air vs Ar, one can think of having an oxygenated N species rather than pyrrole in the most active electrocatalysts. Also it could be just the result of having more Cu coordinated. Indeed, additional components can be used to fit the spectra reported by the authors.
Therefore, a more detailed N and Cu speciations of the catalysts is recommended to clearly identify the nature of the N species and the Cu coordination environment. Following, it would be interesting to see the energetic scenario of additional metal-N ensembles with respect to the reaction intermediates by computation. Based on the points raised above I recommend publication but only after major revision.
The catalytic data part is also quite limited and the authors could include a more extended analysis of the current vs potential, since it is known that the CO2RR for products >C1 follows a volcano type curve with maximum that differs from catalyst to catalyst. Indeed analysis of the reactivity at the maximum could be the ideal way of rationalizing catalytic data, rather than looking at certain potentials.
Some of the graphics needs improvements as for example Figure 1f is too small.


Reviewer #2 (Remarks to the Author):

This study shows acetone formation from electrochemical CO2 reduction on Cu single atoms embedded N-doped porous carbon (Cu-SA/NPC). Various C1-C3 oxygenates including HCOOH, CH3COOH, CH3OH, C2H5OH and CH2COCH3 are produced in CO2 reduction on Cu-SA/NPC. Among them, acetone (CH3COCH3) is the most produced. The results seem too preliminary due to too many unclear issues.

1. The authors suggest that m/z = 61 not observed in Fig. S6 may be caused by the participation of HCO3- electrolyte (Fig. S7). The main peak in GC-MS during 13CO2 reduction is m/z = 58 (Fig. S6b), however, the m/z = 58 indicates that acetone is produced from coupling of three H12CO3- and none of the 13CO2 feed gas is involved. In other words, acetone is produced from HCO3- reduction present in solution, not CO2 feed-reduction. In this case, CO2 reduction is not a main subject, but basically conversion of solution KHCO3 into aceton.

2. When using 13CO2 or H13CO3-, what m/z ratios are observed in other products? Does the HCO3- participate in formation of other products?

3. One of the key findings in this study is selective acetone formation among various oxygenates. However, the origin of selective acetone formation is not discussed deeply. In DFT calculations, what is the selectivity determining step of acetone versus other oxygenates and free energy change at this step?

4. The C-C coupling and acetone formation pathway considered here are just one of several possible pathway. For example, only the CO-CO coupling is considered here in C-C coupling, while CO-CHO and CO-COH are also possible mechanism. Justification of the reaction pathway considered here, or other pathways should be considered to find the most plausible pathway.

5. The production rates of HCOOH and CH3COOH on N-doped porous carbon (NPC) are 200~300 μg h-1 at -0.76 V (Fig. S8), while those on Cu-SA/NPC are less than 150 μg h-1 at same potential (Fig. 3b). This result indicates that HCOOH and CH3COOH produced from NPC may further reduced to acetone on Cu-SA/NPC.

6. It is unclear that the adsorbents are bound to Cu or N atom in Cu-SA/NPC (Fig. 5d). Further clarification and top-view of geometries should be provided.


Reviewer #3 (Remarks to the Author):

The authors have presented an interesting Cu single-atom catalyst with Cu atoms coordinated on N-doped carbon derived from ZIF-8. The Cu-SAC was found to be highly active for producing acetone. Acetone is not a common product in CO2 reduction, but isotope labeling has proven the production of acetone. Several questions remain elusive:

1. The author needs to provide explanation on the absence of gaseous carbon-based product, such as CO, CH4 and C2H4. *CO is in the proposed pathway and it’s unusual to see no CO is released at all from a Cu-based catalyst.
2. What’s the active site? It is hard to be convinced that a single Cu site could enable C-C coupling and even stabilize C3 intermediates. CO coverage on a continuous Cu surface is thought to be highly important for C-C coupling step, which requires neighboring *CO adsorbates (DOI: 10.1021/jacs.7b03300, DOI: 10.1038/s41467-018-07970-9)
3. More control experiments are needed. Did the coordinated N-atoms participate in the reaction? Or the reaction require synergy between two adjacent Cu sites? Comparative studies of, such as NPC and Cu-SA/NPC with different Cu loadings should be done to study the role of different active sites.
4. For the mechanism discussed in Figure 5, did the authors compare competitive pathways? Can a C1 or C2 product be more favorable than acetone?
5. Was the catalyst stable during the reaction? More characterizations and comparisons are needed for the catalysts before and after the measurements.
6. The discussion of HCO3- participation in the reaction is somewhat elusive. Can the authors provide more insights into the connection to the MS spectra?

**Author rebuttal, first version:**

*In this work, the authors reported single Cu atom sites on N-containing carbon derived from MOF structures for the CO_2_ reduction to multi C atoms products.*

*This manuscript is of limited novelty but reports some interesting results on the impact of the N species on C, however the analysis of the data is incomplete and thus the theoretical calculation is based on a too narrow scenario, which could lead to inadequate conclusions.*

**Response:** We thank the reviewer for considering “this work reports some interesting results on the impact of the N species on C”. To improve the manuscript, we performed additional experimental measurements and DFT calculations to provide sufficient evidence and to strengthen the conclusions. Detailed responses to each of the reviewer’s specific comments are provided below.

*1. Particularly, the rationalization of the results aided by the calculations is based on the assumption that the XPS analysis and the assignment of the components in the N1s XPS are exact and unique. However, based on many experimental data in the literature, whilst the lowest BE component in the N1s can be safely assigned to uncoordinated pyridinic N, and the highest BE component to quaternary N, the assignment of the components in between is highly ambiguous, and must be checked case by case with specifically addressed experiments. Particularly, at 399.5 eV circa there are metal-Npyr species, whereas at 400 eV, not only pyrrole is found but also oxygenated forms of N such as lactame and pyridine oxide.*

**Response:** According to the reviewer’s comments, further deconvolution of N 1s XPS was conducted. As shown in **Fig. R1** and **Table R1** below, the content of graphitic N and Cu-N was similar for the two compared catalysts (Cu-SA/NPC and Cu-SA/NPC_Ar_). However, the content of pyridinic N, pyrrolic N and oxidized N was different, which may dictate the different activities of the two compared catalysts. The N-doped porous carbon catalyst without Cu (NPC) was synthesized by carbonization of ZIF-8 at N_2_ atmosphere for 4 h (the carbonization condition was the same with the Cu-SA/NPC catalyst). The XPS results **(Fig. R1**) confirmed the coexistence of graphitic N, pyridinic N, pyrrolic N and oxidized N. It is worth noting that the oxidized N content of NPC was 1.1%, similar to that of Cu-SA/NPC (1.1%). However, there was no acetone production observed on all N-doped porous carbon catalysts without Cu, which suggested that the oxidized N may not be the active site for acetone production. To further confirm the main active sites for selective acetone formation on the Cu-SA/NPC catalyst, additional DFT calculations of energetic pathways for acetate formation from CO_2_ reduction on two types of oxidized N sites (pyridinic-N=O and pyrrolic-N=O sites) were conducted. The DFT results (**Fig. R2** below) showed that the oxidized N sites were not active for acetone formation. The additional experimental and DFT results helped confirm that the main active sites for acetone synthesis from CO_2_ reduction on the Cu-SA/NPC catalyst were the Cu-pyrrolic-N_4_ sites, as described in the “**Action**” below.

**Fig. R1** XPS 1N 1s spectra of **c** Cu-SA/NPC and **d** Cu-SA/NPC_Ar_. (This figure has been added into the revised manuscript as new Fig. 4c and d.)

**Table R1**. The content of Cu and N of prepared catalysts *^a^*. (This table has been added into the revised Supporting Information as new Table S4.)

| Sample | Cu_Total_  (at.%) | N_Total_  (at.%) | Pyridinic N (at.%) | Pyrrolic N  (at.%) | Graphitic N (at.%) | Cu-N  (at.%) | N-O  (at.%) |
| --- | --- | --- | --- | --- | --- | --- | --- |
| NPC | -- | 7.8 | 2.7 | 2.2 | 1.8 | -- | 1.1 |
| Cu-SA/NPC_0.5_ | 0.2 | 8.3 | 2.6 | 1.5 | 2.0 | 1.5 | 0.7 |
| Cu-SA/NPC | 0.2 | 8.4 | 1.5 | 1.9 | 2.4 | 1.5 | 1.1 |
| Cu-SA/NPC_2_ | 0.3 | 6.5 | 1.3 | 1.5 | 1.6 | 1.5 | 0.6 |
| Cu-SA/NPC_3_ | 0.3 | 6.4 | 1.3 | 1.3 | 1.6 | 1.4 | 0.8 |
| Cu-SA/NPC_Ar_ | 0.2 | 8.2 | 2.6 | 1.4 | 2.0 | 1.6 | 0.6 |

*^a^* Catalysts with adding Cu content of 0, 0.16, 0.31, 0.62, 0.93 and 1.86 g were denoted as NPC, Cu-SA/NPC_0.5_, Cu-SA/NPC, Cu-SA/NPC_2_, Cu-SA/NPC_3_, respectively. Catalysts carbonized at Ar atmosphere was denoted as Cu-SA/NPC_Ar_.

**Fig. R2**. Free energy diagrams calculated at potentials of 0 and -0.36 V for CO_2_ reduction to CH_3_COCH_3_ on **a** pyridinic-N=O and **b** pyrrolic-N=O sites. The two computational models are included in the figure. (This figure has been added into the revised Supporting Information as new Fig. S28.)

**Action:** To incorporate these new experimental and DFT results, the following discussions were added into the revised manuscript in the Section “**Electrochemical reduction of CO_2_ on Cu-SA/NPC**” on **Page 13** and the Section “**Pyridinic-N=O and pyrrolic-N=O sites**” on **Pages 17-19** as:

**Page 13** “*Both spectra exhibited five peaks with binding energies at 398.2, 399.8. 400.1, 401.7 and 403.5 eV, which were assigned to pyridinic N, metal Cu-N, pyrrolic N, graphite N and oxidized N (N-O), respectively^34^. The content of graphite N and metal Cu-N was similar, while the pyridinic N, pyrrolic N, and oxidized N (N-O) was different. As shown in* ***Table S4****, the NPC possessed the oxidized N content of 1.1%, similar to that of Cu-SA/NPC (1.1%). However, there was no acetone production from CO_2_ reduction on NPC without Cu (****Fig. S13****). Therefore, the oxidized N might not play an important role in acetone production from CO_2_ reduction. According to the* ***Table S4****, the content of pyridinic N and pyrrolic N on Cu-SA/NPC was 1.5% and 1.9%, respectively, while the content on* *Cu-SA/NPC_Ar_ was 2.6% and 1.4%, respectively. This comparison indicated that the Cu-SA/NPC had a higher content of pyrrolic N than that of Cu-SA/NPC_Ar_. In view of CO_2_ reduction measurements and XPS results, the enhanced CO_2_ reduction activity and CH_3_COCH_3_ selectivity of Cu-SA/NPC should be attributed to the presence of single atom Cu coordinated with more pyrrolic N that served as active sites for CO_2_ reduction*.”

**Pages 20-21** “***Pyridinic-N=O and pyrrolic-N=O sites****. As shown in* ***Table S4****, the content of graphitic N and Cu-N was similar for the two compared catalysts. However, the content of pyridinic N, pyrrolic N and oxidized N was different, which may result in the different activities of the two compared catalysts. The experimental results on N-doped porous carbon catalysts without adding Cu showed no acetone formation from CO_2_ reduction, which suggested that the oxidized N would not be the active site for acetone production on the Cu-SA/NPC catalyst. To further confirm the main active sites for the selective acetone formation on Cu-SA/NPC, DFT calculations of energetic pathways for CO_2_ reduction to acetate on two types of oxidized N sites, including pyridinic- and pyrrolic-N=O sites, were further performed. The results showed that the pyridinic-N=O site was not active for CO_2_ reduction to acetone since several steps involved in the conversion were highly uphill in ΔG, such as CO*-CO* coupling and CO*-COCH_3_* coupling steps with ΔG values of 1.05 and 1.62 eV, respectively, at 0 V (see* ***Fig. S28a****). On the pyrrolic-N=O site, although the CO_2_ reduction to COOH* step became facile comparing to that occurred on the Cu-pyrrolic-N_4_ site, some elementary steps involved in acetone formation had large ΔG values, such as COCH_2_* reduction to COCH_3_* and CO*-COCH_3_* coupling steps with ΔG of ~0.8 eV at 0 V (see* ***Fig. S28b****). Since the C-C coupling reaction could not be facilitated by the applied potential, the acetone formation should not prefer to occur on the pyrrolic-N=O site. It was worth noting that only the reduction of CO_2_ to COOH* involved the N=O site to form a N-O-COOH* intermediate, but other steps mainly occurred on adjacent C-N sites or C-N****^…^****N-C bridge sites with less O atom interaction on the two types of oxidized N sites (all intermediates involved in acetone formation were provided in* ***Fig. S29*** *and* ***S30****). Therefore, the oxidized N sites were not the active sites for acetone formation.*

*These results confirmed that the main active sites for acetone synthesis from CO_2_ reduction on the Cu-SA/NPC catalyst were the Cu-pyrrolic-N_4_, consistent with the structural prediction of Cu/N coordination environment from HAADF-STEM and EXAFS characterization. For CO_2_ reduction to acetone, the formation of reaction intermediates required the synergy between Cu and N sites (****see Fig. 5b****), leading to quite facile C-C coupling toward C_2_ and C_3_ species formation. In addition, the additional experimental results provided in* ***Table S4*** *revealed that the content of Cu dispersed into the NPC catalyst was relatively less, and thus the possibility of forming Cu clusters was small.* *In the literature, it was evidenced that not only Cu metal was beneficial for C-C coupling^41-43^, but the N-doped carbon catalysts also could reduce CO_2_ to C_2_ products via C-C coupling^44-46^. The DFT calculations in the current study clearly demonstrated that CO_2_ reduction to acetone was energetically quite favorable catalyzed by the Cu-pyrrolic-N_4_ site of Cu-SA/NPC due to the crucial Cu-N synergy.*”

*2. If one considers that the only difference in the preparation of the two electrocatalysts was the atmosphere, Air vs Ar, one can think of having an oxygenated N species rather than pyrrole in the most active electrocatalysts. Also it could be just the result of having more Cu coordinated. Indeed, additional components can be used to fit the spectra reported by the authors.*

*Therefore, a more detailed N and Cu speciations of the catalysts is recommended to clearly identify the nature of the N species and the Cu coordination environment. Following, it would be interesting to see the energetic scenario of additional metal-N ensembles with respect to the reaction intermediates by computation.*

**Response:** This question is relevant to **Question #1** from **Reviewer 1**. In order to confirm the different N distribution derived from carbonization of MOF at different atmosphere, the N-doped porous carbon materials without Cu doping were synthesized with carbonization in N_2_ or Ar. The obtained catalysts were denoted as NPC and NPC_Ar_, respectively. As shown in **Fig. R3** and **Table R2** below, the content of total N and graphite N of the two catalyzed was similar, whereas the content of pyridinic N, pyrrolic N and oxidized N was different. These results revealed that the N distribution could be tuned by carbonization at different atmosphere, such as N_2_ and Ar.

To address this question, further deconvolution of N 1s XPS was conducted, and the content of different N-involved species was shown in **Table R1** above. The content of graphitic N and Cu-N was similar for the two compared catalysts (Cu-SA/NPC and Cu-SA/NPC_Ar_). However, the content of pyridinic N, pyrrolic N and oxidized N was different, which may dictate the different activities of the two compared catalysts. DFT calculations in the original manuscript compared two forms of Cu-N sites on the activity toward acetone formation from CO_2_ reduction and revealed that the Cu-pyrrolic-N_4_ site was catalytically more active than the Cu-pyridinic-N_4_ site due to more favorable reaction free energy pathways (see **Fig. 5** in the revised manuscript). The additional DFT calculations performed on two different oxidized N catalysts revealed that the pyridinic-N=O and pyrrolic-N=O sites were not active sites for acetone formation without Cu (see **Fig. R2** above). The additional experimental and DFT results confirmed that the main active sites for acetone synthesis from CO_2_ reduction on the Cu-SA/NPC catalyst were the Cu-pyrrolic-N_4_ sites. According to the combined HAADF-STEM and EXAFS results, it was evidenced that the Cu species was atomically dispersed and was four-fold coordinated with N atoms in Cu SA/NPC. In the literature, the structure of metal single atom coordination to four N atoms has also been reported as active site for CO_2_ activation on metal- and N-doped carbon-based catalysts (*e.g. Nat. Catal.2018, 1, 339-348*).

**Fig. R3** XPS 1N spectra of **a** NPC and **b** NPC_Ar_.

**Table R2.** The content of Cu and N of NPC and NPC_Ar_ catalysts.

| Sample | Total N | Pyridinic N | Pyrrolic N | Graphitic N | N-O |
| --- | --- | --- | --- | --- | --- |
| NPC | 7.8% | 2.7% | 2.2% | 1.8% | 1.1% |
| NPC_Ar_ | 7.6% | 3.4% | 1.7% | 1.9% | 0.6% |

**Action:** To address this question from the reviewer and incorporated the new results, new discussions were added into the revised manuscript in the Section “**Electrochemical reduction of CO_2_ on Cu-SA/NPC**” on **Page 13** and the Section “**Pyridinic-N=O and pyrrolic-N=O sites**” on **Pages 20-21**, as given in the “**Action**” to **Question #1** of **Reviewer 1**.

*Based on the points raised above I recommend publication but only after major revision.*

**Response:** We thank the reviewer for recommending publication of our manuscript in Nature Communications. Based on the suggestions and comments from the reviewer, we have performed additional experiments and DFT calculations to address all the concerns/questions and incorporated the new results into the revised manuscript.

*3. The catalytic data part is also quite limited and the authors could include a more extended analysis of the current vs potential, since it is known that the CO2RR for products >C1 follows a volcano type curve with maximum that differs from catalyst to catalyst. Indeed analysis of the reactivity at the maximum could be the ideal way of rationalizing catalytic data, rather than looking at certain potentials.*

**Response:** According to the suggestion, the current-time curves of electroreduction on Cu-SA/NPC at varied applied potentials in 0.1 M KHCO_3_ solution were provided (**Fig. R4**) and the CO_2_ electroreduction on Cu-SA/NPC was conducted at varied potentials between -0.1 V and -1.0 V vs. RHE (**Fig. R5**). As shown in **Fig. R5**, no acetone was detected at -0.1 V vs. RHE and the onset of acetone production occurred at the potential of -0.16 V and increased with the applied potential negatively shifted from -0.16 to -0.76 V, but the production rate was decreased with the potential further negatively shifted. The Faradic efficiency of acetone generation increased from 23.4% to 36.7% as potential was negatively shifted from -0.16 to -0.36 V, and it then decreased to 13.5% with the potential further negatively shifted to -1.0 V. The maximum acetone production rate of 336.1 ug h^-1^ was achieved at -0.76 V, while the maximum Faradic efficiency of acetone production reached 36.7% at a potential of -0.36 V.

**Action:** To incorporate the new experimental results, the following discussions were added into the revised manuscript in the Section “**Electrochemical reduction of CO_2_ on Cu-SA/NPC**” on **Pages 8-9** as:

**Page 8** “***Fig. 3b*** *showed the product distribution on Cu-SA/NPC, which was found to be dependent on the applied potential (its current-time curves were presented in* ***Fig. S5****).*”

**Page 9** “*Moreover, the production rate of CH_3_COCH_3_ was further investigated in the potentials between -0.1 and -1.0 V (****Fig. S6****). At the potential of -0.1 V, no CH_3_COCH_3_ was detected. The production rate of CH_3_COCH_3_ increased at potentials between -0.16 and -0.76 V, and then decreased with the applied potential further negatively shifted to -1.0 V, likely due to the competing reaction of H_2_ evolution.*”

**Fig. R4** Current-time curves of CO_2_ electroreduction on Cu-SA/NPC at varied applied potential in 0.1 M KHCO_3_ solution. (This figure has been added into the revised Supporting Information as new Fig. S5.)

**Fig. R5** Production rate and Faradic efficiency of acetone production on Cu-SA/NPC at potential of -0.1 to -1.0 V vs. RHE. (This figure has been added into the revised Supporting Information as new Fig. S6.)

*4. Some of the graphics needs improvements as for example Figure 1f is too small.*

**Response:** We thank the review for this suggestion.

**Action:** We accordingly improved the quality of graphics including **Figure 1f** and **Figure 5** in the revised manuscript, as shown below.

**Fig. 1** **a** SEM. **b**, **c** TEM. **d**, **e** HAADF-STEM images and enlarged images. **f-i** EDS images of Cu-SA/NPC by HAADF-STEM.

**Fig. 5** **a** Free energy diagrams calculated at a potential of -0.36 V for CO_2_ reduction to CH_3_COCH_3_ on Cu-pyridinic-N_4_ and Cu-pyrrolic-N_4_ sites of Cu-SA/NPC (the computational models were included in the figure). **b** Optimized structures of all reaction intermediates involved in the pathways of CO_2_ reduction on the Cu-pyrrolic-N_4_ site (grey: C of catalyst; black: C of adsorbate; red: O; orange: Cu; blue: N; white: H).

**~~---------------------------------------------------------------------------------------------------------------------~~**

**Reviewer #2 (Remarks to the Author):**

*This study shows acetone formation from electrochemical CO_2_ reduction on Cu single atoms embedded N-doped porous carbon (Cu-SA/NPC). Various C1-C3 oxygenates including HCOOH, CH_3_COOH, CH_3_OH, C_2_H_5_OH and CH_3_COCH_3_ are produced in CO_2_ reduction on Cu-SA/NPC. Among them, acetone (CH_3_COCH_3_) is the most produced. The results seem too preliminary due to too many unclear issues.*

**Response:** We thank the reviewer for the valuable comments on our manuscript, which enabled us to improve and strengthen our manuscript by performing additional experimental measurements and DFT calculations to incorporate essentially all the comments from the reviewer, as detailed in the below response to each of the specific comments from the reviewer.

*1. The authors suggest that m/z = 61 not observed in Fig. S6 may be caused by the participation of HCO_3_^-^ electrolyte (Fig. S7). The main peak in GC-MS during ^13^CO_2_ reduction is m/z = 58 (Fig. S6b), however, the m/z = 58 indicates that acetone is produced from coupling of three H^12^CO_3_^-^ and none of the ^13^CO_2_ feed gas is involved. In other words, acetone is produced from HCO_3_^-^ reduction present in solution, not CO_2_ feed-reduction. In this case, CO_2_ reduction is not a main subject, but basically conversion of solution KHCO_3_ into aceton.*

**Response:** We apologized for missing detailed explanations of the role of HCO_3_^-^ in CO_2_ reduction. The bicarbonate anions were served as an electrolyte, which has been identified as providing pH buffer and proton donor in the literature. However, recent studied found that CO_2_ in equilibrium with bicarbonates played an essential role in the CO_2_ reduction reaction (*e.g. ACS Energy Lett. 2019, 4, 682-689*), indicating the existence of a dynamic equilibrium between CO_2_ partial pressure and HCO_3_^-^ concentration.

Lin *et al.* pointed out that CO_2_ could be converted to HCO_3_^-^ and used ^13^CO_2_ exchange experiment to estimate the rate of CO_2_ mass transport. In their work, the ^13^CO_2_ gas was used to fill the headspace of the vial with 0.1 M NaH^12^CO_3_ electrolyte under stirring. The ^13^CO_2_/^12^CO_2_ ratio in the headspace was measured by online mass spectrometry. The specific mass transport coefficient was about 0.0015 s^-1^ by measuring the rate of ^13^CO_2_ transfer between gas and liquid phases (*J. Am. Chem. Soc. 2019, 141, 17875-17883*).

**Action:** To address this question, the following discussions were added into the revised manuscript in the Section “**Electrochemical reduction of CO_2_ on Cu-SA/NPC**” on **Page 9** as:

“*These results were consistent with those reported in the literature ^29,30^. According to the previous studies ^29,30^, there should be a dynamic equilibrium between CO_2_ partial pressure and HCO_3_^-^ concentration and the CO_2_ in equilibrium with HCO_3_^-^ was the source for CO_2_ reduction reaction.*”

*2. When using ^13^CO_2_ or H^13^CO_3_^-^, what m/z ratios are observed in other products? Does the HCO_3_^-^ participate in formation of other products?*

**Response:** Based on previous studies in the literature (*e.g. ACS Energy Lett. 2019, 4, 682-689；J. Am. Chem. Soc. 2019, 141, 17875-17883.*) and our experimental results, the CO_2_ in equilibrium with HCO_3_^-^ was the source for CO_2_ reduction reaction. Therefore, the bicarbonate anions should participate in the CO_2_ reduction reaction. The ^13^C-labeled acetone products were detected by GC-MS using headspace sampling. However, it was difficult to detect the minor acids by headspace sampling due to their high boiling point and high solubility in water. The methanol (C_1_ product) and ethanol (C_2_ product) yields from CO_2_ reduction in ^13^CO_2_ or H^13^CO_3_^-^ were analyzed by GC-MS. As shown in **Fig. R6** below, both the *m/z*=31 and *m/z*=32, characteristic peaks of CH_3_OH and ^13^C-labeled CH_3_OH, respectively, were observed in the spectra of ^13^CO_2_ or H^13^CO_3_, indicating that HCO_3_^-^ was involved in CH_3_OH formation. The peaks at *m/z*=28 and *m/z*=29, as characteristic peaks of ^13^C-labeled C_2_H_5_OH, were observed in ^13^CO_2_ reduction, while *m/z*=27, a characteristic peak of C_2_H_5_OH, was also detected. In the spectra of H^13^CO_3_^-^, the peaks at *m/z*=28 was also detected. These results demonstrated that HCO_3_^-^ anions participated in the CO_2_ reduction reaction to produce acetone.

**Action:** The following text was added into the revised manuscript in the Section “**Electrochemical reduction of CO_2_ on Cu-SA/NPC”** on **Page 9** as:

“*As shown in* ***Fig. S11****, the characteristic peaks of CH_3_OH (m/z=31 and m/z=32) and C_2_H_5_OH (m/z=27, m/z=28 and m/z=29) were detected in ^13^CO_2_ and H^13^CO_3_. These results were consistent with those reported in the literature ^29,30^. According to the previous studies ^29,30^, there should be a dynamic equilibrium between CO_2_ partial pressure and HCO_3_^-^ concentration and the CO_2_ in equilibrium with HCO_3_^-^ was the source for CO_2_ reduction reaction.”*

**Fig. R6** GC-MS spectra of **a** CH_3_OH and **b** C_2_H_5_OH production form ^13^CO_2_ electrochemical reduction, **c** CH_3_OH and **d** C_2_H_5_OH production from CO_2_ electrochemical reduction in H^13^CO_3_^-^. (This figure has been added into the revised Supporting Information as new Fig. S11.)

*3. One of the key findings in this study is selective acetone formation among various oxygenates. However, the origin of selective acetone formation is not discussed deeply. In DFT calculations, what is the selectivity determining step of acetone versus other oxygenates and free energy change at this step?*

**Response:** We thank the reviewer for this constructive comment, and accordingly performed additional DFT calculations to examine the energetic pathways for the formation of other oxygenate products including HCOOH, CH_3_COOH, CH_3_OH and C_2_H_5_OH as observed experimentally (see **Fig. R7a**-**R7d** below). The relative selectivities of these C_1_, C_2_ and C_3_ oxygenates produced on the Cu-pyrrolic-N_4_ site of Cu-SA/NPC were evaluated based on additional DFT calculations (see **Fig. R7e** below) and the origin of selective acetone formation on Cu-SA/NPC was discussed in the revised manuscript (see “**Action**” below).

**Fig. R7** Free energy diagrams calculated for CO_2_ reduction to **a** HCOOH, **b** CH_3_COOH, **c** CH_3_OH and **d** C_2_H_5_OH at 0 V potential. **e** Relative selectivity evaluation based on DFT calculations on reaction free energies of all oxygenate products on the Cu-pyrrolic-N_4_ site of Cu-SA/NPC. (This figure has been added into the revised manuscript as new Fig. 6.)

**Action:** To address the reviewer’s question and incorporate the new calculation results, the following discussions were added into the revised manuscript on **Pages 17-19** as:

“*As observed in* ***Fig. 3b****, other oxygenates such as HCOOH, CH_3_COOH, CH_3_OH and C_2_H_5_OH were also detected at different potentials. The energetic pathways for the formation of these oxygenates on the Cu-pyrrolic-N_4_ site of Cu-SA/NPC were examined by DFT calculations and the results were illustrated in* ***Fig. 6a****-****6d****. Optimized structures of all intermediates were provided in* ***Fig. S24*** *to* ***S27****, with the free energy change for each elementary step involved given in* ***Table S7****. The relative selectivity to acetone versus these oxygenates was evaluated based on DFT results at 0 and -0.36 V. For acetone, the rate-determining step was CO_2_ reduction to COOH*, which had a free energy change of 1.42 eV at 0 V potential. For HCOOH formation, the rate-limiting step was CO_2_ reduction to HCOO* with a ΔG of 2.42 eV, determining the selectivity to formic acid and making it difficult to form at lower potentials. However, the formation of CH_3_COOH, CH_3_OH and C_2_H_5_OH all went through a CO* intermediate via CO_2_🡪 COOH* 🡪 CO*, similar to that for acetone formation. Therefore, the selectivity determining step for acetone versus other oxygenates should appear after CO* formation, which was found to be the COCOCH_3_* reduction to COHCOCH_3_* step with a ΔG of 0.32 eV for acetone formation, the CHOH* reduction to CH*+H_2_O(aq) step with a ΔG of 1.12 eV associated with CH_3_COOH formation, the CH_2_O* reduction to CH_2_OH* step with a ΔG of 0.17 eV for CH_3_OH formation and the CHOCH_2_OH* reduction to CHOHCH_2_OH* step with a ΔG of 0.79 eV in regard to C_2_H_5_OH formation, at 0 V potential. Since these steps were all electrochemical reactions, the applied potential could drive these steps occurring more facilely. The relative selectivities predicted for these oxygenate products based on reaction free energy calculations at varied potentials (0 and -0.36 V) were illustrated in* ***Fig. 6e****, which was generally consistent with the experimental results shown in* ***Fig. 3b****. At lower potentials (≤ -0.36 V), the HCOOH and CH_3_COOH could not be produced due to higher uphill ΔG values; the formation of CH_3_COCH_3_ and CH_3_OH was relatively fast because the selectivity determining step became more energetically favorable with the applied potentials; the C_2_H_5_OH could be produced but the selectivity should be lower than that of CH_3_COCH_3_ and CH_3_OH due to a moderate uphill free energy change of the selectivity determining step when the potential was applied. The higher selectivity toward acetone formation from CO_2_ reduction on the Cu-SA/NPC catalyst should originate from the unique Cu-pyrrolic-N_4_ active site in stabilizing reaction intermediates involved in acetone production as well as facilitating the C-C coupling reactions involved in CO_2_ reduction to acetone. As shown in* ***Fig. 5a****, the two C-C coupling steps were largely downhill in free energy change (-1.22 and -0.92 eV, respectively, for CO*-CO* and CO*-COCH_3_* coupling) and the formation of reaction intermediates was energetically quite favorable (except for the first CO_2_ reduction to COOH* step) due to the synergy of Cu-N coordination and interaction with intermediate species for acetone formation on the Cu-pyrrolic-N_4_ site of Cu-SA/NPC.*”

*4. The C-C coupling and acetone formation pathway considered here are just one of several possible pathway. For example, only the CO-CO coupling is considered here in C-C coupling, while CO-CHO and CO-COH are also possible mechanism. Justification of the reaction pathway considered here, or other pathways should be considered to find* *the most plausible pathway.*
 **Response:** We have considered other possible pathways for C-C coupling including the CO*-CHO* and CO*-COH* coupling routes. We identified that the direct coupling of two CO* species was energetically most favorable, which was significantly downhill in free energy change (-1.23 eV), as shown in **Fig. R8** below. The formation of COH* from CO* reduction was 0.98 eV uphill in free energy change and thus subsequent CO*-COH* coupling was not a preferred path albeit this C-C coupling step was substantially exothermic. CO* reduction to CHO* was less favorable comparing to CO*-CO* coupling and the COCHO* intermediate was much less stable than COCOH*. Therefore, the pathway going through the CO*-CO* coupling followed by COCO* reduction to a COCOH* intermediate should be the preferred mechanism for acetone production from CO_2_ reduction on the Cu-pyrrolic-N_4_ site of Cu-SA/NPC.

**Fig. R8** Free energy diagrams calculated for CO_2_ reduction through the CO*-CO* coupling, CO*-CHO* coupling, and CO*-COH* coupling pathways. The optimized structures of key CHO*, COH* and COCO* intermediates were included in the figure. (This figure has been added into the Supporting Information as new Fig. S23.)

**Action:** To answer this question, the following descriptions were added into the revised manuscript on **Page 17** as:

“*Other possible C-C coupling pathways were also examined on the Cu-pyrrolic-N_4_ site of Cu-SA/NPC, including CO*-CHO* and CO*-COH* coupling, and were compared with the CO*-CO* coupling route. As illustrated in* ***Fig. S23****, the formation of COH* from CO* reduction was 0.98 eV endothermic in free energy change and thus subsequent CO*-COH* coupling was not a preferred path despite that this C-C coupling was substantially exothermic (-2.27 eV). Meanwhile, the CO* reduction to CHO* (ΔG of -0.50 eV) was less favorable as compared to the direct coupling of two CO* species with a ΔG of -1.23 eV. Moreover, the formed COCHO* species was much less stable than the COCOH* species (****Fig. S23****). Therefore, the route going through CO*-CO* coupling followed by the formation of a COCOH* intermediate should be the most plausible pathway for acetone production from CO_2_ reduction.*”

*5. The production rates of HCOOH and CH_3_COOH on N-doped porous carbon (NPC) are 200~300 μg h^-1^ at -0.76 V (Fig. S8), while those on Cu-SA/NPC are less than 150 μg h^-1^ at same potential (Fig. 3b). This result indicates that HCOOH and CH_3_COOH produced from NPC may further reduced to acetone on Cu-SA/NPC.*

**Response:** The selectivity of products produced from CO_2_ reduction was related to the binding energy of intermediates on catalyst surfaces, which could be influenced by the different surface and electronic properties of catalysts. In this work, the NPC and Cu-SA/NPC possessed different surface structure and electronic properties, resulting in different selectivities for CO_2_ reduction. To identify whether HCOOH and CH_3_COOH could be further reduced to acetone on Cu-SA/NPC, the reduction experiments of HCOOH and CH_3_COOH were conducted on the Cu-SA/NPC cathode. The 0.1 M KHCO_3_ electrolyte with additional HCOOH (20 mg L^-1^) or CH_3_COOH (20 mg L^-1^) was used as electrolyte. During the reduction process, CO_2_ gas was absent. The reduction experiments were conducted at the potential of -0.76 V vs. RHE. After 2 h of reduction, the samples were analyzed by 1H NMR. As shown in **Fig. R9** below, no peak associated with acetone was detected from 1H NMR spectra. These results indicated that the formed HCOOH and CH_3_COOH products could not be further reduced to acetone on Cu-SA/NPC.

**Fig. R9** ^1^H NMR spectra of HCOOH and CH_3_COOH reduction on Cu-SA/NPC catalysts. (This figure has been added into the revised Supporting Information as new Fig. S8.)

**Action:** The following sentence was added in the revised manuscript on **Page 9** as:

*“**As shown in* ***Fig. S8****, no peak associated with acetone was detected from ^1^H NMR spectra after reducing HCOOH or CH_3_COOH in 0.1 M KHCO_3_ electrolyte, suggesting that the formed HCOOH and CH_3_COOH products could not be further reduced to acetone on Cu-SA/NPC.”*

*6. It is unclear that the adsorbents are bound to Cu or N atom in Cu-SA/NPC (Fig. 5d). Further clarification and top-view of geometries should be provided.*

**Response:** We thank the reviewer for this suggestion.

**Action:** We revised **Fig. 5d** (now **Fig. 5b** in the revised manuscript) by providing both top and side views of each structure for clarity. The entire **Fig. 5** was reorganized for improvement, as shown below.

**Fig. 5** **a** Free energy diagrams calculated at a potential of -0.36 V for CO_2_ reduction to CH_3_COCH_3_ on Cu-pyridinic-N_4_ and Cu-pyrrolic-N_4_ sites of Cu-SA/NPC (the computational models were included in the figure). **b** Optimized structures of all reaction intermediates involved in the pathways of CO_2_ reduction on the Cu-pyrrolic-N_4_ site (grey: C of catalyst; black: C of adsorbate; red: O; orange: Cu; blue: N; white: H).

**~~---------------------------------------------------------------------------------------------------------------------~~**

**Reviewer #3 (Remarks to the Author):**

*The authors have presented an interesting Cu single-atom catalyst with Cu atoms coordinated on N-doped carbon derived from ZIF-8. The Cu-SAC was found to be highly active for producing acetone. Acetone is not a common product in CO_2_ reduction, but isotope labeling has proven the production of acetone. Several questions remain elusive:*

**Response:** We thank the reviewer for considering “the authors have presented an interesting Cu single-atom catalyst with Cu atoms coordinated on N-doped carbon for highly selective production of acetone”. To improve and strengthen the manuscript, we performed additional experiments and DFT calculations and made revisions by incorporating all comments and suggestions from the reviewer, as detailed below.

*1. The author needs to provide explanation on the absence of gaseous carbon-based product, such as CO, CH_4_ and C_2_H_4_. *CO is in the proposed pathway and it’s unusual to see no CO is released at all from a Cu-based catalyst.*

**Response:** We thank the review for this comment. The gas phase products were collected into a multilayer gas sampling bag and identified by gas chromatography (Shimadzu, GC-14C) equipped with a thermal conductivity detector (TCD). CO was detected as a gas phase product and the Faradaic efficiency was about 9.5~17.4% at the studied potential range, as shown in **Fig. R10** below.

**Action:** The following sentence was added into the revised manuscript on **Page 8** as:

“*The detected liquid products on Cu-SA/NPC were formic acid (HCOOH), acetic acid (CH_3_COOH), methanol (CH_3_OH), ethanol (C_2_H_5_OH) and acetone (**CH_3_COCH_3_), while the gaseous products were H_2_ and CO.*”

**Fig. R10** Gaseous CO product on Cu-SA/NPC at different applied potentials.

*2. What’s the active site? It is hard to be convinced that a single Cu site could enable C-C coupling and even stabilize C3 intermediates. CO coverage on a continuous Cu surface is thought to be highly important for C-C coupling step, which requires neighboring *CO adsorbates (**DOI: 10.1021/jacs.7b03300,* *DOI: 10.1038/s41467-018-07970-9)*

**Response:** As evidenced from the EXAFS fitting results (**Fig. 2c and Table S2**), the Cu atom was coordinated with N atoms with a Cu-N coordination number of 3.8 ± 0.2. Based on the combined HAADF-STEM and EXAFS results, it was evidenced that the Cu species was atomically dispersed and was four-fold coordinated with N atoms in Cu-SA/NPC. The DFT calculations by comparing different types of Cu-N and N=O sites, including pyridinic and pyrrolic N, revealed that the main active sites for acetone production from CO_2_ reduction on the Cu-SA/NPC catalyst were the assemble of the Cu-pyrrolic-N_4_ sites, in which the Cu single atom was coordinated to four pyrrolic N atoms, as shown in **Fig. 5a**. For CO_2_ reduction to acetone, the formation of reaction intermediates required the synergy between Cu and N sites (see **Fig. 5b**), resulting in quite facile C-C coupling toward C_2_ and C_3_ species formation. In addition, the additional experimental results (**Figs. R11**, **R12** and **Table R1** below) showed that the content of Cu dispersed into the NPC catalyst was relatively low, and thus the possibility of forming consecutive Cu-Cu bond was small. In the literature, it was evidenced that both Cu metal ^41-43^ and the N-doped carbon catalysts ^44-46^ were active to reduce CO_2_ to C_2_ products via C-C coupling. Our DFT calculations clearly demonstrated that CO_2_ reduction to acetone was energetically quite favorable on the assemble of the Cu-pyrrolic-N_4_ sites of the Cu-SA/NPC catalyst.

**Fig. R11** Production rate and Faradic efficiency of acetone production on Cu-SA/NPC_0.5_, Cu-SA/NPC, Cu-SA/NPC_2_, and Cu-SA/NPC_3_ at potential of -0.76 V vs. RHE. (This figure has been added into the revised Supporting Information as new Fig. S15.)

**Fig. R12** XPS of Cu-SA/NPC_0.5_, Cu-SA/NPC, Cu-SA/NPC_2_, and Cu-SA/NPC_3_. (This figure has been added into the revised Supporting Information as new Fig. S16.)

**Table R1**. The content of Cu and N of prepared catalysts *^a^*. (This table has been added into the revised Supporting Information as new Table S4.)

| Sample | Cu_Total_  (at.%) | N_Total_  (at.%) | Pyridinic N (at.%) | Pyrrolic N  (at.%) | Graphitic N (at.%) | Cu-N  (at.%) | N-O  (at.%) |
| --- | --- | --- | --- | --- | --- | --- | --- |
| NPC | -- | 7.8 | 2.7 | 2.2 | 1.8 | -- | 1.1 |
| Cu-SA/NPC_0.5_ | 0.2 | 8.3 | 2.6 | 1.5 | 2.0 | 1.5 | 0.7 |
| Cu-SA/NPC | 0.2 | 8.4 | 1.5 | 1.9 | 2.4 | 1.5 | 1.1 |
| Cu-SA/NPC_2_ | 0.3 | 6.5 | 1.3 | 1.5 | 1.6 | 1.5 | 0.6 |
| Cu-SA/NPC_3_ | 0.3 | 6.4 | 1.3 | 1.3 | 1.6 | 1.4 | 0.8 |
| Cu-SA/NPC_Ar_ | 0.2 | 8.2 | 2.6 | 1.4 | 2.0 | 1.6 | 0.6 |

*^a^* Catalysts with adding Cu content of 0, 0.16, 0.31, 0.62, 0.93 and 1.86 g were denoted as NPC, Cu-SA/NPC_0.5_, Cu-SA/NPC, Cu-SA/NPC_2_, Cu-SA/NPC_3_, respectively. Catalysts carbonized at Ar atmosphere was denoted as Cu-SA/NPC_Ar_.

**Action:** To address this question from the reviewer, the following sentences were added into the revised manuscript on **Page 21** as:

*“These results confirmed that the main active sites for acetone synthesis from CO_2_ reduction on the Cu-SA/NPC catalyst were the Cu-pyrrolic-N_4_, consistent with the structural prediction of Cu/N coordination environment from HAADF-STEM and EXAFS characterization. For CO_2_ reduction to acetone, the formation of reaction intermediates required the synergy between Cu and N sites (****see Fig. 5b****), leading to quite facile C-C coupling toward C_2_ and C_3_ species formation. In addition, the additional experimental results provided in* ***Table S4*** *revealed that the content of Cu dispersed into the NPC catalyst was relatively less, and thus the possibility of forming Cu clusters was small. In the literature, it was evidenced that not only Cu metal was beneficial for C-C coupling ^41-43^, but the N-doped carbon catalysts also could reduce CO_2_ to C_2_ products via C-C coupling ^44-46^. The DFT calculations in the current study clearly demonstrated that CO_2_ reduction to acetone was energetically quite favorable catalyzed by the Cu-pyrrolic-N_4_ site of Cu-SA/NPC due to the crucial Cu-N synergy.*”

*3. More control experiments are needed. Did the coordinated N-atoms participate in the reaction? Or the reaction require synergy between two adjacent Cu sites? Comparative studies of, such as NPC and Cu-SA/NPC with different Cu loadings should be done to study the role of different active sites.*

**Response:** As suggested by the reviewer, the Cu-SA/NPC catalysts with different Cu content were prepared. The Cu-SA/NPC in the original manuscript was synthesized with the Zn/Cu ratio of 10/1. The catalysts with Zn/Cu ratios of 20/1, 5/1, 10/3, and 5/3 were synthesized and denoted as Cu-SA/NPC_0.5_, Cu-SA/NPC_2_, Cu-SA/NPC_3_, and Cu-SA/NPC_6_, respectively. As shown in **Fig. R13** below, the Cu-SA/NPC_0.5_, Cu-SA/NPC_2_ and Cu-SA/NPC_3_ retained the rhombic dodecahedral morphology of pristine ZIF-8, and the size of prepared catalysts increased with increasing Cu content. The pristine rhombic dodecahedral morphology was destroyed when further increased the Cu content to a Zn/Cu ratio of 5/3 (Cu-SA/NPC_6_). Therefore, the CO_2_ electroreduction was conducted on Cu-SA/NPC_0.5_, Cu-SA/NPC, Cu-SA/NPC_2_ and Cu-SA/NPC_3_ at -0.76 V (see **Fig. R11** above), with a acetone production rate of 108.6, 336.1, 194.7 and 129.3 μg h^-1^, respectively. The Faradic efficiency of acetone generation followed the same trend as Cu-SA/NPC > Cu-SA/NPC_2_ > Cu-SA/NPC_3_ > Cu-SA/NPC_0.5_. According to the XPS results, the Cu content of Cu-SA/NPC_0.5_, Cu-SA/NPC, Cu-SA/NPC_2_ and Cu-SA/NPC_3_ catalysts increased from 0.1 to 0.3 % (**Table R1**). However, the N content also increased slightly from 8.3 to 8.4 % as the Zn/Cu ratio increased to 10/1, and the N content decreased to 6.4% with further increasing the Zn/Cu ratio to 10/3 (**Fig. R14** and **Table R1**). The trend in the change of N content with the doping content of Cu metal was similar to that reported in the literature (*ACS Catal. 2017, 7, 1655-1663*). Therefore, acetone production from CO_2_ on Cu-SA/NPC was not only related to the atomic Cu doping, but also related to the N content and species.

Furthermore, the DFT calculations of energetic pathways for acetone formation from CO_2_ reduction on different types of Cu-N and N=O sites, including pyridinic and pyrrolic N were conducted (**Fig. 5** and **Fig. R2**). The results revealed that the main active sites for acetone production from CO_2_ reduction on the Cu-SA/NPC catalyst were the assemble of the Cu-pyrrolic-N_4_ sites, in which the Cu single atom was coordinated to four pyrrolic N atoms, as shown in **Fig. 5** in the revised manuscript. The acetone production from CO_2_ reduction required the synergy between Cu-N sites in the Cu-SA/NPC catalyst.

**Action:** To address these questions, the following discussions were added in the Sections “**Methods**”, “**Electrochemical reduction of CO_2_ on Cu-SA/NPC**” and “**Pyridinic-N=O and pyrrolic-N=O sites**” on **Page 23**, **Pages 11-12** and **Pages 20-21** as:

**Page 23** “*The catalysts with different content of Cu (0.16, 0.62, 0.93 and 1.86 g Cu with Zn/Cu ratios of 20/1, 5/1, 10/3 and 5/3, respectively) were also synthesized and the obtained catalysts were denoted as Cu-SA/NPC_0.5_, Cu-SA/NPC_2_, Cu-SA/NPC_3_, and Cu-SA/NPC_6_, respectively.*”

**Pages 11-12** “*The Cu-SA/NPC catalysts with different Cu content were prepared. The Cu-SA/NPC in the original manuscript was synthesized with the Zn/Cu ratio of 10/1. The catalysts with Zn/Cu ratios of 20/1, 5/1, 10/3, and 5/3 were synthesized and denoted as Cu-SA/NPC_0.5_, Cu-SA/NPC_2_, Cu-SA/NPC_3_, and Cu-SA/NPC_6_, respectively. As shown in* ***Fig. S14****, the Cu-SA/NPC_0.5_, Cu-SA/NPC_2_ and Cu-SA/NPC_3_ retained the rhombic dodecahedral morphology of pristine ZIF-8, and the size of prepared catalysts increased with increasing Cu content. The pristine rhombic dodecahedral morphology was destroyed when further increased the Cu content to a Zn/Cu ratio of 5/3 (Cu-SA/NPC_6_). Therefore, the CO_2_ electroreduction was conducted on Cu-SA/NPC_0.5_, Cu-SA/NPC, Cu-SA/NPC_2_ and Cu-SA/NPC_3_ at -0.76 V (****Fig. S15****), with a acetone production rate of 108.6, 336.1, 194.7 and 129.3 μg h^-1^, respectively. The Faradic efficiency of acetone generation followed the same trend as Cu-SA/NPC > Cu-SA/NPC_2_ > Cu-SA/NPC_3_ > Cu-SA/NPC_0.5_. According to the XPS results, the Cu content of Cu-SA/NPC_0.5_, Cu-SA/NPC, Cu-SA/NPC_2_ and Cu-SA/NPC_3_ catalysts increased from 0.1 to 0.3 % (****Table S4****). However, the N content also increased slightly from 8.3 to 8.4% as the Zn/Cu ratio increased to 10/1, and the N content decreased to 6.4% with further increasing the Zn/Cu ratio to 10/3 (****Figs. S16****,* ***S17*** *and* ***Table S4****). The trend in the change of N content with the doping content of Cu metal was similar to that reported in the literature ^33^. Therefore, acetone production from CO_2_ on Cu-SA/NPC was not only related to the atomic Cu doping, but also related to the N content and species.*”

**Pages 20-21 “*Pyridinic-N=O and pyrrolic-N=O sites****. As shown in* ***Table S4****, the content of graphitic N and Cu-N was similar for the two compared catalysts. However, the content of pyridinic N, pyrrolic N and oxidized N was different, which may result in the different activities of the two compared catalysts. The experimental results on N-doped porous carbon catalysts without adding Cu showed no acetone formation from CO_2_ reduction, which suggested that the oxidized N would not be the active site for acetone production on the Cu-SA/NPC catalyst. To further confirm the main active sites for the selective acetone formation on Cu-SA/NPC, DFT calculations of energetic pathways for CO_2_ reduction to acetate on two types of oxidized N sites, including pyridinic- and pyrrolic-N=O sites, were further performed. The results showed that the pyridinic-N=O site was not active for CO_2_ reduction to acetone since several steps involved in the conversion were highly uphill in ΔG, such as CO*-CO* coupling and CO*-COCH_3_* coupling steps with ΔG values of 1.05 and 1.62 eV, respectively, at 0 V (see* ***Fig. S28a****). On the pyrrolic-N=O site, although the CO_2_ reduction to COOH* step became facile comparing to that occurred on the Cu-pyrrolic-N_4_ site, some elementary steps involved in acetone formation had large ΔG values, such as COCH_2_* reduction to COCH_3_* and CO*-COCH_3_* coupling steps with ΔG of ~0.8 eV at 0 V (see* ***Fig. S28b****). Since the C-C coupling reaction could not be facilitated by the applied potential, the acetone formation should not prefer to occur on the pyrrolic-N=O site. It was worth noting that only the reduction of CO_2_ to COOH* involved the N=O site to form a N-O-COOH* intermediate, but other steps mainly occurred on adjacent C-N sites or C-N****^…^****N-C bridge sites with less O atom interaction on the two types of oxidized N sites (all intermediates involved in acetone formation were provided in* ***Fig. S29*** *and* ***S30****). Therefore, the oxidized N sites were not the active sites for acetone formation.*

*These results confirmed that the main active sites for acetone synthesis from CO_2_ reduction on the Cu-SA/NPC catalyst were the Cu-pyrrolic-N_4_, consistent with the structural prediction of Cu/N coordination environment from HAADF-STEM and EXAFS characterization. For CO_2_ reduction to acetone, the formation of reaction intermediates required the synergy between Cu and N sites (****see Fig. 5b****), leading to quite facile C-C coupling toward C_2_ and C_3_ species formation. In addition, the additional experimental results provided in* ***Table S4*** *revealed that the content of Cu dispersed into the NPC catalyst was relatively less, and thus the possibility of forming Cu clusters was small. In the literature, it was evidenced that not only Cu metal was beneficial for C-C coupling^41-43^, but the N-doped carbon catalysts also could reduce CO_2_ to C_2_ products via C-C coupling^44-46^. The DFT calculations in the current study clearly demonstrated that CO_2_ reduction to acetone was energetically quite favorable catalyzed by the Cu-pyrrolic-N_4_ site of Cu-SA/NPC due to the crucial Cu-N synergy.*

**Fig. R13** SEM of **a** Cu-SA/NPC_0.5_, **b** Cu-SA/NPC_2_, **c** Cu-SA/NPC_3_, and **d** Cu-SA/NPC_6_. (This figure has been added into the revised Supporting Information as new Fig. S14.)

**Fig. R14** XPS 1N spectra of **a** Cu-SA/NPC_0.5_, **b** Cu-SA/NPC_2_ and **c** Cu-SA/NPC_3_. (This figure has been added into the Supporting Information as new Fig. S17.)

**Fig. R2**. Free energy diagrams calculated at potentials of 0 and -0.36 V for CO_2_ reduction to CH_3_COCH_3_ on **a** pyridinic-N=O and **b** pyrrolic-N=O sites. The two computational models are included in the figure. (This figure has been added into the revised Supporting Information as new Fig. S28.)

*4. For the mechanism discussed in Figure 5, did the authors compare competitive pathways? Can a C1 or C2 product be more favorable than acetone?*

**Response:** In the revised manuscript, other possible pathways were discussed. The mechanism proposed in the manuscript for acetone production from CO_2_ reduction was verified to be the energetically most favorable pathway (see **Fig. R8** below). Moreover, the energetic pathways for the formation of other experimentally observed C_1_ and C_2_ oxygenates (HCOOH, CH_3_COOH, CH_3_OH and C_2_H_5_OH) at varied potentials were examined by performing additional DFT calculations (see **Fig. R7** below). The relative selectivities among these C_1_, C_2_ and C_3_ oxygenate products as well as the origin of selective acetone formation were further discussed in the revised manuscript (see “**Action**” below).

**Fig. R8** Free energy diagrams calculated for CO_2_ reduction through the CO*-CO* coupling, CO*-CHO* coupling, and CO*-COH* coupling pathways. The optimized structures of key CHO*, COH* and COCO* intermediates were included in the figure. (This figure has been added into the Supporting Information as new Fig. S23.)

**Fig. R7** Free energy diagrams calculated for CO_2_ reduction to **a** HCOOH, **b** CH_3_COOH, **c** CH_3_OH and **d** C_2_H_5_OH at 0 V potential. **e** Relative selectivity evaluation based on DFT calculations on reaction free energies of all oxygenate products on the Cu-pyrrolic-N_4_ site of Cu-SA/NPC. (This figure has been added into the revised manuscript as new Fig. 6.)

**Action:** To address these questions and incorporate the additional DFT results, the following discussions were added into the revised manuscript on **Pages 17** and **17-19** as:

**Page 17** “*Other possible C-C coupling pathways were also examined on the Cu-pyrrolic-N_4_ site of Cu-SA/NPC, including CO*-CHO* and CO*-COH* coupling, and were compared with the CO*-CO* coupling route. As illustrated in* ***Fig. S23****, the formation of COH* from CO* reduction was 0.98 eV endothermic in free energy change and thus subsequent CO*-COH* coupling was not a preferred path despite that this C-C coupling was substantially exothermic (-2.27 eV). Meanwhile, the CO* reduction to CHO* (ΔG of -0.50 eV) was less favorable as compared to the direct coupling of two CO* species with a ΔG of -1.23 eV. Moreover, the formed COCHO* species was much less stable than the COCOH* species (****Fig. S23****). Therefore, the route going through CO*-CO* coupling followed by the formation of a COCOH* intermediate should be the most plausible pathway for acetone production from CO_2_ reduction.*”

**Pages 17-19** “*As observed in* ***Fig. 3b****, other oxygenates such as HCOOH, CH_3_COOH, CH_3_OH and C_2_H_5_OH were also detected at different potentials. The energetic pathways for the formation of these oxygenates on the Cu-pyrrolic-N_4_ site of Cu-SA/NPC were examined by DFT calculations and the results were illustrated in* ***Fig. 6a****-****6d****. Optimized structures of all intermediates were provided in* ***Fig. S24*** *to* ***S27****, with the free energy change for each elementary step involved given in* ***Table S7****. The relative selectivity to acetone versus these oxygenates was evaluated based on DFT results at 0 and -0.36 V. For acetone, the rate-determining step was CO_2_ reduction to COOH*, which had a free energy change of 1.42 eV at 0 V potential. For HCOOH formation, the rate-limiting step was CO_2_ reduction to HCOO* with a ΔG of 2.42 eV, determining the selectivity to formic acid and making it difficult to form at lower potentials. However, the formation of CH_3_COOH, CH_3_OH and C_2_H_5_OH all went through a CO* intermediate via CO_2_🡪 COOH* 🡪 CO*, similar to that for acetone formation. Therefore, the selectivity determining step for acetone versus other oxygenates should appear after CO* formation, which was found to be the COCOCH_3_* reduction to COHCOCH_3_* step with a ΔG of 0.32 eV for acetone formation, the CHOH* reduction to CH*+H_2_O(aq) step with a ΔG of 1.12 eV associated with CH_3_COOH formation, the CH_2_O* reduction to CH_2_OH* step with a ΔG of 0.17 eV for CH_3_OH formation and the CHOCH_2_OH* reduction to CHOHCH_2_OH* step with a ΔG of 0.79 eV in regard to C_2_H_5_OH formation, at 0 V potential. Since these steps were all electrochemical reactions, the applied potential could drive these steps occurring more facilely. The relative selectivities predicted for these oxygenate products based on reaction free energy calculations at varied potentials (0 and -0.36 V) were illustrated in* ***Fig. 6e****, which was generally consistent with the experimental results shown in* ***Fig. 3b****. At lower potentials (≤ -0.36 V), the HCOOH and CH_3_COOH could not be produced due to higher uphill ΔG values; the formation of CH_3_COCH_3_ and CH_3_OH was relatively fast because the selectivity determining step became more energetically favorable with the applied potentials; the C_2_H_5_OH could be produced but the selectivity should be lower than that of CH_3_COCH_3_ and CH_3_OH due to a moderate uphill free energy change of the selectivity determining step when the potential was applied. The higher selectivity toward acetone formation from CO_2_ reduction on the Cu-SA/NPC catalyst should originate from the unique Cu-pyrrolic-N_4_ active site in stabilizing reaction intermediates involved in acetone production as well as facilitating the C-C coupling reactions involved in CO_2_ reduction to acetone. As shown in* ***Fig. 5a****, the two C-C coupling steps were largely downhill in free energy change (-1.22 and -0.92 eV, respectively, for CO*-CO* and CO*-COCH_3_* coupling) and the formation of reaction intermediates was energetically quite favorable (except for the first CO_2_ reduction to COOH* step) due to the synergy of Cu-N coordination and interaction with intermediate species for acetone formation on the Cu-pyrrolic-N_4_ site of Cu-SA/NPC.*”

*5. Was the catalyst stable during the reaction? More characterizations and comparisons are needed for the catalysts before and after the measurements.*

**Response:** The post-reaction catalysts were characterized by SEM and HAADF-STEM characterizations. As shown in **Fig. R15** below, the post-reaction catalysts retained the rhombic dodecahedral structure and the Cu metal was still presented in atomic dimensions.

**Action:** To address this question and incorporate the new results, the following sentences were added into the revised manuscript on **Page 11** as:

“*Furthermore, the catalysts were characterized by SEM and HAADF-STEM (****Fig. S12****) after five sequential CO_2_ reduction. The rhombic dodecahedral morphology of the post-reaction catalysts was intact. The HAADF-STEM results showed that Cu remained atomically distributed in porous carbon. These results indicated that the Cu-SA/NPC catalyst showed good stability in CO_2_ electrochemical reduction.*”

**Fig. R15** Characterizations of post-catalysts. **a** SEM, **b, c** HAADF-STEM, **d-g** EDS images of post-catalysts by HAADF-STEM. (This figure has been added into the revised Supporting Information as new Fig. S12.)

*6. The discussion of HCO_3_^-^ participation in the reaction is somewhat elusive. Can the authors provide more insights into the connection to the MS spectra?*

**Response:** We apologized for missing detailed explanations of the role of HCO_3_^-^ in CO_2_ reduction. The bicarbonate anions were served as an electrolyte, which has been identified as providing pH buffer and proton donor in the literature. However, recent studied found that CO_2_ in equilibrium with bicarbonates played an essential role in the CO_2_ reduction reaction (e.g. *ACS Energy Lett. 2019, 4, 682-689*), indicating the existence of a dynamic equilibrium between CO_2_ partial pressure and HCO_3_^-^ concentration.

Lin *et al.* pointed out that CO_2_ could be converted to HCO_3_^-^ and used ^13^CO_2_ exchange experiment to estimate the rate of CO_2_ mass transport. In their work, the ^13^CO_2_ gas was used to fill the headspace of the vial with 0.1 M NaH^12^CO_3_ electrolyte under stirring. The ^13^CO_2_/^12^CO_2_ ratio in the headspace was measured by online mass spectrometry. The specific mass transport coefficient was about 0.0015 s^-1^ by measuring the rate of ^13^CO_2_ transfer between gas and liquid phases (*J. Am. Chem. Soc. 2019, 141, 17875-17883*).

The methanol (C_1_ product) and ethanol (C_2_ product) yields from CO_2_ reduction in ^13^CO_2_ or H^13^CO_3_^-^ were analyzed by GC-MS. As shown in **Fig. R6** below, both the *m/z*=31 and *m/z*=32, characteristic peaks of CH_3_OH and ^13^C-labeled CH_3_OH, respectively, were observed in the spectra of ^13^CO_2_ or H^13^CO_3_, indicating that HCO_3_^-^ was involved in CH_3_OH formation. The peaks at *m/z*=28 and *m/z*=29, as characteristic peaks of ^13^C-labeled C_2_H_5_OH, were observed in ^13^CO_2_ reduction, while *m/z*=27, a characteristic peak of C_2_H_5_OH, was also detected. In the spectra of H^13^CO_3_^-^, the peaks at *m/z*=28 was also detected. These results demonstrated that HCO_3_^-^ anions participated in the CO_2_ reduction reaction to produce acetone.

**Action:** To address this question from the reviewer and incorporated the new results, the following text was added into the revised manuscript in the Section “**Electrochemical reduction of CO_2_ on Cu-SA/NPC”** on **Page 9** as:

“*As shown in* ***Fig. S11****, the characteristic peaks of CH_3_OH (m/z=31 and m/z=32) and C_2_H_5_OH (m/z=27, m/z=28 and m/z=29) were detected in ^13^CO_2_ and H^13^CO_3_. These results were consistent with those reported in the literature ^29,30^. According to the previous studies ^29,30^, there should be a dynamic equilibrium between CO_2_ partial pressure and HCO_3_^-^ concentration and the CO_2_ in equilibrium with HCO_3_^-^ was the source for CO_2_ reduction reaction.”*

**Fig. R6** GC-MS spectra of **a** CH_3_OH and **b** C_2_H_5_OH production form ^13^CO_2_ electrochemical reduction, **c** CH_3_OH and **d** C_2_H_5_OH production from CO_2_ electrochemical reduction in H^13^CO_3_^-^. (This figure has been added into the revised Supporting Information as new Fig. S11.)

**Reviewer comments, second version:**

Reviewer #1 (Remarks to the Author):

In this version, the authors included a more detailed analysis of the XPS and additional DFT study, however, in my opinion the manuscript presents still inconsistencies, which need to be addressed, therefore I do not recommend publication of the manuscript at the current state. The fitting of the N1s in figure R1 requires improvements and is not consistently applied to the two figures in terms of FWHM of the peaks and this could lead to false conclusions, therefore it deserves further considerations from the authors:
a) The envelope peak relative to the Npyr goes outside the spectrum lineshape, which suggest that the fitting is not correct. This must be changed.
b) Particularly for Fig R1d the background is not taken correctly which leads to underestimate the high BE peaks;
c) The FWHM and BE for the NO species and pyrrolic N do not appear to be the same in every spectra and I do not see any reasons for doing that.
It is of paramount importance that the fitting is the same for all N1s core levels spectra.

In the current review the authors focus on evaluating the relevance of the oxidized species, apparently following up on my previous comments. While this was actually not my initial point, but specifically I referred to the fact that pyridine oxide appears at the same binding energy of pyrrolic N and quaternary N, while what they indicate as N-O are nitrous moieties, which are certainly not catalytically active. I still see a lack of a cohesive and consistent interpretation of DFT and XPS analysis. I will try to be clearer now:
The authors suggest that “In view of CO2 reduction measurements and XPS results, the enhanced CO2 reduction activity and CH3COCH3 selectivity of Cu-SA/NPC should be attributed to the presence of single atom Cu coordinated with more pyrrolic N that served as active sites for CO2 reduction. “
However, the Cu-N species are similar for the two catalysts (synthetized in air and in Ar) whereas only the amount of uncoordinated pyrrolic N is different.
This seems a contradiction with their conclusions.
I would advise that the authors clearly describe the reactive ensemble and identify where the individual species composing the reactive ensemble contribute to the XPS spectra.
However, before doing that the fitting of the N1s must be improved and applied consistently.

Further clarifications are: Would the Cu-Pyrrol N1s component fall at a different BE than the Cu-Pyr? What species are they considering as Cu-N is that XPS fitting?
The impact of Zn was not discussed but it certainly has an impact of the performance. Can the authors comment on that?


all Points were satisfactory answered except point 2 and 3.
In my opinion the authors did not characterize very well the catalysts, neglecting the role of Zn used during the synthesis, which might be involved in the reaction mechanism. Their model on a co-participation of Cu single atoms and N species to bind CO2 and form C-C bonds is based on previously reported contributions, but no evidence are provided here that this is happening.
Other report have identified similar sites as active species however selectivity patterns are different. Therefore I do not think that this manuscript at the current state is adding more value to the knowledge that we have on the reaction mechanism. I believe that the authors should clearly answer this question.


Reviewer #2 (Remarks to the Author):

The authors have addressed all my previous comments properly, and I would recommend its publication.

**Author rebuttal, second version:**

*In this version, the authors included a more detailed analysis of the XPS and additional DFT study, however, in my opinion the manuscript presents still inconsistencies, which need to be addressed, therefore I do not recommend publication of the manuscript at the current state.* *The fitting of the N1s in figure R1 requires improvements and is not consistently applied to the two figures in terms of FWHM of the peaks and this could lead to false conclusions, therefore it deserves further considerations from the authors:
a) The envelope peak relative to the Npyr goes outside the spectrum lineshape, which suggest that the fitting is not correct. This must be changed.
b) Particularly for Fig R1d the background is not taken correctly which leads to underestimate the high BE peaks;
c) The FWHM and BE for the NO species and pyrrolic N do not appear to be the same in every spectra and I do not see any reasons for doing that.
It is of paramount importance that the fitting is the same for all N1s core levels spectra.

In the current review the authors focus on evaluating the relevance of the oxidized species, apparently following up on my previous comments. While this was actually not my initial point, but specifically I referred to the fact that pyridine oxide appears at the same binding energy of pyrrolic N and quaternary N, while what they indicate as N-O are nitrous moieties, which are certainly not catalytically active. I still see a lack of a cohesive and consistent interpretation of DFT and XPS analysis. I will try to be clearer now:
The authors suggest that “In view of CO_2_ reduction measurements and XPS results, the enhanced CO_2_ reduction activity and CH_3_COCH_3_ selectivity of Cu-SA/NPC should be attributed to the presence of single atom Cu coordinated with more pyrrolic N that served as active sites for CO_2_ reduction. “
However, the Cu-N species are similar for the two catalysts (synthetized in air and in Ar) whereas only the amount of uncoordinated pyrrolic N is different.
This seems a contradiction with their conclusions.
I would advise that the authors clearly describe the reactive ensemble and identify where the individual species composing the reactive ensemble contribute to the XPS spectra.
However, before doing that the fitting of the N1s must be improved and applied consistently.

Further clarifications are: Would the Cu-Pyrrol N1s component fall at a different BE than the Cu-Pyr? What species are they considering as Cu-N is that XPS fitting?
The impact of Zn was not discussed but it certainly has an impact of the performance. Can the authors comment on that?

all Points were satisfactory answered except point 2 and 3.
In my opinion the authors did not characterize very well the catalysts, neglecting the role of Zn used during the synthesis, which might be involved in the reaction mechanism. Their model on a co-participation of Cu single atoms and N species to bind CO_2_ and form C-C bonds is based on previously reported contributions, but no evidence are provided here that this is happening.
Other report have identified similar sites as active species however selectivity patterns are different. Therefore I do not think that this manuscript at the current state is adding more value to the knowledge that we have on the reaction mechanism. I believe that the authors should clearly answer this question.*

**Response:** Based on the reviewer’s comments and suggestions, we divided them into three questions: 1. The fitting of the N1s requires improvement; 2. The impact of Zn on CO_2_ electroreduction; and 3. The active sites for CO_2_ reduction. As described below, we have performed additional data analysis and DFT calculations to address all the questions.

***Question #1.*** *The fitting of the N1s requires improvement.*

**Response:** Based on Reviewer’s suggestion, the fitting of N 1s XPS was improved. The XPSPEAK41 software was used to analyze the obtained XPS spectra. Component fitting for N element was based on Gaussian-Lorentzian product function with a 20% Lorentzian-Gaussian value using Shirley background. The C 1s at 284.5 eV was used as the reference for charge correction. The percentage of different N species was determined by the ratio of peak area. For each type of N species, the binding energies (BE) and full width at half maximum (FWHM) were fixed at constant values and the detail parameters for fitting of N 1s spectra were summarized in **Table R1** below. The results were provided in **Figs. R1** and **R2**, and **Table R2** below. Both spectra exhibited five peaks with binding energies at 397.9, 398.9, 400.2, 401.0 and 402.7 eV, assigning to pyridinic N, metal Cu-N, pyrrolic N, graphitic N and oxidized N, respectively (**Fig. R1** and **Fig. R2**). As shown in **Table R2**, the content of graphitic N and metal Cu-N was similar, while that of pyridinic N, pyrrolic N, and oxidized N was different on Cu-SA/NPC and Cu-SA/NPC_Ar_. The content of pyridinic N and pyrrolic N on Cu-SA/NPC was 2.2% and 1.8%, respectively, while that on Cu-SA/NPC_Ar_ was 2.7% and 1.4%, respectively. This comparison indicated that the Cu-SA/NPC had a higher content of pyrrolic N than that of Cu-SA/NPC_Ar_, which may play an important role for the obtained high acetone selectivity on Cu-SA/NPC. According to the literature, the XPS N 1s spectra can be divided into pyridinic N, metal-N, pyrrolic N, graphitic N and oxidized N (*Adv. Funct. Mater*. 2015, 25, 872-882; *Environ. Energy Sci*. 2019, 12, 149-156). Therefore, the Cu-N is one kind of N species, neither Cu-pyridinic N nor Cu-pyrrolic N, in fitting of N 1s XPS spectra. The metal component, such as Cu, can be coordinated with either pyridinic N or pyrrolic N (*Nat. Catal*. 2018, 1, 339-348; *Nat. Mater*. 2015, 14, 937). The catalysts with more pyrrolic N can provide more pyrrolic N to form the Cu-N bond.

**Table R1.** Parameters for fitting of N 1s spectra

|  | Pyridinic N | Cu-N | Pyrrolic N | Graphitic N | N-O |
| --- | --- | --- | --- | --- | --- |
| BE (eV) | 397.9 | 398.9 | 400.2 | 401.0 | 402.7 |
| FWHM | 1.2 | 1.3 | 1.3 | 1.4 | 3.1 |

**Fig. R1** XPS N 1s spectra of **c** Cu-SA/NPC and **d** Cu-SA/NPC_Ar_. (This figure has been added into the revised manuscript as **Fig. 4c** and **d**.)

**Fig. R2** XPS N 1s spectra of **a** Cu-SA/NPC_0.5_, **b** Cu-SA/NPC_2_ and **c** Cu-SA/NPC_3_. (This figure has been added into the revised Supporting Information as **Fig. S17**.)

**Table R2.** The content of Cu and N of prepared catalysts *^a^.* (This table has been added into the revised Supporting Information as **Table S4**.)

| Sample | Cu_Total_  (at.%) | N_Total_  (at.%) | Pyridinic N (at.%) | Pyrrolic N  (at.%) | Graphitic N (at.%) | Cu-N  (at.%) | N-O  (at.%) |
| --- | --- | --- | --- | --- | --- | --- | --- |
| NPC | -- | 7.8 | 3.3 | 1.9 | 1.5 | -- | 1.1 |
| Cu-SA/NPC_0.5_ | 0.2 | 8.3 | 2.8 | 1.4 | 1.3 | 1.7 | 1.1 |
| Cu-SA/NPC | 0.2 | 8.4 | 2.2 | 1.8 | 1.4 | 1.8 | 1.2 |
| Cu-SA/NPC_2_ | 0.3 | 6.5 | 1.7 | 1.5 | 1.1 | 1.4 | 0.8 |
| Cu-SA/NPC_3_ | 0.3 | 6.4 | 1.7 | 1.3 | 1.1 | 1.3 | 1.0 |
| Cu-SA/NPC_Ar_ | 0.2 | 8.2 | 2.7 | 1.4 | 1.3 | 1.9 | 0.9 |

*^a^* Catalysts with adding Cu content of 0, 0.16, 0.31, 0.62, and 0.93 g were denoted as NPC, Cu-SA/NPC_0.5_, Cu-SA/NPC, Cu-SA/NPC_2_, Cu-SA/NPC_3_, respectively. Catalysts carbonized at Ar atmosphere was denoted as Cu-SA/NPC_Ar_.

**Action:** New discussions were added into the revised manuscript in the Section “**Electrochemical reduction of CO_2_ on Cu-SA/NPC**” on Pages 12-13. The method for fitting N 1s spectra was added into the revised Supporting Information in the Section “**XPS analysis**”.

**Pages 12-13** *“Both spectra exhibited five peaks with binding energies at 397.9, 398.9. 400.2, 401.0 and 402.7 eV, which were assigned to pyridinic N, metal Cu-N, pyrrolic N, graphite N and oxidized N (N-O), respectively ^35,36^. The content of graphite N and metal Cu-N was similar, while that of pyridinic N, pyrrolic N, and oxidized N (N-O) was different. As shown in* ***Table S4****, the NPC possessed the oxidized N content of 1.1%, similar to that of Cu-SA/NPC (1.2%). However, there was no acetone production from CO_2_ reduction on NPC without Cu (****Fig. S13****), suggesting the oxidized N alone might not play an important role in acetone production. As shown in* ***Table S4****, the content of pyridinic N and pyrrolic N on Cu-SA/NPC was 2.2% and 1.8%, respectively, while that on Cu-SA/NPC_Ar_ was 2.7% and 1.4%, respectively. This comparison indicated that the Cu-SA/NPC had a higher content of pyrrolic N than that of Cu-SA/NPC_Ar_, which might play an important role for acetone production on Cu-SA/NPC.”*

***Question #2.*** *The impact of Zn on CO_2_ electroreduction.*

**Response:** The Zn content was investigated by inductively coupled plasma atomic emission spectrometry and the results were presented in **Table S1 (**also provided below**)** in the original Supporting Information. The Zn content of Cu-SA/NPC and Cu-SA/NPC_Ar_ was measured to be 0.07 wt % and 0.06 wt %, respectively. As shown in **Table S1**, the Zn content of NPC was 0.90 wt %, which was 12 times higher than that of Cu-SA/NPC. However, the liquid products of CO_2_ reduction on NPC were HCOOH and CH_3_COOH (**Fig. S13**), demonstrating that Zn should not be active in the formation of CH_3_OH, C_2_H_5_OH and CH_3_COCH_3_. Moreover, Cu-SA/NPC and Cu-SA/NPC_Ar_ possessed a similar Zn content, but the selectivety of CH_3_OH, C_2_H_5_OH and CH_3_COCH_3_ production on these two catalysts was different, also indicating that Zn should not be responsible for the formation of CH_3_OH, C_2_H_5_OH and CH_3_COCH_3_. According to the literature, such low level of Zn on the catalysts could not combine with Cu nor change the selectivity toward CO_2_ reduction (*Angew. Chem. Int. Ed.* 2019, 58, 15098-15103). In this work, the Cu atom was identified to be coordinated with N atom as measured by the EXAFS characterization, demonstrating that Zn was not coordinated with Cu. Therefore, the trace amount of Zn impurity should have a negligible effect on acetone production from CO_2_ reduction on Cu-SA/NPC. The trace amount of Zn impurity was also reported with a negligible effect for other catalytic reactions (e.g. *ACS Catal..* 2018, 8, 1186-1191; *J. Mater. Chem. A,* 2019, 7, 26358-26363; *Materials Research Bulletin* 2020, 123, 110708).

In order to confirm the impact of Zn on acetone production from CO_2_ reduction in the current work, we performed additional DFT calculations on two types of catalyst models including Zn-pyrrolic-N_4_ and (Cu-Zn)-pyrrolic-N_3_ sites (see **Fig. R3** below). The Gibbs free energy change for several key elementary steps were calculated including CO_2_ reduction to COOH* and then to CO*, together with two non-electrochemical C-C coupling reactions, and compared with that obtained on Cu-pyrrolic-N_4_. As shown in **Fig. R3**, on Cu-pyrrolic-N_4_ site without Zn, the first eletroreduction step of CO_2_ to COOH* had a ΔG value of 1.42 eV, and was identified to be the rate-limiting step for acetone formation according to **Fig. 5** in the manuscript. The two C-C coupling reactions of CO*+CO* and CO*+COCH_3_* were energetically exothermic with ΔG values of -1.23 and -0.92 eV, respectively, indicating a facile formation of acetone from CO_2_ reduction on Cu-pyrrolic-N_4_. For comparison, on Zn-pyrrolic-N_4_ without Cu, the first electroreduction step of CO_2_ to COOH* was energetically more endothermic with a ΔG value of 1.53 eV (**Fig. R3**). In addition, the C-C coupling of two CO* species had a ΔG value of 0.96 eV and this reaction could not be facilitated by the electrode potential. Therefore, the Zn-pyrrolic-N_4_ should not be active for acetone production. For the other Zn-involved model, the (Cu-Zn)-pyrrolic-N_3_ site facilitated CO_2_ reduction to COOH* by reducing the ΔG to 0.73 eV, however, the non-electrochemical C-C coupling of two CO* species was highly endothermic with a ΔG value of 1.92 eV, as illustrated in **Fig. R3**. These additional DFT results revealed that the Zn species was not responsible for acetone production from CO_2_ reduction, confirming the experimental results that the trace amount of Zn impurity had a negligible effect on CO_2_ reduction on Cu-SA/NPC.

**Fig. R3** Gibbs free energy change for key elementary steps involved in CO_2_ reduction to acetone on different catalyst models including Cu-pyrrolic-N_4_, Zn-pyrrolic-N_4_, (Cu-Zn)-pyrrolic-N_3_ sites (This figure has been added into the revised Supporting Information as new **Fig. S29**).

**Table S1.** Inductively coupled plasma atomic emission spectrometry data for Cu and Zn contents in the prepared catalysts.

| Sample | ZIF-8 | NPC | Cu-doped ZIF-8 | Cu-SA/NPC | Cu-SA/NPC_Ar_ |
| --- | --- | --- | --- | --- | --- |
| Cu (wt %) | -- | -- | 0.12 | 0.59 | 0.60 |
| Zn (wt %) | 19.21 | 0.90 | 18.04 | 0.07 | 0.06 |

**Action:** New discussions were added into the revised Supporting Information in a new section of “**2.** **Impact of Zn on acetone production**” and “**3.3** **Zn-involved sites**”. We added the following sentence in the revised manuscript:

**Page 11** “*The trace amount of Zn impurity should have a negligible effect on acetone production from CO_2_ reduction on Cu-SA/NPC, as discussed in the* ***Supporting Information****.*”

**Pages S3-S4 of Supporting Information** “*According to the ICP-AES test (****Table S1****), Zn impurity existed in the NPC and Cu-SA/NPC. The Zn content of NPC was 0.90 wt %, which was 12 times higher than that of Cu-SA/NPC (0.07 wt %). However, the liquid products of CO_2_ reduction on NPC were HCOOH and CH_3_COOH (****Fig. S13****), demonstrating that Zn should not be responsible for the formation of CH_3_OH, C_2_H_5_OH and CH_3_COCH_3_. The Zn content of Cu-SA/NPC was only 0.07 wt %, and such low level of Zn on the catalysts could not combine with Cu nor change the selectivity toward CO_2_ reduction ^1^. In this work, the Cu atom was identified to be coordinated with N atom as measured by the EXAFS (****Fig.2b****), demonstrating that Zn was not coordinated with Cu. As shown in* ***Table S1****, Cu-SA/NPC and Cu-SA/NPC_Ar_ possessed similar Zn content of 0.07 wt % and 0.06 wt %, respectively, but the selectively of CH_3_OH, C_2_H_5_OH and CH_3_COCH_3_ production on these two catalysts was different. This also indicated that Zn should not be responsible for the formation of CH_3_OH, C_2_H_5_OH and CH_3_COCH_3_. Furthermore, the Cu content, BET surface area and pore structure (****Tables S1, S5*** *and* ***Figs. S19, S20****) of Cu-SA/NPC and Cu-SA/NPC_Ar_ catalysts were also similar, therefore, the disparate CO_2_ reduction activity of these two catalysts may be resulted from the content and type of N species. Furthermore, the influence of Zn impurity on acetone production from CO_2_ reduction on Cu-SA/NPC catalyst was also investigated by DFT calculations.****”***

**Pages S5-S6 of Supporting Information** “*To further investigate whether the Zn species had an impact on acetone production from CO_2_ reduction on Cu-SA/NPC, DFT calculations were performed on two types of catalyst models including Zn-pyrrolic-N_4_ and (Cu-Zn)-pyrrolic-N_3_ sites (****Figs. S29*** *and* ***S30****). The Gibbs free energy change for several key elementary steps were calculated including CO_2_ reduction to COOH* and then to CO*, together with two non-electrochemical C-C coupling reactions, and compared with that obtained on Cu-pyrrolic-N_4_. As shown in* ***Fig. S29****, on Cu-pyrrolic-N_4_ site without Zn, the first eletroreduction step of CO_2_ to COOH* had a ΔG value of 1.42 eV, and was identified to be the rate-limiting step for acetone formation according to* ***Fig. 5****. The two C-C coupling reactions of CO*+CO* and CO*+COCH_3_* were energetically exothermic with ΔG values of -1.23 and -0.92 eV, respectively, indicating a facile formation of acetone from CO_2_ reduction on Cu-pyrrolic-N_4_. For comparison, on Zn-pyrrolic-N_4_ without Cu, the first electroreduction step of CO_2_ to COOH* was energetically more endothermic with a ΔG value of 1.53 eV. In addition, the C-C coupling of two CO* species had a ΔG value of 0.96 eV and this reaction could not be facilitated by the electrode potential. Therefore, the Zn-pyrrolic-N_4_ was not active for acetone production. Although the (Cu-Zn)-pyrrolic-N_3_ facilitated CO_2_ reduction to COOH* by reducing the ΔG value to 0.73 eV, the non-electrochemical C-C coupling of two CO* was highly endothermic with a ΔG value of 1.92 eV, as illustrated in* ***Fig. S29****. These DFT results revealed that the Zn species was not responsible for acetone production from CO_2_ reduction, confirming the experimental results that the trace amount of Zn impurity had a negligible effect on CO_2_ reduction on Cu-SA/NPC.*”

***Question #3.*** *The active sites for CO_2_ reduction.*

**Response:** To clarify this question, we performed additional DFT calculations to examine other possible sites including different types of uncoordinated pyrrolic N sites, N=O sites, Zn-N sites and Zn-Cu-N sites. Together with the Cu-N_4_ sites reported in the manuscript, the DFT section “**Active sites identification and mechanisms of CO_2_ reduction on Cu-SA/NPC**” was reorganized to clarify the active sites for acetone production from CO_2_ production on Cu SA/NPC. According to the EXAFS fitting results, the Cu atom coordinated with N atom and the coordination number was measured to be 3.8 ± 0.2. The combined HAADF-STEM and EXAFS results indicated that the Cu species was atomically dispersed and was four-fold coordinated with N atoms in Cu SA/NPC. Based on these characterization results, we first examined CO_2_ reduction to acetone on the Cu-pyridinic-N_4_ and Cu-pyrrolic-N_4_ sites, and found that Cu coordinated with pyrrolic N species (Cu-pyrrolic-N_4_) was active for acetone production rather than Cu-pyridinic-N_4_ due to the energetically more favorable pathways leading to acetone formation on Cu-pyrrolic-N_4_ (**Fig. 5** in the manuscript). The DFT results were consistent with the structural prediction of Cu/N coordination environment from HAADF-STEM and EXAFS characterization and the N species analysis from XPS characterization that the Cu-SA/NPC had a higher content of pyrrolic N than that of Cu-SA/NPC_Ar_, which should be related to the high activity toward acetone production. To further confirm this, other possible sites were examined and compared with Cu-pyrrolic-N_4_. The DFT results revealed that Cu single atom coordinated with pyrrolic N species (Cu-pyrrolic-N_4_) was catalytically more active than the uncoordinated pyrrolic N specie based on the comparison on changes in the Gibbs free energy for pathways leading to acetone production from CO_2_ reduction on these sites (see **Fig. R4**). For N=O sites, several elementary steps involved in the paths were highly uphill in ΔG (see **Fig. R5**), therefore, the oxidized N sites should not be active for acetone formation. With regard to the impact of Zn, DFT calculations demonstrated that the Zn species was not responsible for acetone production from CO_2_ reduction based on the comparison on the free energy change for several key elementary steps involved in acetone production on Cu-pyrrolic-N_4_, Zn-pyrrolic-N_4_ and (Cu-Zn)-pyrrolic-N_3_ sites (see **Fig. R3**). Therefore, the combined experimental and DFT studies confirmed that the Cu single atom coordinated with four pyrrolic N (Cu-pyrrolic-N_4_) should be responsible for acetone production from CO_2_ reduction on Cu-SA/NPC, and were identified as active sites.

**Fig. R4** Free energy diagrams calculated at 0 eV for CO_2_ reduction to CH_3_COCH_3_ on uncoordinated pyrrolic-N_1_, uncoordinated pyrrolic-N_4_ and coordinated Cu-pyrrolic-N_4_ sites (the computational models were included in the figure) (This figure has been added into the revised Supporting Information as new **Fig. S24**).

**Fig. R5** Free energy diagrams calculated at potentials of 0 and -0.36 V for CO_2_ reduction to CH_3_COCH_3_ on **a** pyridinic-N=O and **b** pyrrolic-N=O sites. The two computational models were included in the figures (grey: C; red: O; blue: N; orange: Cu) (This figure has been added into the revised Supporting Information as new **Fig. S26**).

**Fig. R3** Gibbs free energy change for key elementary steps involved in CO_2_ reduction to acetone on different catalyst models including Cu-pyrrolic-N_4_, Zn-pyrrolic-N_4_, (Cu-Zn)- pyrrolic-N_3_ sites (This figure has been added into the revised Supporting Information as new **Fig. S29**).

**Action:** Some original text was modified for clarity on page 13 and page 15. The section “**Active sites identification and mechanisms of CO_2_ reduction on Cu-SA/NPC**” was reorganized and new discussions were added on pages 17-18 in the revised manuscript.

**Page 13** “*In view of CO_2_ reduction measurements and XPS results, CH_3_COCH_3_ production from CO_2_ reduction might be determined by the single atom Cu and the content of pyrrolic N. However, how these two species combined with each other was not confirmed based on experimental results and DFT calculations were conducted to identify the active sites of Cu-SA/NPC and to uncover the mechanisms for acetone production from CO_2_ reduction.*”

**Page 15** “*According to the combined HAADF-STEM and EXAFS results, the Cu species should be atomically dispersed and was four-fold coordinated with N atoms in Cu SA/NPC ^29^.*”

**Pages 17-18** “***Other possible sites examined and compared***

*To examine whether the uncoordinated pyrrolic N species was active toward acetone formation, DFT calculations were performed on two types of uncoordinated pyrrolic N models without Cu, as shown in* ***Figs. S24*** *and* ***S25****. The free energy diagrams for acetone production form CO_2_ reduction on uncoordinated* *pyrrolic-N_1_, uncoordinated* *pyrrolic-N_4_ and Cu-pyrrolic-N_4_ at 0 V potential were compared in* ***Fig. S24****. Clearly, the uncoordinated* *pyrrolic-N_4_ was not active toward acetone formation due to several uphill elementary steps other than CO_2_ reduction to COOH* (ΔG of 1.35 eV), such as COCOH* reduction to COC* (ΔG of 1.17 eV), COCH_2_* reduction to COCH_3_* (ΔG of 1.07 eV) and COCH_3_* coupling with CO* to form COCOCH_3_* (ΔG of 1.05 eV). For the uncoordinated* *pyrrolic-N_1_, although the first step of CO_2_ reduction to COOH* had a smaller ΔG value (0.75 eV) than that (1.42 eV) obtained on Cu-pyrrolic-N_4_, subsequent CO* formation from COOH* reduction had a larger ΔG of 1.24 eV. In addition, steps such as COCO* reduction to COCOH* and COHCOCH_3_* reduction to CCOCH_3_* were energetically endothermic with ΔG values of 0.70 and 0.97 eV, respectively, indicating that the uncoordinated* *pyrrolic-N_1_ did not have an advantage for acetone production. The comparison results in* ***Fig. S24*** *revealed that Cu single atom coordinated with pyrrolic N species (Cu-pyrrolic-N_4_) was catalytically more active than the uncoordinated pyrrolic N species, and should be responsible for the acetone production from CO_2_ reduction on Cu-SA/NPC. As shown in* ***Table S4****, other than the difference in the content of pyridinic N and pyrrolic N, the content of oxidized N was also different on Cu-SA/NPC and Cu-SA/NPC_Ar_. The experimental results on the N-doped porous carbon catalyst (NPC) without adding Cu showed no acetone formation from CO_2_ reduction, which suggested that the oxidized N should not be the active site for acetone production on Cu-SA/NPC. To further confirm this, DFT calculations of energetic pathways for CO_2_ reduction to acetone on pyridinic- and pyrrolic-N=O sites were performed (****Figs. S26-S28****), and detailed results were provided in the* ***Supporting Information****. Since several elementary steps involved in the paths were highly uphill in ΔG (****Fig. S26****), the oxidized N sites were not active for acetone formation. Furthermore, the influence of Zn impurity on acetone production from CO_2_ reduction on Cu-SA/NPC catalyst was also investigated by DFT calculations (****Figs. S29*** *and* ***S30****) which revealed that Zn was not responsible for acetone production from CO_2_ reduction.*

*These DFT results revealed that the active sites for acetone synthesis from CO_2_ reduction on Cu-SA/NPC were Cu-pyrrolic-N_4_, consistent with the structural prediction of Cu/N coordination environment from EXAFS characterization and the N species identified from XPS. For CO_2_ reduction to acetone, the formation of reaction intermediates required the synergy between Cu and coordinated pyrrolic N species (****Fig. 5b****), leading to a facile C-C coupling toward C_2_ and C_3_ species formation (****Fig. 5a****). The ICP results provided in* ***Table S1*** *revealed that the content of Cu dispersed into the Cu-SA/NPC catalyst was relatively small, and the EXAFS results (****Fig. 2b****) confirmed that the Cu coordinated with N atom. The combined experimental and DFT studies confirmed that the* *Cu single atom coordinated with four pyrrolic N (Cu-pyrrolic-N_4_) should be responsible for the acetone production from CO_2_ reduction on Cu-SA/NPC, and therefore were proposed as the active sites.*”

***Reviewer #2 (Remarks to the Author):*** *The authors have addressed all my previous* *comments properly, and I would recommend its publication.*

**Response:** We thank the reviewer for recommending publication of our manuscript in Nature Communications.

**Reviewer comments, third version:**

Reviewer #1 (Remarks to the Author):

The authors have extensively revised the manuscript, clarifying points previously raised and inconsistency. The manuscipt presents now some level of novelthy, specifically on the analysis of the Cu-Zn sites, and therefore I am able to recommend its publication as it is now.

**Author rebuttal, third version:**

*The authors have extensively revised the manuscript, clarifying points previously raised and inconsistency. The manuscipt presents now some level of novelthy, specifically on the analysis of the Cu-Zn sites, and therefore I am able to recommend its publication as it is now.*

**Response:** We thank the reviewer for recommending publication of our manuscript in Nature Communications.
